# Proteomics Analysis Reveals Serum Biomarkers Reflecting Joint Pain and Physical Limitations in Knee Osteoarthritis Before and After Joint Replacement Surgery

**DOI:** 10.1177/19476035261455413

**Published:** 2026-05-30

**Authors:** Anne-Mari Mustonen, Janne Tampio, Kristiina M. Huttunen, Petro Julkunen, Laura Säisänen, Lauri Karttunen, Jusa Reijonen, Amir Esrafilian, Tiina Kuningas, Atul Kumar, Heikki Kröger, Petteri Nieminen

**Affiliations:** 1 Institute of Biomedicine, School of Medicine, Faculty of Health Sciences, 205537 University of Eastern Finland, Kuopio, Finland; 2 Department of Environmental and Biological Sciences, Faculty of Science, Forestry and Technology, 205537 University of Eastern Finland, Joensuu, Finland; 3 School of Pharmacy, Faculty of Health Sciences, 205537 University of Eastern Finland, Kuopio, Finland; 4 Department of Technical Physics, Faculty of Science, Forestry and Technology, 205537 University of Eastern Finland, Kuopio, Finland; 5 Department of Clinical Neurophysiology, 60650 Kuopio University Hospital, Kuopio, Finland; 6 Institute of Clinical Medicine, School of Medicine, Faculty of Health Sciences, 205537 University of Eastern Finland, Kuopio, Finland; 7 Neuro Center, 60650 Kuopio University Hospital, Kuopio, Finland; 8 Department of Rehabilitation, 60650 Kuopio University Hospital, Kuopio, Finland; 9 Department of Bioengineering, Stanford University, Stanford, CA, USA; 10 UEF Bioinformatics Center, Biocenter Kuopio, Biocenter Finland, 205537 University of Eastern Finland, Kuopio, Finland; 11 Department of Orthopaedics, Traumatology and Hand Surgery, 60650 Kuopio University Hospital, Kuopio, Finland; 12 Kuopio Musculoskeletal Research Unit, 205537 University of Eastern Finland, Kuopio, Finland

**Keywords:** calcium homeostasis, coagulation, complement activation, extracellular matrix, immune response, inflammation, knee, osteoarthritis, pain, proteomics

## Abstract

**Objective:**

Osteoarthritis (OA) is an age-related musculoskeletal disorder lacking effective disease-modifying therapies and early diagnostic biomarkers. This study aimed to identify serum proteins that could indicate the occurrence of knee OA (KOA) and correlate with patients’ pain and functional impairment.

**Design/Methods:**

Fasting serum samples were collected from controls (n = 8) and patients with end-stage KOA at baseline (n = 8) and 12 months after total knee arthroplasty (n = 8). Proteomics analysis was conducted with liquid chromatography–mass spectrometry, followed by univariate and multivariate statistics and pathway analyses by MetaboAnalyst and STRING. Partial correlations were calculated with R, adjusted for sex, age, and body mass index, using a linear regression model.

**Results:**

151 proteins were upregulated and 5 proteins downregulated in baseline KOA compared to control. These proteins were linked to the complement system, immune response, coagulation, inflammatory response, calcium homeostasis, and extracellular matrix remodeling. Of these, complement factor I showed strong biomarker potential. Several proteins emerged as statistically significant predictors of cartilage loss, pain sensitivity, physical function, and corticospinal excitability. Systemic alterations persisted 12 months after surgery.

**Conclusion:**

Serum proteins may serve as biomarkers of KOA, reflecting disease-related immune, inflammatory, and tissue-remodeling processes that persist after joint replacement.

## Introduction

Osteoarthritis (OA) is a common age-associated musculoskeletal disorder and a major cause of physical disability in developed countries. The limited understanding of its pathophysiology hinders the discovery of effective pharmacological targets and, at present, available treatments are mainly focused on pain management and, eventually, joint replacement surgery.^1^ Sensitive, specific, and reliable biomarkers for diagnosing and monitoring the progression of OA during its pathogenic course remain elusive. Currently, biochemical marker candidates for diagnosis and prognosis are primarily associated with cartilage and bone structure and damage, inflammation, metabolism, and oxidative stress.^2^ There is a particular need for biomarkers that could detect early signs of articular cartilage degradation to identify pre-radiographic changes.

There has been considerable previous research on proteomics in the plasma, serum, and synovial fluid (SF) of OA patients. Several potential biomarkers for OA severity and/or progression have been identified,^3-7^ although they are not yet utilized in clinical settings. In serum and plasma, these biomarkers include, but are not limited to, cartilage acidic protein 1 (CRTAC1), cartilage oligomeric matrix protein (COMP), thrombospondin 4 (THBS4), interleukin (IL)-18 receptor 1, tumor necrosis factor (TNF) ligand superfamily member 14, fibrillin-1, vitamin D-binding protein, clusterin, lubricin, complement C3, inter-alpha-trypsin inhibitor heavy chain 1 (ITIH1), and S100 calcium-binding protein A6 (S100A6). In addition, enriched pathways include platelet activation and degranulation, extracellular matrix (ECM) interactions, and neutrophil degranulation.^8^

The current understanding of the etiology of OA pain is limited. Still, cytokines, neoepitopes, neuropeptides, cartilage-degrading proteinases, growth factors, and other proteins have been associated with OA pain.^9,10^ Unfortunately, most previous studies have not compared protein levels with perceived symptoms or OA patients’ ability to cope with the disease. According to Giordano et al,^11^ IL6, macrophage colony-stimulating factor 1, fibroblast growth factor-21, and TNF superfamily member 12 were significant independent predictors of subjective pain intensity in patients with knee OA (KOA). In Naili et al,^8^ ATPase 13 and hemoglobin subunit delta were upregulated and associated with patient-reported pain and symptoms, and peroxiredoxin-2 was associated with Knee Injury and Osteoarthritis Outcome Score pain. These previous studies suggest that circulating proteins may be associated with and potentially influence knee pain and joint function in OA.

The present study aimed to: (*i*) evaluate potential serum biomarkers using a proteomics approach in patients with KOA compared to healthy controls, and (*ii*) assess the associations between serum proteomics, pain symptoms, and performance-based joint function before and after total knee arthroplasty (TKA). A comprehensive dataset was assembled from individuals with KOA and from control participants, incorporating self-reported questionnaires, physiatric measures, magnetic resonance imaging (MRI)-based cartilage thickness measurements, and navigated transcranial magnetic stimulation (nTMS) tools to assess neuromuscular function. It was hypothesized that (*i*) serum proteomic profiles would distinguish controls from patients with KOA at baseline and following surgery, and that (*ii*) longitudinal alterations in circulating protein levels would relate to pain symptoms and joint function before and after TKA.

## Material and Methods

### Ethics and Subjects

**Figure 1.**
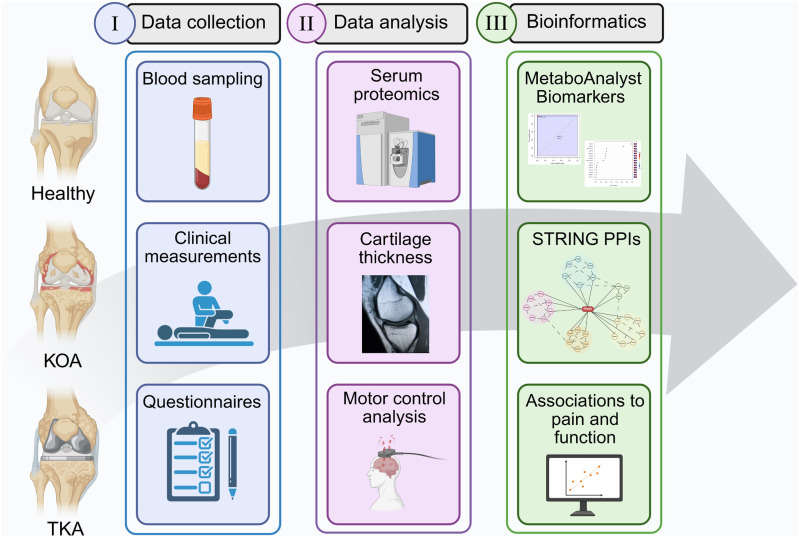
Illustration summarizing the study protocol, created in BioRender.com, Mustonen, A.-M. (2026) https://BioRender.com/9jtz0el. KOA = knee osteoarthritis, TKA = total knee arthroplasty, STRING = Search Tool for the Retrieval of Interacting Genes/Proteins, PPI = protein–protein interaction

This study is in accordance with the Declaration of Helsinki, and ethical approval was obtained from the Regional Medical Research Ethics Committee of Eastern Finland Collaborative Area (decision #140/2017, amendment 8/2020). All subjects provided written informed consent prior to study participation to donate their blood samples for research purposes. The study protocol is shown in Figure 1. Eight patients (3 men, 5 women) with end-stage primary KOA who underwent TKA at Kuopio University Hospital in 2020–2022 and 8 healthy volunteers (3 men, 5 women) with no clinical history of joint disorders were included in the study.

Inclusion criteria for patients were as follows: age 45–70 years; radiographically verified KOA (Kellgren–Lawrence grade 2–4); tibiofemoral joint pain and functional limitations on >50% of days over the last 12 months; and no diagnoses for abnormal mental status. Patients were excluded if they had severe knee pain caused by other conditions, such as trauma, infections, or autoimmune diseases; limited range of motion (ROM; severe flexion or extension deficit as determined by an experienced clinician during outpatient assessment) or substantial instability requiring, e.g., hinged knee arthroplasty; neurological or metabolic diseases; continuous use of central nervous system agents; active malignancies; inflammatory arthritis; earlier restorative surgery of the knee; pregnancy; metal objects or implants in the body if not compatible with MRI scanners or nTMS, cardiac pacemaker; body mass index (BMI) >33 kg/m^2^, or thigh circumference >52 cm. Patients requiring expedited surgery because of uncontrollable pain or avascular necrosis of the knee were also excluded.

Gender, age, body mass, height, BMI, and medication were recorded. There were no differences in the sex ratios between the study groups, but KOA patients were expectedly older (32 ± 3 vs. 62 ± 2 years, Student’s t-test, p <0.001) and had a higher average BMI than controls (26.0 ± 1.01 vs. 31.9 ± 0.51 kg/m^2^, Student’s t-test, p <0.001).

### Blood Sampling

Venous blood was drawn from control participants at baseline (n = 8) and KOA patients prior to TKA (n = 8) following an overnight fast (average duration 12 h, min 7 h, max 15 h), using BD Vacutainer Clot Activator tubes (BD, Belliver Industrial Estate, Plymouth, UK). KOA patients were also sampled 12 months post-TKA (n = 8), while controls were not sampled at follow-up. After collection, samples were allowed to clot at room temperature (RT) for 30 min and then centrifuged at 2500 × *g* for 15 min at RT.^12^ The resulting supernatant was transferred to a new tube and subjected to a second centrifugation under the same conditions. The platelet-poor serum was aliquoted and stored at −80°C until further analysis.

### Cartilage Thickness Measurement

Articular cartilage thickness in the principal load-bearing regions of the medial tibia and femur, areas most susceptible to cartilage degradation, was measured from MRI scans (Philips Achieva 3.0 T X, Philips, Eindhoven, the Netherlands; or Siemens MAGNETOM Vida, Siemens Healthcare, Erlangen, Germany) using an automated pipeline implemented in Python. This pipeline was based on tissue geometries obtained using a deep-learning-based segmentation tool, nnU-Net.^13^

### Pain Assessment and Physical and Neuromuscular Evaluations

Clinical measurements were conducted during outpatient visits and included ROM (flexion, extension) and physical function tests recommended by the Osteoarthritis Research Society International (30-s chair-stand test, 4 × 10 m fast-paced walk test, and 12-step stair-climb test), as previously described.^14^ The ROM was assessed using standard goniometric methods with the patient in the supine position. Pain and sensation were evaluated using the visual analog scale, pressure pain threshold (PPT), thermal detection and heat pain thresholds, and two-point discrimination (TPD). Self-reported questionnaires included the Western Ontario and McMaster Universities Osteoarthritis Index, painDETECT, RAND-36 measure of health-related quality of life, Beck Depression and Anxiety Inventories, and Pain Self-Efficacy Questionnaire. These questionnaires were not obtained from the controls. nTMS was utilized to test neuromuscular function (NBS 4.3 research version, Nexstim Plc, Helsinki, Finland), specifically to determine the resting motor threshold (rMT) of the *tibialis anterior* muscle, as described previously.^14^

### Non-Targeted Peptide Analysis of Serum Proteins

The total protein concentrations of serum samples were quantified using the Pierce BCA Protein Assay Kit (Thermo Fisher Scientific, Waltham, MA, USA) with the Hidex Sense microplate reader (Hidex, Turku, Finland). Based on the protein concentrations, serum samples equivalent to 50 µg of proteins were digested for peptide analysis by liquid chromatography–mass spectrometry (LC–MS).

The protein samples (50 µg) were solubilized in 0.5 M Tris–HCl buffer (pH 8.5) containing 7 M guanidine hydrochloride and 10 mM ethylenediaminetetraacetic acid. Proteins were reduced with dithiothreitol (1:50, w/w) for 1 h, and alkylated with iodoacetamide (1:20, w/w) for 1 h in the dark. The alkylated proteins were collected as a precipitated pellet by extraction with methanol, chloroform, and water (4:1:3, v/v/v), combined with centrifugation at 18,000 × *g* for 5 min at 4°C. The protein pellets were resolubilized using 6 M urea in 0.1 M Tris–HCl (pH 8.5), followed by a 5-fold dilution with 0.1 M Tris–HCl. The proteins were digested into peptides using Trypsin/Lys-C Mix (1:25, w/w; Promega Biotech AB, Nacka, Sweden) and 0.05% ProteaseMax (Promega Biotech AB) for 18 h at 37°C. The digestion was terminated by acidification with 10% formic acid. The digested peptides were diluted to 0.5 µg/µl with MilliQ water and centrifuged at 15,000 × *g* for 10 min at 10°C before the LC–MS analysis.

The digested peptides were analyzed in data-dependent acquisition (DDA) mode according to the previously published method,^15^ with an extended chromatographic separation. Briefly, digested peptide samples equal to 10 μg of intact proteins were separated with the ultra-high performance LC (Vanquish Flex UHPLC system, Thermo Fisher Scientific, Bremen, Germany) using the AdvanceBio Peptide Map column (2.1 × 250 mm, 2.7 μm; Agilent Technologies, Santa Clara, CA, USA) and a mobile phase of 0.1% formic acid in both water (eluent A) and acetonitrile (eluent B). The flow rate was 0.3 ml/min with the following gradient: 0–2 min: 2% B → 7%, 2–5 min: 7% B, 5–50 min: 7% B → 30%, 50–83 min: 30% B → 45%, 83–83.5 min: 45% B → 80%, 83.5–85.5 min: 80% B, 85.5–86 min: 80% B → 2%, 86–90 min: 2% B. The LC system was coupled with a high-resolution MS (Q-Exactive Classic mass spectrometry, Thermo Fisher Scientific). Full-scan MS spectra were recorded between 1–86 min with the *m/z* range of 395–1247, and the 10 most intense multiply charged ions (z ≥ 2) were selected for MS/MS microscans with the *m/z* range of 200–2000.

The collected MS spectra were processed with the Skyline software (*v*25.1) using the DDA peptide search.^16^ A spectral library was built from the acquired data using the UniProt reference proteome database for *Homo sapiens* (entry UP000005640; version downloaded May 8, 2025) by MS Amanda. The acquired spectra were matched with a mass tolerance of 5 ppm for precursor ions and 20 ppm for fragment ions (b and y ions). The project-specific library was used to search the identified peptides in the sample data with the false discovery rate (FDR) threshold of 0.05 for peptides with lengths of 7–30 amino acids, with a maximum of 2 missed cleavages after the trypsin digestion. Unique peptides were annotated to corresponding proteins for identification, whereas shared peptides were used to form protein groups. The relative protein abundances between sample groups were compared based on the summed intensities of the identified peptides for each protein or protein group. A comprehensive list of identified proteins and their abundances in each subject is presented in Supplemental Table S1.

### Statistical and Bioinformatic Analyses

Statistical analyses were conducted using the IBM SPSS Statistics *v*29.0.2.0 (IBM, Armonk, NY, USA) and Microsoft Excel (Microsoft, Redmond, WA, USA). Baseline differences in age and BMI were assessed using the independent-samples Student’s t-test, while the sex ratios were compared using the Fisher’s exact test. A p-value <0.05 was considered statistically significant. Differences in protein abundances between the study groups were evaluated using the independent-samples Student’s t-test. The comparisons were performed between control participants and KOA patients at baseline, between KOA patients at baseline and at 12 months post-TKA, and between control participants and KOA patients at 12 months post-TKA. This approach allowed us to assess both disease-specific changes and the effects of surgical intervention. To control the FDR for multiple comparisons, the p-values were adjusted using the Benjamini–Hochberg procedure.^17^

Partial correlation analysis was performed using the R software *v*4.5.1 (R Core Team, R Foundation for Statistical Computing, Vienna, Austria). To account for confounding factors, protein values and clinical variables were first adjusted by regressing out sex, age, and BMI using linear models (lm()). Residuals obtained from these models were extracted using the residuals() function and used as input for the subsequent correlation analysis. Pearson correlation coefficients between the residualized protein and clinical variables were computed using the function cor() with method = “pearson”, and statistical significance of individual protein–clinical variable associations was assessed with cor.test(method = “pearson”). Multiple testing correction was applied to the resulting p-values using the Benjamini–Hochberg procedure (p.adjust(method = “fdr”)).^17^ The most significant partial correlations between protein and clinical variables were visualized using the ggcorrplot package (*v*0.1.4.1) in R. For plotting, the results were filtered using a cut-off of FDR-adjusted p-value <0.05 and ranked by the adjusted p-value. Correlation coefficients were reshaped into a protein × clinical variable matrix using reshape2::acast() (*v*1.4.4.), and correlation plots were generated using the function ggcorrplot(). Scatter plots of each significant protein–clinical variable pair (FDR-adjusted p-value <0.05 in the analyses of either groups 1 and 2 or groups 2 and 3) were generated using the ggplot2 package (*v*4.0.0) in R. Individual samples were plotted as protein abundance vs. clinical variable values using ggplot() function, and linear regression lines were overlaid using ordinary least squares with geom_smooth(method = “lm”) (Supplemental Fig. S1–S2). Based on the differential expression of the proteins, volcano plots were generated using the R *v*4.5.3 by plotting Log2 fold change (FC) on the X-axis and –log10 of p-value on the Y-axis. The top 10 upregulated proteins, if significant (FDR <0.05), are labeled on the plot.

MetaboAnalyst *v*6.0, an open-access web-based platform for comprehensive pathway analysis in metabolomics research (https://www.metaboanalyst.ca), was used to identify potential therapeutic targets for KOA. The protein values were normalized by median, followed by the overall data normalization by log transformation. Baseline KOA was compared to controls, and post-surgical KOA was compared to baseline KOA and to controls. Candidate biomarkers were selected based on variable importance in projection (VIP) values and loading plots from the partial least squares discriminant analysis (PLS-DA) model. The diagnostic performance of each biomarker was assessed by calculating the area under the receiver operating characteristic (ROC) curve (AUC). Sensitivity and specificity values were also calculated to assess the potential clinical applicability of the biomarkers for distinguishing between the defined groups.

The obtained differentially expressed proteins (DEPs) were imported into the Search Tool for the Retrieval of Interacting Genes/Proteins (STRING) *v*12.0,^18^ a comprehensive online resource (https://string-db.org/) designed to evaluate known and predicted protein–protein interactions (PPIs). These include direct (physical) and indirect (functional) associations between proteins, stemming from computational prediction, knowledge transfer between organisms, and interactions aggregated from other (primary) databases. The research species was set to *Homo sapiens*, the minimum required interaction score of 0.400 (medium confidence) was selected, and PPI network diagrams were constructed separately for baseline KOA vs. control, post-surgical KOA vs. baseline KOA, and post-surgical KOA vs. control. To gain further insight into the functional roles and biological significance of the proteins involved in each comparison, enrichment analyses were performed within the STRING platform. These included Gene Ontology (GO) classifications of biological processes and molecular functions, as well as pathway enrichment analysis using the Kyoto Encyclopedia of Genes and Genomes (KEGG) database.

## Results

### Effects of KOA and TKA on Serum Proteomics

A total of 804 proteins were identified in the serum samples (Supplemental Table S1). There were 156 DEPs between controls and KOA patients at baseline, of which 60 remained significant after applying the Benjamini–Hochberg procedure. Between KOA at baseline and 12 months after TKA, 116 proteins were differentially expressed with 1 remaining significant after the FDR correction. When controls were compared with KOA patients 12 months after TKA, there were 258 DEPs, of which 169 remained significant after the FDR correction.

### Control vs. Baseline KOA

**Figure 2.**
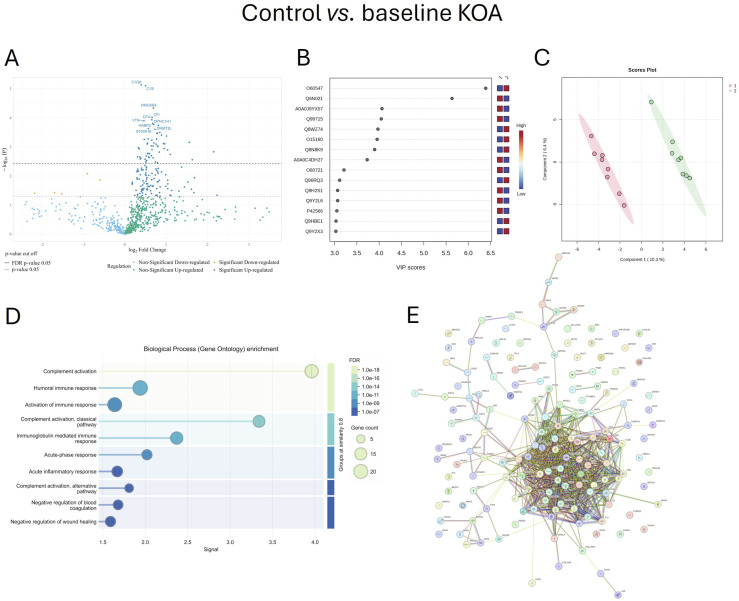
Serum proteins involved in biochemical pathways in knee osteoarthritis (KOA) compared to control. Volcano plot (A), variable importance in projection (VIP) scores (B), partial least squares discriminant analysis score plot (C), Gene Ontology biological process enrichment analysis (D), and protein–protein interaction (PPI) network (E). Network (E) nodes represent proteins, and edges represent PPIs. Colored nodes indicate query proteins and first shell of interactors. The colored edges connecting proteins indicate known interactions in turquoise (from curated databases) and purple (experimentally determined). Predicted interactions are shown in green (gene neighborhood), red (gene fusions), and dark blue (gene co-occurrence). Yellow indicates text mining, black co-expression, and light blue protein homology. Groups: 1 = control, 2 = baseline KOA, FDR = false discovery rate. Proteins are labeled using UniProt accession numbers in B and gene symbols in A and E, the full names of the proteins and their UniProt accession numbers are listed in Supplemental Table S1

Baseline KOA samples showed 151 proteins to be upregulated, and 5 proteins downregulated compared to control sera. The top 30 KOA-associated proteins included, for instance, several complement-related proteins, ECM-associated glycoproteins (vitronectin [VTN], fibronectin), proteins involved in calcium homeostasis (S100A16, ryanodine receptor 3, calcium homeostasis modulator protein 3, alpha-2-HS-glycoprotein), coagulation-related proteins (hyaluronan-binding protein 2 [HABP2], kininogen-1, coagulation factor XI), and proteins involved in lipid metabolism (apolipoprotein E [APOE], adipocyte plasma membrane-associated protein). The baseline KOA-linked proteins with the highest significance are listed in Table 1, and the volcano plot highlights the most significantly upregulated proteins (Figure 2A).

**Table 1.** The Top 30 Differentially Expressed Proteins in Serum of Patients With Knee Osteoarthritis Compared to Controls (n = 8/Group) Sorted by p-value Ranking

Protein name	UniProt #	Gene	*P* (t-test)	B–H	Simple FC	Log2FC
Complement C1q subcomponent subunit A	P02745	C1QA	7.32 × 10^–6^	6.21 × 10^–5^	1.32	0.40
Complement C1s subcomponent	P09871	C1S	7.90 × 10^–6^	0.000124	1.42	0.51
HMG domain-containing protein 4	Q9UGU5	HMGXB4	4.58 × 10^–5^	0.000186	1.62	0.70
Complement factor I	P05156	CFI	9.55 × 10^–5^	0.000248	1.58	0.66
Complement factor H	P08603	CFH	0.000116	0.000311	1.58	0.66
Vitronectin	P04004	VTN	0.000127	0.000373	1.38	0.47
Hyaluronan-binding protein 2	Q14520	HABP2	0.000156	0.000435	1.60	0.68
Cytoplasmic dynein 1 heavy chain 1	Q14204	DYNC1H1	0.000168	0.000497	1.63	0.71
DNA (cytosine-5)-methyltransferase 3-like	Q9UJW3	DNMT3L	0.000182	0.000559	1.76	0.81
Protein S100-A16	Q96FQ6	S100A16	0.000231	0.000621	1.49	0.57
Ryanodine receptor 3	Q15413	RYR3	0.000248	0.000683	1.66	0.73
Bcl-2-associated transcription factor 1	Q9NYF8	BCLAF1	0.000318	0.000745	1.80	0.85
Protein SCAF11	Q99590	SCAF11	0.000331	0.000807	1.73	0.79
Neuferricin	Q8WUJ1	CYB5D2	0.000331	0.000870	1.73	0.79
Complement component C6	P13671	C6	0.000345	0.000932	1.71	0.78
FH1/FH2 domain-containing protein 1	Q9Y613	FHOD1	0.000392	0.000994	1.85	0.89
Calcium homeostasis modulator protein 3	Q86XJ0	CALHM3	0.000422	0.001056	2.02	1.01
Insulin-like growth factor-binding protein complex acid labile subunit	P35858	IGFALS	0.000528	0.001118	1.48	0.57
Ribosome production factor 2 homolog	Q9H7B2	RPF2	0.000535	0.001180	1.71	0.77
Kininogen-1	P01042	KNG1	0.000555	0.001242	1.39	0.47
Serum amyloid P-component	P02743	APCS	0.000586	0.001304	1.58	0.66
Peroxynitrite isomerase THAP4	Q8WY91	THAP4	0.000696	0.001366	2.95	1.56
Apolipoprotein E	P02649	APOE	0.000726	0.001429	1.63	0.71
Complement C5	P01031	C5	0.000736	0.001491	1.47	0.55
Adipocyte plasma membrane-associated protein	Q9HDC9	APMAP	0.000768	0.001553	1.92	0.94
Small ribosomal subunit protein mS34	P82930	MRPS34	0.000787	0.001615	1.96	0.97
Complement C3	P01024	C3	0.000794	0.001677	1.46	0.55
Fibronectin	P02751	FN1	0.000801	0.001739	1.69	0.76
C4b-binding protein alpha chain	P04003	C4BPA	0.000916	0.001801	1.56	0.64
Coagulation factor XI	P03951	F11	0.000923	0.001863	1.73	0.79

B–H = Benjamini–Hochberg correction critical value

The highest VIP scores for baseline KOA vs. control, determined by MetaboAnalyst, were observed for serum GDP-mannose 4,6 dehydratase (GMDS), methylcytosine dioxygenase TET2, melanoma-associated antigen B6B, collagen alpha-1(XII) chain, and cortactin-binding protein 2 (Figure 2B). Components 1–2 clearly separated control and KOA baseline samples in the PLS-DA (Figure 2C). The ROC analysis identified several strong biomarker candidates for baseline KOA. Complement factor CFI had the greatest biomarker potential, with an AUC of 1.000 and both sensitivity and specificity of 1.000. It was followed by HMG domain-containing protein 4, melanoma-associated antigen B6B (AUC 0.969 for both, sensitivity 0.9–1.0, specificity 0.9–1.0), cytoplasmic dynein 1 heavy chain 1, frataxin, mitochondrial, DNA (cytosine-5)-methyltransferase 3-like (DNMT3L), and PDZ domain-containing protein 4 (AUC 0.953, sensitivity 0.9–1.0, specificity 0.9–1.0).

The biological processes identified by the STRING GO analysis included, for instance, complement activation, complement activation (classical pathway), immunoglobulin-mediated immune response, acute-phase response, and humoral immune response (Figure 2D). In addition, we identified several processes related to coagulation, inflammatory response, immune response, wound healing, lipoprotein, cholesterol, and triglyceride metabolism, as well as oxidative stress. GO molecular functions, describing the biochemical activity of a gene product, involved endopeptidase inhibitor activity, peptidase regulator activity, serine-type endopeptidase inhibitor activity, serine-type endopeptidase activity, enzyme inhibitor activity, glycosaminoglycan binding, complement binding, heparin binding, cholesterol/lipid transfer activity, and antioxidant activity, among other functions. KEGG pathways highlighted, for instance, complement and coagulation cascades, systemic lupus erythematosus, and cholesterol metabolism. The PPI network contained 151 nodes, the number of edges was 761, the average node degree was 10.1, the average local clustering coefficient was 0.467, and the PPI enrichment p-value was <1.0 × 10^-16^ (Figure 2E), indicating that these proteins formed functionally interconnected and biologically relevant networks and not only random associations. The top hub proteins in the KOA PPI network included VTN, alpha-2-HS-glycoprotein, complement C3, plasminogen, prothrombin, and alpha-1-antitrypsin, which are central to complement and coagulation cascades, ECM remodeling, and inflammatory regulation pathways, these results being consistent with the GO enrichment analysis. Together, the findings highlight the involvement of immune–coagulation interactions, inflammatory pathways, lipid metabolism, and oxidative stress in KOA pathophysiology.

### Baseline KOA vs. Post-surgical KOA

**Figure 3.**
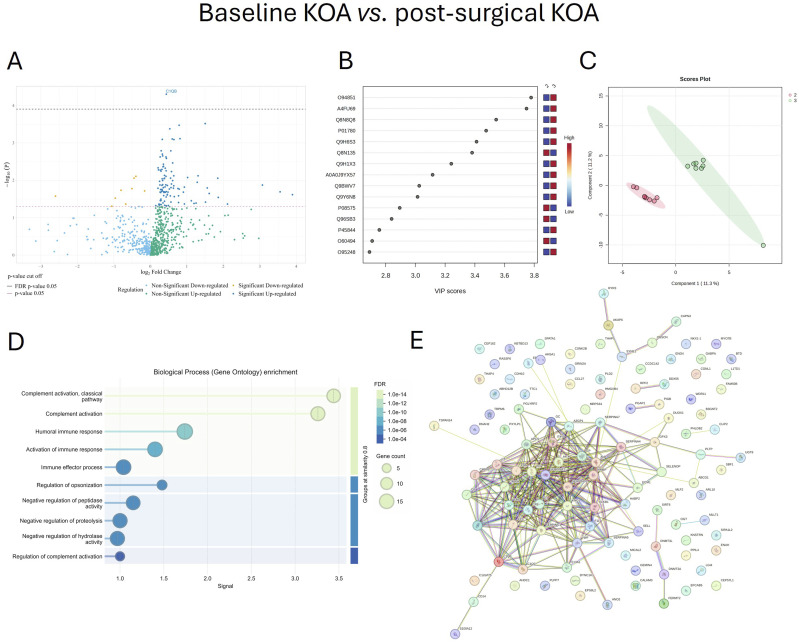
Serum proteins involved in biochemical pathways in post-surgical knee osteoarthritis (KOA) compared to baseline KOA. Volcano plot (A), variable importance in projection (VIP) scores (B), partial least squares discriminant analysis score plot (C), Gene Ontology biological process enrichment analysis (D), and protein–protein interaction (PPI) network (E). Network (E) nodes represent proteins, and edges represent PPIs. Colored nodes indicate query proteins and first shell of interactors. The colored edges connecting proteins indicate known interactions in turquoise (from curated databases) and purple (experimentally determined). Predicted interactions are shown in green (gene neighborhood), red (gene fusions), and dark blue (gene co-occurrence). Yellow indicates text mining, black co-expression, and light blue protein homology. Groups: 2 = baseline KOA, 3 = post-surgical KOA (12 months), FDR = false discovery rate. Proteins are labeled using UniProt accession numbers in B and gene symbols in A and E, the full names of the proteins and their UniProt accession numbers are listed in Supplemental Table S1

When baseline KOA samples were compared to sera 12 months after TKA, 107 proteins were upregulated and 9 downregulated post-surgery. The top 30 post-surgical KOA-associated proteins included several complement-related proteins, proteins involved in coagulation (coagulation factor XI, plasma kallikrein, heparin cofactor 2), ECM-associated proteins (fermitin family homolog 2 [FERMT2], ITIH2), as well as vitamin D-binding protein and glutathione peroxidase 3, a key antioxidant enzyme (Table 2). The volcano plot emphasizes the most significantly upregulated protein, complement C1q subcomponent subunit B (Figure 3A).

**Table 2.** The Top 30 Differentially Expressed Proteins in Serum of Patients With Post-surgical Knee Osteoarthritis (KOA) Compared to Baseline KOA (n = 8/Group) Sorted by p-value Ranking

Protein name	UniProt #	Gene	P (t-test)	B–H	Simple FC	Log2FC
Complement C1q subcomponent subunit B	P02746	C1QB	4.98 × 10^–5^	6.21 × 10^–5^	1.39	0.48
Overexpressed in colon carcinoma 1 protein	Q8TAD7	OCC1	0.000304	0.000124	3.10	1.63
A-kinase anchor protein 6	Q13023	AKAP6	0.000337	0.000186	1.85	0.88
Centrosomal protein CEP57L1	Q8IYX8	CEP57L1	0.000420	0.000248	1.45	0.53
Fermitin family homolog 2	Q96AC1	FERMT2	0.000771	0.000311	1.82	0.87
Spermatogenesis-associated protein 1	Q5VX52	SPATA1	0.000786	0.000373	1.48	0.56
Complement C2	P06681	C2	0.000806	0.000435	1.28	0.35
Coagulation factor XI	P03951	F11	0.000813	0.000497	1.62	0.69
Vitamin D-binding protein	P02774	GC	0.001626	0.000559	1.23	0.30
DNA (cytosine-5)-methyltransferase 3A	Q9Y6K1	DNMT3A	0.001632	0.000621	1.55	0.63
Plasma kallikrein	P03952	KLKB1	0.001998	0.000683	1.27	0.34
Kallistatin	P29622	SERPINA4	0.002224	0.000745	1.27	0.34
Inter-alpha-trypsin inhibitor heavy chain H2	P19823	ITIH2	0.002372	0.000807	1.20	0.26
C4b-binding protein beta chain	P20851	C4BPB	0.002374	0.000870	1.36	0.45
HMG domain-containing protein 4	Q9UGU5	HMGXB4	0.002595	0.000932	1.42	0.50
DNA (cytosine-5)-methyltransferase 3-like	Q9UJW3	DNMT3L	0.003434	0.000994	1.68	0.75
Complement C5	P01031	C5	0.003713	0.001056	1.33	0.41
Protein AMBP	P02760	AMBP	0.004230	0.001118	1.34	0.42
Cingulin-like protein 1	Q0VF96	CGNL1	0.004347	0.001180	1.30	0.37
Complement C1q subcomponent subunit C	P02747	C1QC	0.004641	0.001242	1.17	0.22
Heparin cofactor 2	P05546	SERPIND1	0.004751	0.001304	1.31	0.38
Complement component C8 alpha chain	P07357	C8A	0.005113	0.001366	1.28	0.36
Albumin	P02768	ALB	0.005256	0.001429	1.18	0.24
Complement C1s subcomponent	P09871	C1S	0.005349	0.001491	1.21	0.28
Ribosome production factor 2 homolog	Q9H7B2	RPF2	0.005561	0.001553	1.44	0.52
Protein enabled homolog	Q8N8S7	ENAH	0.005695	0.001615	1.23	0.30
Myeloid leukemia factor 2	Q15773	MLF2	0.005848	0.001677	1.61	0.68
Glutathione peroxidase 3	P22352	GPX3	0.007377	0.001739	1.33	0.41
Epidermal growth factor receptor kinase substrate 8-like protein 2	Q9H6S3	EPS8L2	0.007573	0.001801	2.29	1.20
Peroxynitrite isomerase THAP4	Q8WY91	THAP4	0.007577	0.001863	1.73	0.79

B–H = Benjamini–Hochberg correction critical value

The highest VIP scores for post-surgical KOA vs. baseline KOA were observed for serum [F-actin]-monooxygenase MICAL2, EF-hand calcium-binding domain-containing protein 5, cytochrome c oxidase assembly protein COX18, mitochondrial, immunoglobulin heavy variable 3-7, and epidermal growth factor receptor kinase substrate 8-like protein 2 (Figure 3B). In the PLS-DA, components 1–2 clearly separated the baseline and post-surgical KOA samples (Figure 3C). The ROC analysis showed that zinc finger and BTB domain-containing protein 46 and overexpressed in colon carcinoma 1 protein had the greatest biomarker potential for post-surgical KOA, with an AUC of 0.984, sensitivity of 0.9–1.0, and specificity of 0.9–1.0. They were followed by FERMT2, calpain-3 (CAPN3), NAD-dependent protein deacylase sirtuin-6, DNMT3L, and Kelch-like protein 32 (AUC 0.938–0.953, sensitivity 0.9, specificity 0.9–1.0).

Based on the STRING analyses, complement activation (classical pathway), complement activation, humoral immune response, regulation of opsonization, activation of immune response, and blood coagulation, among others, were included in GO biological processes (Figure 3D). GO molecular functions involved endopeptidase inhibitor activity, serine-type endopeptidase inhibitor activity, complement binding, glycosaminoglycan binding, and serine-type endopeptidase activity. The top KEGG pathways included complement and coagulation cascades and systemic lupus erythematosus, among other pathways. In the PPI network, there were 112 nodes and 318 edges, the average node degree was 5.68, the average local clustering coefficient was 0.391, and the PPI enrichment p-value was <1.0 × 10^-16^ (Figure 3E). The top hub proteins in the post-surgical KOA PPI network were albumin, heparin cofactor 2, complement C3, CFI, kininogen-1, and complement factor B, which are involved in complement activation, coagulation and kallikrein–kinin system pathways, as well as in the regulation of inflammation. Together, our findings indicate that there are remaining alterations in immune response and coagulation cascades in KOA patients 12 months after TKA.

### Post-Surgical KOA vs. Control

**Figure 4.**
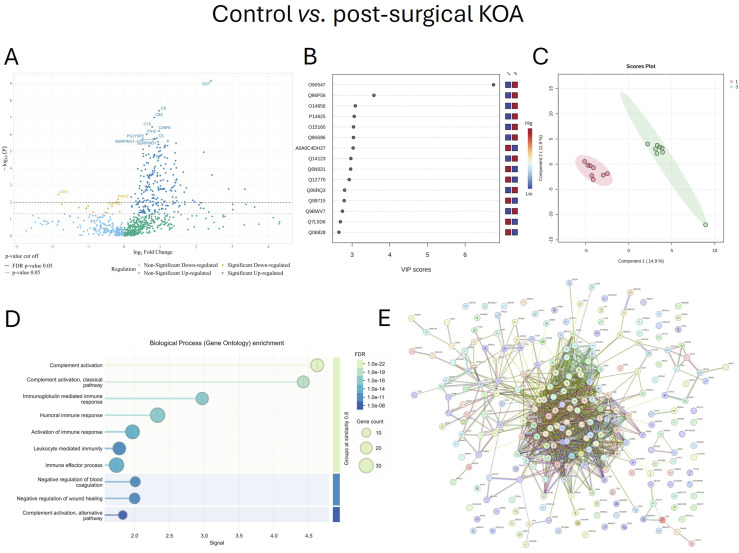
Serum proteins involved in biochemical pathways in post-surgical knee osteoarthritis (KOA) (12 months) compared to control. Volcano plot (A), variable importance in projection (VIP) scores (B), partial least squares discriminant analysis score plot (C), Gene Ontology biological process enrichment analysis (D), and protein–protein interaction (PPI) network (E). Network (E) nodes represent proteins, and edges represent PPIs. Colored nodes indicate query proteins and first shell of interactors. The colored edges connecting proteins indicate known interactions in turquoise (from curated databases) and purple (experimentally determined). Predicted interactions are shown in green (gene neighborhood), red (gene fusions), and dark blue (gene co-occurrence). Yellow indicates text mining, black co-expression, and light blue protein homology. Groups: 1 = control, 3 = post-surgical KOA (12 months), FDR = false discovery rate. Proteins are labeled using UniProt accession numbers in B and gene symbols in A and E, the full names of the proteins and their UniProt accession numbers are listed in Supplemental Table S1

Compared to control sera, post-surgical KOA samples showed 245 upregulated and 13 downregulated proteins. The top 30 post-surgical KOA-associated proteins included several complement-related proteins, coagulation-associated proteins (plasma protease C1 inhibitor, heparin cofactor 2, coagulation factor XI, HABP2, alpha-2-antiplasmin [SERPINF2], plasma kallikrein, vitamin K-dependent protein S), ITIH2, glutathione peroxidase 3, and hemopexin (Table 3). The volcano plot illustrates proteins with the most significant up- and downregulation (Figure 4A).

**Table 3.** The Top 30 Differentially Expressed Proteins in Serum of Patients With Post-surgical Knee Osteoarthritis Compared to Control (n = 8/Group) Sorted by p-value Ranking

Protein name	UniProt #	Gene	P (t-test)	B–H	Simple FC	Log2 FC
UDP-N-acetylglucosamine--peptide N-acetylglucosaminyltransferase 110 kDa subunit	O15294	OGT	6.91 × 10^–10^	6.21 × 10^–5^	5.44	2.44
Complement component C9	P02748	C9	4.05 × 10^–8^	0.000124	1.97	0.98
Complement component C8 alpha chain	P07357	C8A	1.01 × 10^–7^	0.000186	1.84	0.88
Complement C1s subcomponent	P09871	C1S	3.63 × 10^–7^	0.000248	1.72	0.79
C4b-binding protein alpha chain	P04003	C4BPA	6.36 × 10^–7^	0.000311	1.97	0.98
Inter-alpha-trypsin inhibitor heavy chain H2	P19823	ITIH2	9.96 × 10^–7^	0.000373	1.53	0.61
Complement C5	P01031	C5	1.71 × 10^–6^	0.000435	1.95	0.96
Plasma protease C1 inhibitor	P05155	SERPING1	1.82 × 10^–6^	0.000497	1.79	0.84
Heparin cofactor 2	P05546	SERPIND1	1.88 × 10^–6^	0.000559	1.78	0.83
N-acetylmuramoyl-L-alanine amidase	Q96PD5	PGLYRP2	1.90 × 10^–6^	0.000621	1.43	0.52
FH1/FH2 domain-containing protein 1	Q9Y613	FHOD1	2.49 × 10^–6^	0.000683	2.38	1.25
Protein AMBP	P02760	AMBP	2.79 × 10^–6^	0.000745	2.00	1.00
Coagulation factor XI	P03951	F11	3.26 × 10^–6^	0.000807	2.80	1.49
Tetratricopeptide repeat protein 1	Q99614	TTC1	3.99 × 10^–6^	0.000870	1.93	0.95
HMG domain-containing protein 4	Q9UGU5	HMGXB4	4.48 × 10^–6^	0.000932	2.29	1.20
Ribosome production factor 2 homolog	Q9H7B2	RPF2	4.60 × 10^–6^	0.000993	2.45	1.29
Complement C2	P06681	C2	4.92 × 10^–6^	0.001056	1.65	0.72
Complement component C6	P13671	C6	5.39 × 10^–6^	0.001118	2.33	1.22
A-kinase anchor protein 6	Q13023	AKAP6	5.92 × 10^–6^	0.001180	2.31	1.21
Glutathione peroxidase 3	P22352	GPX3	6.03 × 10^–6^	0.001242	1.92	0.94
Protein SCAF11	Q99590	SCAF11	6.22 × 10^–6^	0.001304	2.08	1.05
Neuferricin	Q8WUJ1	CYB5D2	6.22 × 10^–6^	0.001366	2.08	1.05
Complement C1q subcomponent subunit C	P02747	C1QC	7.63 × 10^–6^	0.001429	1.45	0.54
Complement factor I	P05156	CFI	9.44 × 10^–6^	0.001491	2.06	1.04
Hyaluronan-binding protein 2	Q14520	HABP2	1.05 × 10^–5^	0.001553	2.03	1.02
Peroxynitrite isomerase THAP4	Q8WY91	THAP4	1.14 × 10^–5^	0.001615	5.12	2.36
Myeloid leukemia factor 2	Q15773	MLF2	1.24 × 10^–5^	0.001677	2.87	1.52
Alpha-2-antiplasmin	P08697	SERPINF2	1.39 × 10^–5^	0.001739	1.45	0.53
Plasma kallikrein	P03952	KLKB1	1.51 × 10^–5^	0.001801	1.62	0.70
Hemopexin	P02790	HPX	1.57 × 10^–5^	0.001863	1.77	0.83

B–H = Benjamini–Hochberg correction critical value

Serum GMDS, cation channel sperm-associated protein 2, torsin-1A, endoplasmin, and DNA-directed RNA polymerases I and III subunit RPAC1 showed the highest VIP scores for post-surgical KOA compared to control (Figure 4B). In the PLS-DA, components 1–2 clearly separated the control and post-surgical KOA samples (Figure 4C). The ROC analysis emphasized UDP-N-acetylglucosamine--peptide N-acetylglucosaminyltransferase 110 kDa subunit, lysine-specific demethylase PHF2, protein AMBP, coagulation factor XI, CFI, heparin cofactor 2, complement component C6, CAPN3, A-kinase anchor protein 6, and glucosylceramide transporter ABCA12 as the top 10 proteins with the greatest biomarker potential for post-surgical KOA vs. control, with an AUC of 1.000 and both sensitivity and specificity of 1.000.

Post-surgical KOA-related GO biological processes included complement activation, complement activation (classical pathway), immunoglobulin-mediated immune response, humoral immune response, and several processes related to coagulation, inflammatory response, immune response, wound healing, and lipoprotein, cholesterol, and phospholipid metabolism, among other processes (Figure 4D). GO molecular functions involved, e.g., endopeptidase inhibitor activity, peptidase regulator activity, serine-type endopeptidase inhibitor activity, glycosaminoglycan binding, serine-type endopeptidase activity, and functions related to complement binding, heparin binding, cholesterol/lipid transfer, and antioxidant activity, and the KEGG pathways contained complement and coagulation cascades, systemic lupus erythematosus, cholesterol metabolism, and vitamin digestion and absorption. In the PPI network, the number of nodes was 238, the number of edges was 1410, the average node degree was 11.8, the average local clustering coefficient was 0.441, and the PPI enrichment p-value was <1.0 × 10^-16^ (Figure 4E). The top hub proteins in the post-TKA PPI network included albumin, beta-2-glycoprotein 1, VTN, APOA1, APOE, and plasminogen with central roles in lipid metabolism, coagulation and fibrinolysis, ECM interactions, and the regulation of inflammatory processes. Together, our findings suggest that, despite surgical intervention, KOA patients would have sustained low-grade inflammation, altered coagulation pathway, and metabolic dysregulation.

### Associations of Proteomics With Knee Pain and Function

**Figure 5.**
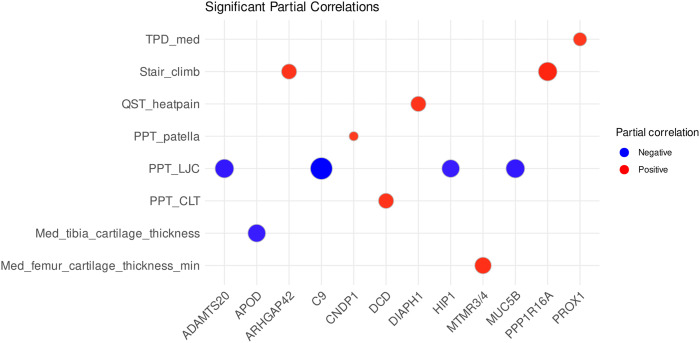
Significant Pearson correlations between serum proteins and clinical variables adjusted for sex, age, and body mass index in controls and patients with baseline knee osteoarthritis. The dot size increases with correlation magnitude: the largest dot (C9 vs. PPT_LJC) has the correlation coefficient of –0.9358003 and the smallest (CNDP1 vs. PPT_patella) 0.8495099. Proteins are labeled using their gene symbols, the full names of the proteins and their UniProt accession numbers are listed in Supplemental Table S1. TPD = two-point discrimination, med = medial, QST = quantitative sensory testing, PPT = pressure pain threshold, LJC = lateral joint capsule, CLT = lateral tibial condyle, min = minimum

Numerous significant associations (FDR <0.05) were identified with the Pearson correlation analysis between the detected proteins, cartilage thickness, and measures of pain, physical performance, and sensorimotor function, after adjusting for sex, age, and BMI. However, when data from all three study groups (control, baseline KOA, post-surgical KOA) were analyzed together, no correlations reached significance at FDR <0.05. In the analysis including the controls and baseline KOA patients, 12 significant correlations were identified (Figure 5). These included, for instance, associations of complement component C9 (C9), a disintegrin and metalloproteinase with thrombospondin motifs 20 (ADAMTS20), and Beta-Ala-His dipeptidase with pain sensitivity, protein phosphatase 1 regulatory subunit 16A (PPP1R16A) and Rho GTPase-activating protein 42 (ARHGAP42) with stair climb performance, and APOD and phosphatidylinositol-3,5-bisphosphate 3-phosphatase MTMR3/4 with articular cartilage thickness. A comprehensive list of the significant results is presented in Supplemental Table S2.

**Figure 6.**
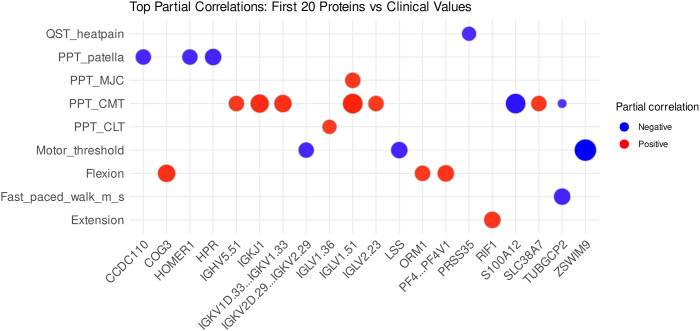
Top 20 significant Pearson correlations between serum proteins and clinical variables adjusted for sex, age, and body mass index in patients with baseline knee osteoarthritis (KOA) and post-surgical KOA. The dot size increases with correlation magnitude: the largest dot (ZSWIM9 vs. Motor_threshold) has the correlation coefficient of –0.9378151 and the smallest (TUBGCP2 vs. PPT_CMT) –0.8261381. Proteins are labeled using their gene symbols, the full names of the proteins and their UniProt accession numbers are listed in Supplemental Table S1. QST = quantitative sensory testing, PPT = pressure pain threshold, MJC = medial joint capsule, CMT = medial tibial condyle, CLT = lateral tibial condyle

The analysis of the baseline and post-surgical KOA patients revealed 56 significant associations when adjusted for sex, age, and BMI. Several proteins, such as peroxisome proliferator-activated receptor gamma (PPARG), S100A12, C8A, fibrinogen alpha chain (FGA), transthyretin, and many immunoglobulin variable region proteins, were related to objective pain sensitivity (Figure 6, Supplemental Table S3).

There were also several significant correlations between particular proteins and ROM of the knee joint (e.g., alpha-1-acid glycoprotein 1 and 2, platelet factor 4), physical performance (e.g., contactin-associated protein-like 4, PPP1R16A), rMT (e.g., muscarinic acetylcholine receptor M3 [CHRM3], lanosterol synthase [LSS], immunoglobulin kappa variable 3D-15), and threshold for heat pain (e.g., cytochrome P450 7A1, mannan-binding lectin serine protease 1, phosphatidylinositol 4,5-bisphosphate 3-kinase catalytic subunit alpha isoform). For a full overview of the significant results, see Supplemental Table S3.

## Discussion

The present study on KOA investigated longitudinal changes in the serum proteome and their associations with subjective and objective measures of pain, physical performance, sensation, and neuromuscular function before and after TKA. The findings demonstrate the potential of serum proteins as biomarkers to distinguish individuals with KOA from controls, even post-surgery. KOA associated with alterations in key biological pathways, and several proteins emerged as predictors of pain sensitivity, physical limitations, and sensorimotor function. In summary: (*i*) baseline KOA shows enrichment of proteins involved in complement activation, immune response, coagulation, inflammatory response, calcium homeostasis, ECM remodeling, and oxidative stress, (*ii*) post-TKA proteomic alterations can reflect both tissue repair and residual pathophysiology, and (*iii*) several serum proteins mirror KOA pain and disability, with potential as biomarkers or therapeutic targets.

### Baseline KOA Is Characterized by Systemic Proteomic Alterations

The clear separation between control and KOA baseline samples underscores the diagnostic potential of serum proteins and points to a broader systemic involvement beyond the joint tissues alone. The KOA-associated DEPs were linked to the complement and coagulation cascades that, based on previous SF and serum proteomic studies, may have crosstalk in OA joints and play roles in disease progression.^19,20^ In the present study, baseline KOA-related proteins involved in coagulation cascades included, for instance, coagulation factors IX and XI, prothrombin, plasminogen, and SERPINF2. Complement can be activated by various ECM components and their cleavage products released during cartilage degradation in OA.^21^ As the complement and coagulation systems are interconnected and capable of activating one another,^22^ it is possible that their interactions could amplify inflammatory responses^23^ and contribute to further joint damage. CFI, in particular, stood out as a potential biomarker warranting further validation in larger experimental or clinical settings.

Alterations in calcium homeostasis were indicated by increased levels of S100A16, ryanodine receptor 3, calcium homeostasis modulator protein 3, and alpha-2-HS-glycoprotein in baseline KOA. These observations align with current understanding of OA pathophysiology as, in addition to cartilage degeneration, OA also features abnormal calcification in joint tissues, such as articular cartilage,^24^ calcified cartilage,^25^ and menisci.^26^ Several proteins involved in calcium regulation, including S100A6^4^ and osteocalcin,^27^ have previously been reported to be dysregulated in OA. Notably, S100A7 was identified among the top 10 hub proteins in the PPI network analysis of OA calcified cartilage,^28^ and it is known to possess proinflammatory properties.^29^ Moreover, particular proteins previously shown to be altered in calcified cartilage from patients with advanced KOA^28^ were also dysregulated in the serum of end-stage KOA patients before or after TKA (fibronectin, APOA1, ECM protein 1 [ECM1]). Collectively, these findings suggest a multifaceted disruption of calcium-related pathways in OA pathogenesis that could contribute to inflammation, chondrocyte dysfunction, ECM degradation, and pathologic calcification.

Extensive literature demonstrates that OA involves profound remodeling of the ECM.^30^ ECM-related pathways have been previously highlighted in the plasma proteomics of KOA patients,^8^ and several proteins involved in the ECM and its remodeling (e.g., fibronectin, ITIH2, plasminogen, SERPINF2, VTN, and fibulin-1) were also documented to change in the present study. Fibronectin was previously identified as one of the top 10 hub proteins in the PPI network analysis of KOA calcified cartilage,^28^ further underscoring its central role in OA. These findings support the notion that ECM remodeling is a fundamental process in OA,^30^ and the observed proteomic alterations presumably reflect the ongoing cartilage breakdown and attempted repair processes. Szilagyi et al^7^ reported that plasma levels of the ECM protein CRTAC1 were associated with OA severity and progression. However, we did not find significant correlations with cartilage thickness or differences in its abundance between the study groups.

### Comparative Serum Proteomics of Baseline and Post-surgical KOA

The systemic proteomic alterations that emerged following TKA may indicate residual pathophysiological processes while also representing potential biomarkers for evaluating surgical recovery or ongoing joint dysfunction. The increased abundance of complement-related proteins and coagulation factor XI, plasma kallikrein, and heparin cofactor 2 after surgery supports the concept of coagulation–inflammation crosstalk^31^ that could relate to persistent systemic low-grade inflammation after TKA.^32^ Occasionally, synovial tissue remains in joints following TKA and may be associated with residual inflammation.^33^ Additionally, operated patients can experience effusion and Hoffa’s fat pad synovitis.^34^ Regarding the patients of the present study, residual synovitis would be unlikely, and the C-reactive protein abundances were not elevated in baseline KOA or after TKA.

Instead, the differences in proteomics could relate to extra-articular tissue inflammation during healing and scarring at the surgical site itself, including bone cuts and large wounds in soft tissues. The new anatomical situation after TKA involves inflammation–resolution processes, along with a general stress response triggered by the surgery and anesthesia. During rehabilitation, stretching of healing tissues, microtrauma, and reactions to the joint implant may also influence systemic proteomic pathways. Proteins, such as ITIH2, ECM1, and FERMT2, may contribute to ECM remodeling or fibrotic processes,^35-37^ which are involved in tissue repair but may also promote joint stiffness and functional limitations. The upregulation of DNMT3A and DNMT3L in post-surgical (and baseline) KOA suggests epigenetic changes in response to joint injury, inflammation, or surgery.^38^ Increased levels of vitamin D-binding protein and glutathione peroxidase 3 could indicate alterations in calcium metabolism and oxidative stress responses. Overall, the systemic proteomic alterations observed after TKA likely reflect a multifactorial response involving inflammation–resolution dynamics, tissue remodeling, metabolic adaptations, and surgical stress, rather than isolated residual joint pathology.

### Persistent Systemic Proteomic Alterations in KOA Patients One Year After TKA

Twelve months post-TKA, the serum proteome clearly differed between patients and healthy individuals. The proteomic profile of the recovering patients was characterized by persistent alterations in the complement and coagulation cascades, immune response, and ECM remodeling pathways, and dysregulation of oxidative stress responses and lipid metabolism. These findings may reflect incomplete resolution of disease-related processes or long-term effects of surgical intervention. It is also common for KOA patients to have OA in the contralateral knee^13,14^ or in other joints, which could contribute to the persistently abnormal serum proteomic profiles post-TKA.

Several complement-related proteins remained elevated 12 months after TKA and could reflect unresolved systemic low-grade inflammation^32^ or ongoing tissue remodeling. Complement activation is increasingly recognized in the pathogenesis of OA,^39^ and the higher abundance of coagulation factor XI, heparin cofactor 2, SERPINF2, and plasma kallikrein could imply interactions between coagulation and inflammation.^31^ This aligns with growing evidence that coagulation pathways contribute to OA pathology^40,41^ and, based on the current results, they seem to remain active post-TKA. Upregulation of glutathione peroxidase 3 can reflect a systemic oxidative stress response, which may be also tied to inflammation or post-TKA tissue recovery. The downregulated CAPN3 separated post-surgical KOA from both controls and baseline KOA. It is a muscle-specific calcium-dependent cysteine protease essential for muscle formation and remodeling,^42^ with an unknown role in post-surgical KOA. As muscle dysfunction, particularly quadriceps weakness, is a hallmark of KOA, and preoperative quadriceps strength is a strong predictor of post-TKA functional performance,^43^ the downregulation of CAPN3 might affect muscle recovery after surgery and contribute to altered joint mechanics or functional limitations.

Even though several studies have measured biochemical markers (e.g., clinical chemistry and hematology) in circulation during the early post-operative phase after TKA,^44^ research comparing blood biomarkers before TKA and after long-term recovery is limited. We previously documented minor changes in plasma fatty acids^45^ and amino acids^46^ measured 3 and/or 12 months after TKA in the same patient cohort. Following TKA, the levels of lignoceric acid (24:0), nervonic acid (24:1n-9), and the dipeptide cystine were altered, with pathway analysis suggesting that taurine and hypotaurine metabolism could be affected by surgery.^45,46^ Styrkarsdottir et al^5^ also noted a difference in levels of OA-associated proteins between patients who had undergone joint replacement before plasma collection and those who had not. The protein with the strongest association with prior joint replacement, CUB domain-containing protein 1 (CDCP1), was not associated with OA without joint replacement. CDCP1 was not affected by KOA or TKA in the current dataset. In Styrkarsdottir et al,^5^ CDCP1 levels changed very little during the 20 years preceding joint replacement, followed by an increase in the levels at the time of arthroplasty, and a continued steady rise thereafter. Together, these findings suggest that the recovery from KOA is slow, and TKA does not fully normalize systemic biochemical processes within 12 months. This supports the growing view of OA as a whole-body condition characterized by molecular dysregulation beyond the joint^47^ that seems to be persistent and not fully reversible by TKA.

### Potential KOA Biomarkers in Serum

Previous studies have identified potential serum and plasma biomarkers associated with OA severity and progression, including CRTAC1, COMP, THBS4, IL18R1, fibrillin-1, vitamin D-binding protein, clusterin, lubricin, complement C3, ITIH1, and S100A6.^3-7^ However, most of these findings could not be replicated in our dataset. Some of the molecules were not detected, while no significant differences were observed in CRTAC1, clusterin, or vitamin D-binding protein in baseline KOA. However, ITIH2–4 and several complement-related proteins, including complement C3, showed increased abundance. Additionally, S100A12 and S100A16 were upregulated, whereas S100A6 was not detected. These discrepancies may reflect differences in sample types, disease stages, or analytical approaches and highlight the need for further validation of the proposed biomarkers in larger and more diverse cohorts. Other proteins found to be elevated in baseline KOA—many also previously associated with OA through altered levels—included, e.g., VTN, HABP2, fetuin-B, hemopexin, haptoglobin, serum amyloid P-component, and pigment epithelium-derived factor.^6,48,49^

### Associations of Proteomics With Knee Function, Sensation, and Pain

Previous research indicates that circulating proteins related to, e.g., inflammation, ECM remodeling, and oxidative stress can be involved in pain perception and physical function in KOA. For instance, plasma CRTAC1 concentrations have been associated with joint pain,^5^ and Giordano et al^11^ identified IL6, macrophage colony-stimulating factor 1, fibroblast growth factor-21, and TNF superfamily member 12 as key serum markers associated with subjective KOA pain intensity. In Naili et al,^8^ plasma ATPase 13, hemoglobin subunit delta, and peroxiredoxin-2 were linked to KOA pain, and many other proteins with multifunctional roles were related to performance-based joint function and kinematic measures. Additionally, elevated serum IL8 levels were associated with worse knee function 5 years after TKA.^50^

In the present study, a total of 12 significant associations emerged in the analysis of controls and baseline KOA patients adjusted for sex, age, and BMI, while a broader set of 56 significant correlations was observed for baseline and post-surgical KOA. Elevated levels of C9, ADAMTS20, S100A12, and FGA were linked to increased pain sensitivity (lower PPT), whereas several immunoglobulin variable region proteins were associated with reduced sensitivity. Together with prior evidence,^51^ these findings support a role for circulating antibodies or their components in pain modulation, potentially via anti-inflammatory mechanisms. Some of the other proteins mentioned above, such as C9 and S100A12, could promote KOA pain through immune/inflammatory or tissue-remodeling pathways.^52,53^ Collectively, these results suggest that circulating immune-related proteins could affect pain modulation and may have biomarker or therapeutic relevance in KOA.

Impaired physical performance was associated with ARHGAP42, PPP1R16A, and gamma-tubulin complex component 2, suggesting that alterations in vascular tone, myosin-driven contractility, and microtubule organization^54-56^ may be related to impaired mobility in KOA. These proteins could serve as indirect markers of functional capacity and provide insight into the molecular basis of KOA-related physical decline. rMT, a neurophysiological measure of corticospinal excitability, was positively associated with CHRM3 and inversely associated with LSS. CHRM3, the muscarinic acetylcholine receptor M3, may indicate altered cholinergic signaling in the central nervous system,^57^ while LSS, a key enzyme in cholesterol biosynthesis, could signal disruptions in lipid metabolism affecting synaptogenesis and neurotransmission.^58^ These associations suggest cholinergic and lipid-related mechanisms in modulating central motor control in KOA. Additionally, higher levels of APOD were associated with thinner tibial cartilage, possibly reflecting oxidative stress or ongoing inflammation.^59^

Together, these findings suggest that specific circulating proteins reflect the complex interplay between immune, neural, and structural pathways underlying clinical KOA manifestations. In addition, this indicates that KOA progression could be assessed from blood samples in addition to radiological evaluation, with potential prognostic value. Obviously, further validation in independent cohorts is needed to confirm the clinical relevance of the results and to discover the proteins that would be the most useful candidates as biomarkers or therapeutic targets. A limitation of this study to be acknowledged is the relatively small number of samples, which leads to reduced statistical power, increases the risk of type II errors, and limits the generalizability of the identified biomarkers and their associations with pain and functional outcomes. Additionally, the differences in average age and body adiposity between the study groups represent important limitations. Although these factors were accounted for in our statistical analyses, they should still be considered when interpreting the results. It should also be acknowledged that generalized inflammatory processes, such as complement and immune-related pathways, are not specific to KOA and may overlap with other conditions, such as infection. This highlights the value of other markers, e.g., those linked to ECM remodeling, which would be more directly reflective of joint-specific pathology.

## Supplemental Material

Supplemental material - Proteomics Analysis Reveals Serum Biomarkers Reflecting Joint Pain and Physical Limitations in Knee Osteoarthritis Before and After Joint Replacement Surgery Supplemental material for Proteomics Analysis Reveals Serum Biomarkers Reflecting Joint Pain and Physical Limitations in Knee Osteoarthritis Before and After Joint Replacement Surgery by Anne-Mari Mustonen, Janne Tampio, Kristiina M. Huttunen, Petro Julkunen, Laura Säisänen, Lauri Karttunen, Jusa Reijonen, Amir Esrafilian, Tiina Kuningas, Atul Kumar, Heikki Kröger and Petteri Nieminen in Cartilage.

Supplemental material - Proteomics Analysis Reveals Serum Biomarkers Reflecting Joint Pain and Physical Limitations in Knee Osteoarthritis Before and After Joint Replacement Surgery Supplemental material for Proteomics Analysis Reveals Serum Biomarkers Reflecting Joint Pain and Physical Limitations in Knee Osteoarthritis Before and After Joint Replacement Surgery by Anne-Mari Mustonen, Janne Tampio, Kristiina M. Huttunen, Petro Julkunen, Laura Säisänen, Lauri Karttunen, Jusa Reijonen, Amir Esrafilian, Tiina Kuningas, Atul Kumar, Heikki Kröger and Petteri Nieminen in Cartilage.

Supplemental material - Proteomics Analysis Reveals Serum Biomarkers Reflecting Joint Pain and Physical Limitations in Knee Osteoarthritis Before and After Joint Replacement Surgery Supplemental material for Proteomics Analysis Reveals Serum Biomarkers Reflecting Joint Pain and Physical Limitations in Knee Osteoarthritis Before and After Joint Replacement Surgery by Anne-Mari Mustonen, Janne Tampio, Kristiina M. Huttunen, Petro Julkunen, Laura Säisänen, Lauri Karttunen, Jusa Reijonen, Amir Esrafilian, Tiina Kuningas, Atul Kumar, Heikki Kröger and Petteri Nieminen in Cartilage.

Supplemental material - Proteomics Analysis Reveals Serum Biomarkers Reflecting Joint Pain and Physical Limitations in Knee Osteoarthritis Before and After Joint Replacement Surgery Supplemental material for Proteomics Analysis Reveals Serum Biomarkers Reflecting Joint Pain and Physical Limitations in Knee Osteoarthritis Before and After Joint Replacement Surgery by Anne-Mari Mustonen, Janne Tampio, Kristiina M. Huttunen, Petro Julkunen, Laura Säisänen, Lauri Karttunen, Jusa Reijonen, Amir Esrafilian, Tiina Kuningas, Atul Kumar, Heikki Kröger and Petteri Nieminen in Cartilage.

Supplemental material - Proteomics Analysis Reveals Serum Biomarkers Reflecting Joint Pain and Physical Limitations in Knee Osteoarthritis Before and After Joint Replacement Surgery Supplemental material for Proteomics Analysis Reveals Serum Biomarkers Reflecting Joint Pain and Physical Limitations in Knee Osteoarthritis Before and After Joint Replacement Surgery by Anne-Mari Mustonen, Janne Tampio, Kristiina M. Huttunen, Petro Julkunen, Laura Säisänen, Lauri Karttunen, Jusa Reijonen, Amir Esrafilian, Tiina Kuningas, Atul Kumar, Heikki Kröger and Petteri Nieminen in Cartilage.

Supplemental material - Proteomics Analysis Reveals Serum Biomarkers Reflecting Joint Pain and Physical Limitations in Knee Osteoarthritis Before and After Joint Replacement Surgery Supplemental material for Proteomics Analysis Reveals Serum Biomarkers Reflecting Joint Pain and Physical Limitations in Knee Osteoarthritis Before and After Joint Replacement Surgery by Anne-Mari Mustonen, Janne Tampio, Kristiina M. Huttunen, Petro Julkunen, Laura Säisänen, Lauri Karttunen, Jusa Reijonen, Amir Esrafilian, Tiina Kuningas, Atul Kumar, Heikki Kröger and Petteri Nieminen in Cartilage.

## Data Availability

All relevant data analyzed during this study are included in this published article and its supplementary information files.*

## Appendix

### List of Abbreviations

ADAMTS20: A disintegrin and metalloproteinase with thrombospondin motifs 20
APOA1/D/E: Apolipoprotein A1/D/E
ARHGAP42: Rho GTPase-activating protein 42
AUC: Area under the curve
BMI: Body mass index
C9/8A: Complement component C9/C8 alpha chain
CAPN: Calpain
CDCP1: CUB domain-containing protein 1
CFI: Complement factor I
CHRM3: Muscarinic acetylcholine receptor M3
COMP: Cartilage oligomeric matrix protein
CRTAC1: Cartilage acidic protein 1
DDA: Data-dependent acquisition
DEP: Differentially expressed protein
DNMT3A/3L: DNA (cytosine-5)-methyltransferase 3A/3-like
ECM: Extracellular matrix
ECM1: Extracellular matrix protein 1
FC Fold change
FDR: False discovery rate
FERMT2: Fermitin family homolog 2
FGA: Fibrinogen alpha chain
GMDS: GDP-mannose 4,6 dehydratase
GO: Gene Ontology
HABP2: Hyaluronan-binding protein 2
IL: Interleukin
ITIH: Inter-alpha-trypsin inhibitor heavy chain
KEGG: Kyoto Encyclopedia of Genes and Genomes
KOA: Knee osteoarthritis
LC–MS: Liquid chromatography–mass spectrometry
LSS: Lanosterol synthase
MRI: Magnetic resonance imaging
nTMS: Navigated transcranial magnetic stimulation
OA: Osteoarthritis
PLS-DA: Partial least squares discriminant analysis
PPARG: Peroxisome proliferator-activated receptor gamma
PPI: Protein–protein interaction
PPP1R16A: Protein phosphatase 1 regulatory subunit 16A
PPT: Pressure pain threshold
rMT: Resting motor threshold
ROC: Receiver operating characteristic
ROM: Range of motion
RT: Room temperature
S100A6/7/12/16: S100 calcium-binding protein A6/7/12/16
SERPINF2: Alpha-2-antiplasmin
SF: Synovial fluid
STRING: Search Tool for the Retrieval of Interacting Genes/Proteins
THBS4: Thrombospondin 4
TKA: Total knee arthroplasty
TNF: Tumor necrosis factor
TPD: Two-point discrimination
VIP: Variable importance in projection
VTN: Vitronectin
