# Supplementary material for: Proteomics Analysis Reveals Serum Biomarkers Reflecting Joint Pain and Physical Limitations in Knee Osteoarthritis Before and After Joint Replacement Surgery
Source: Cartilage. 2026 May 30:19476035261455413. Online ahead of print. doi: 10.1177/19476035261455413 (PMC13222223; doi:10.1177/19476035261455413)
Supplement: Supplemental material - Proteomics Analysis Reveals Serum Biomarkers Reflecting Joint Pain and Physical Limitations in Knee Osteoarthritis Before and After Joint Replacement Surgery [file sj-pdf-1-car-10.1177_19476035261455413.pdf]

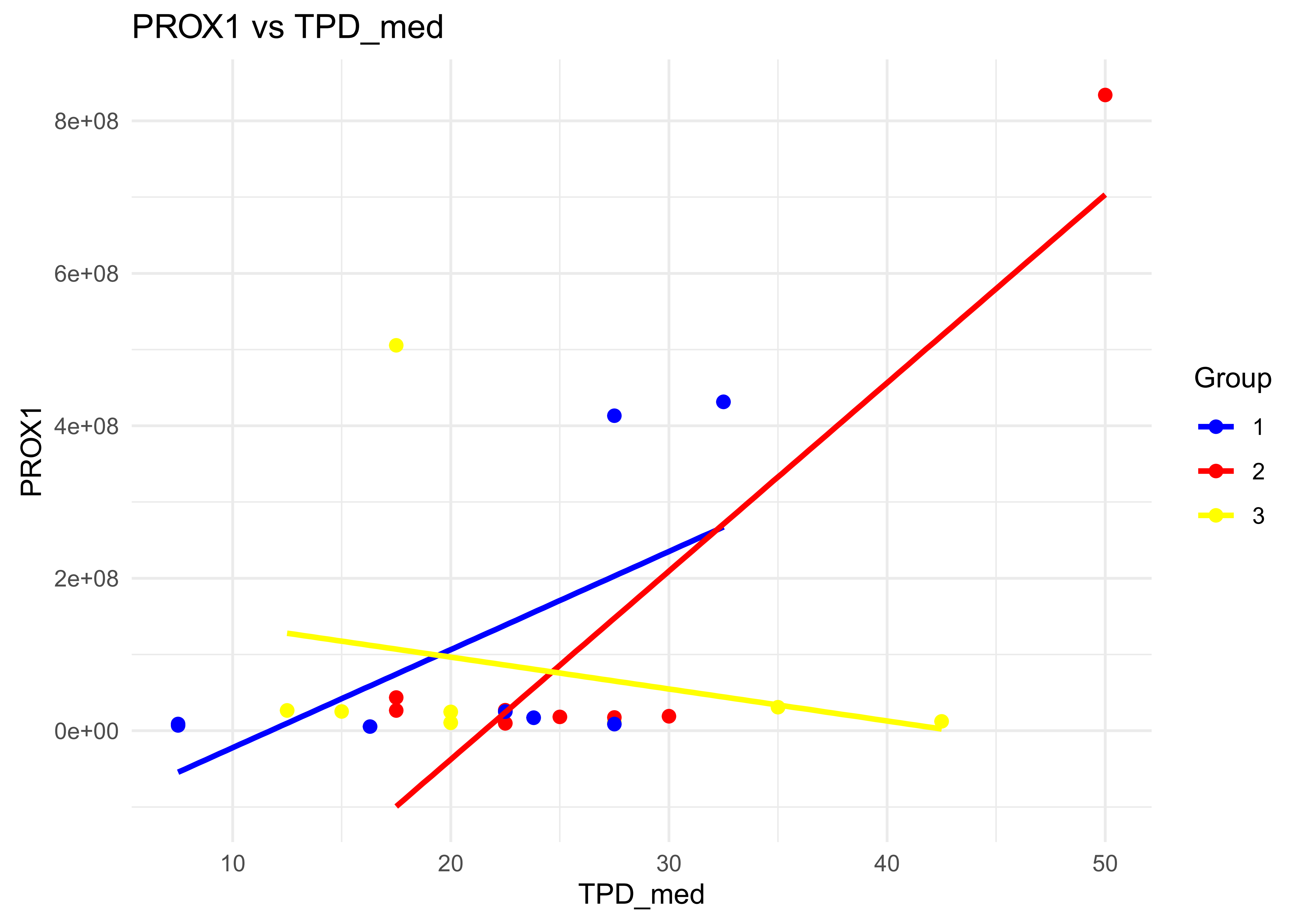

PROX1 vs TPD_med
PROX1
8e+08
6e+08
4e+08
2e+08
0e+00
10
20
30
40
50
TPD_med
Group
1
2
3

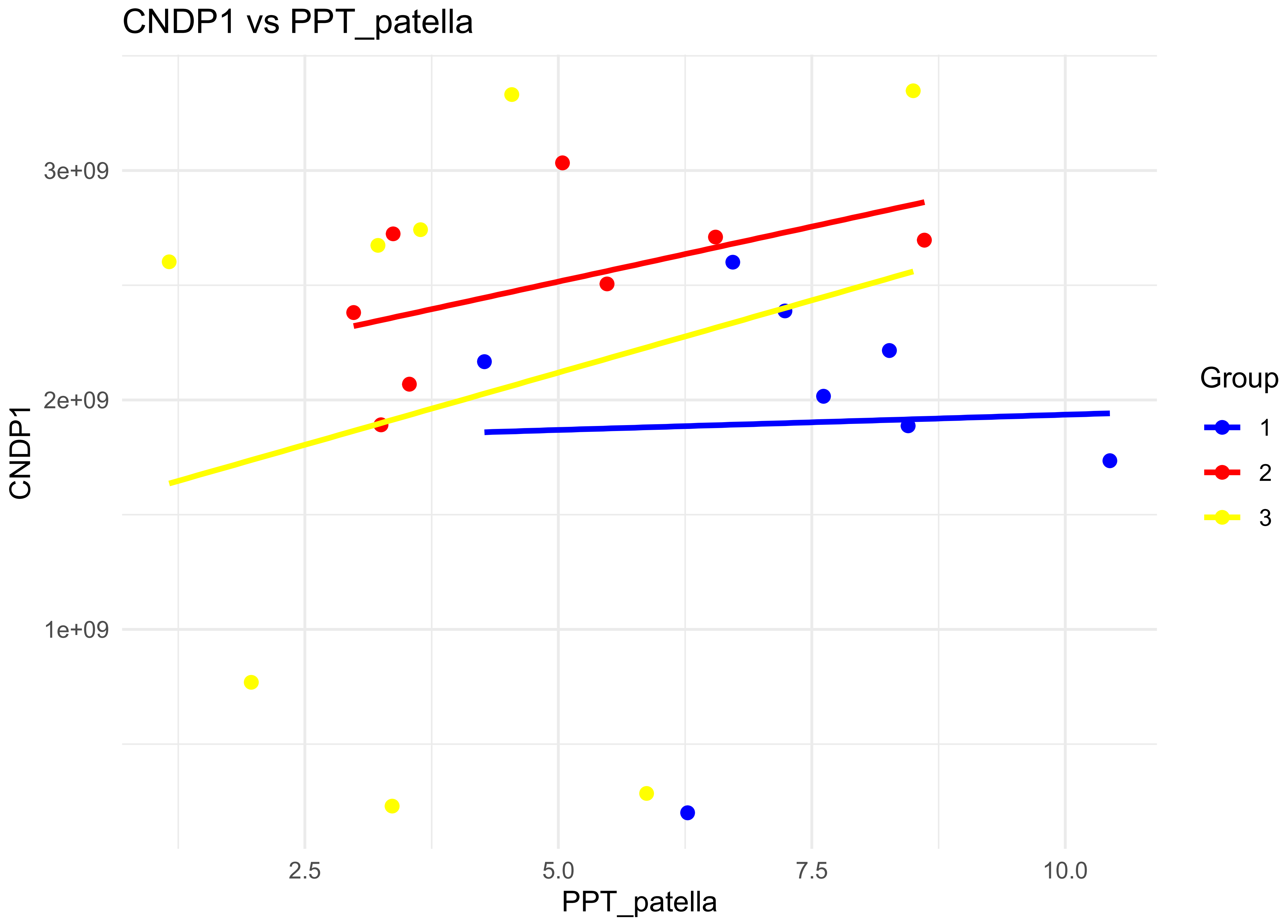
CNDP1 vs PPT_patella
CNDP1
3e+09
2e+09
1e+09
PPT_patella
2.5
5.0
7.5
10.0
Group
1
2
3

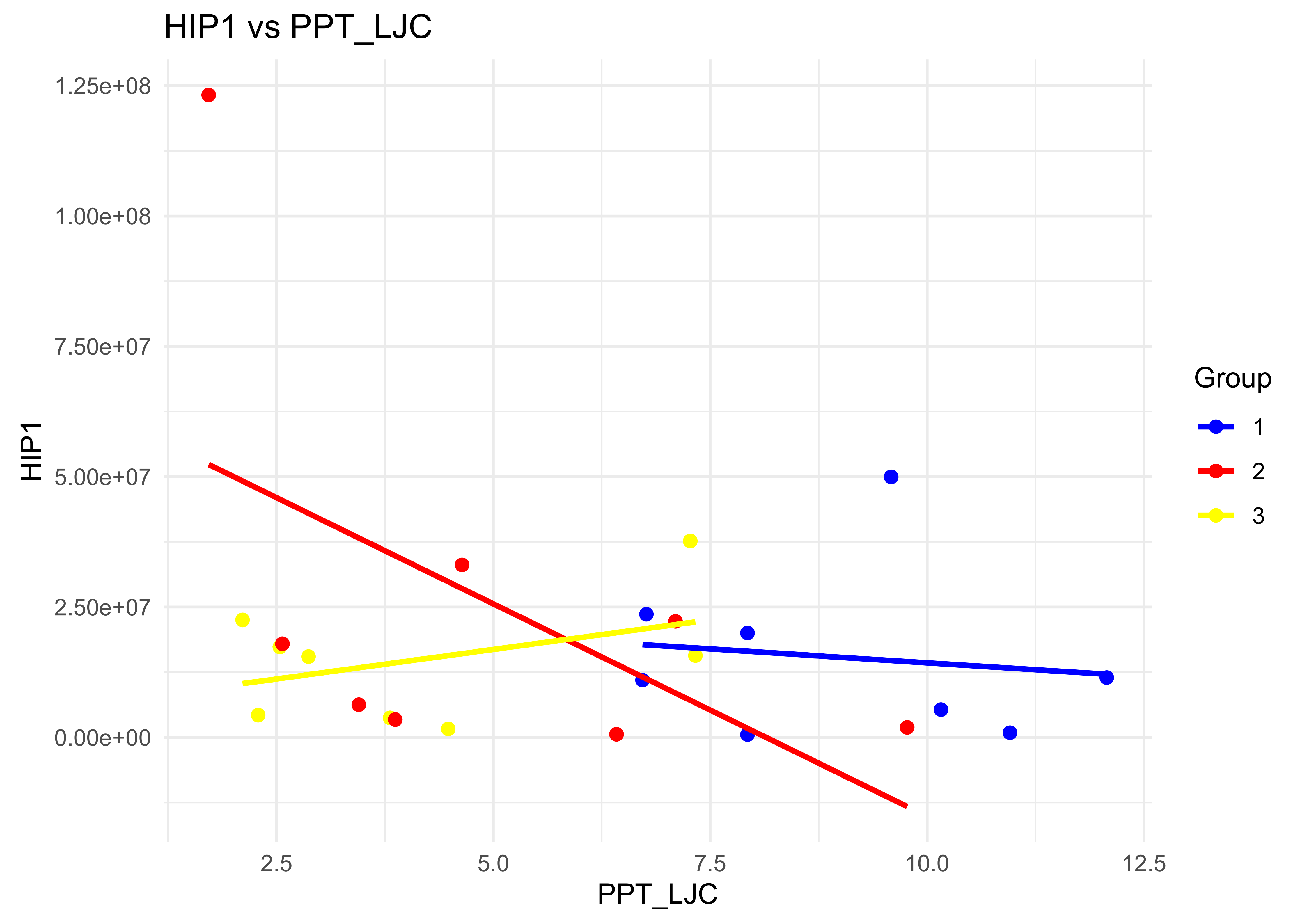

HIP1 vs PPT_LJC
1.25e+08
1.00e+08
7.50e+07
5.00e+07
2.50e+07
0.00e+00
HIP1
2.5
5.0
7.5
10.0
12.5
PPT_LJC
Group
1
2
3

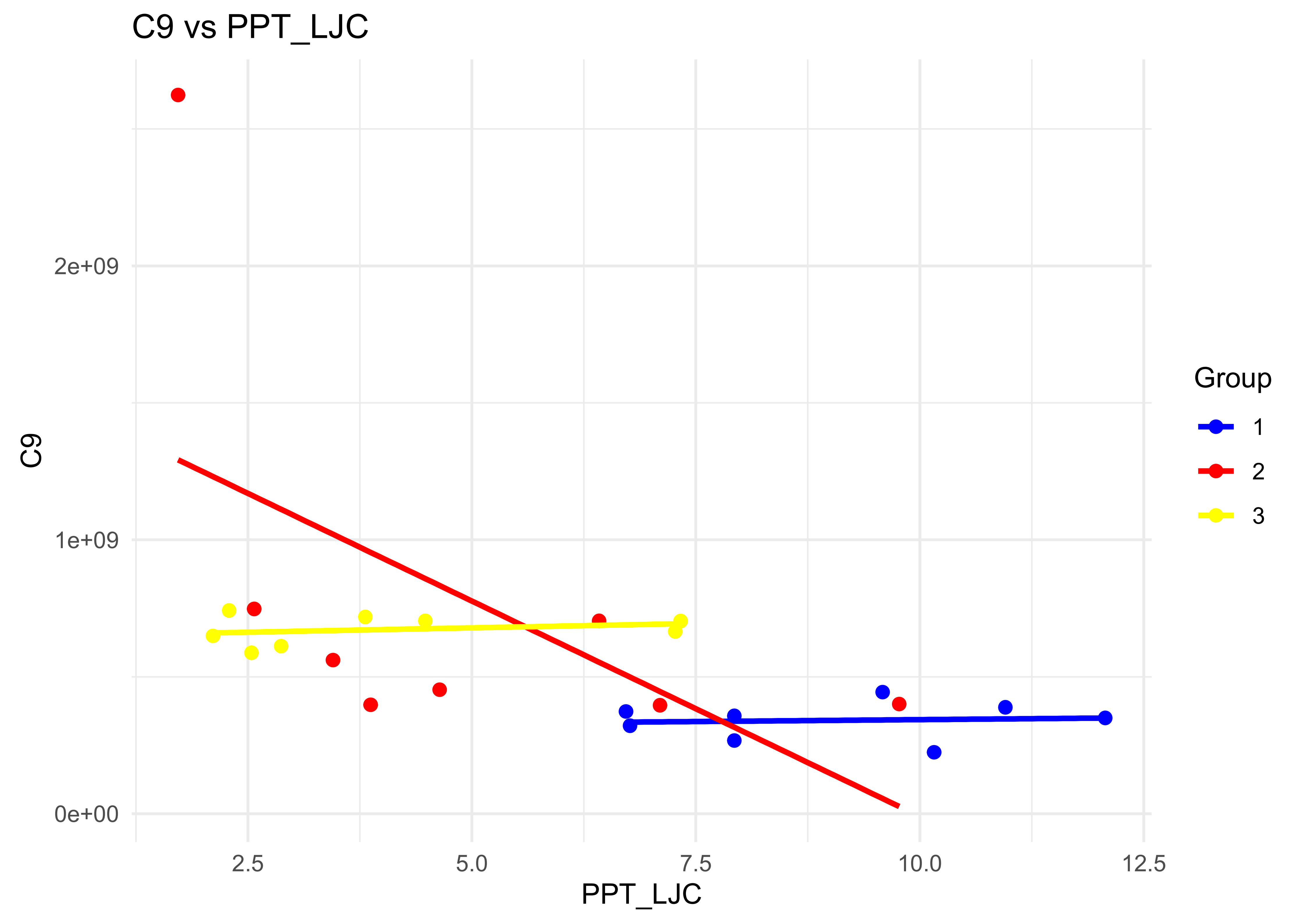

C9 vs PPT_LJC
C9
2e+09
1e+09
0e+00
2.5
5.0
7.5
10.0
12.5
PPT_LJC
Group
1
2
3

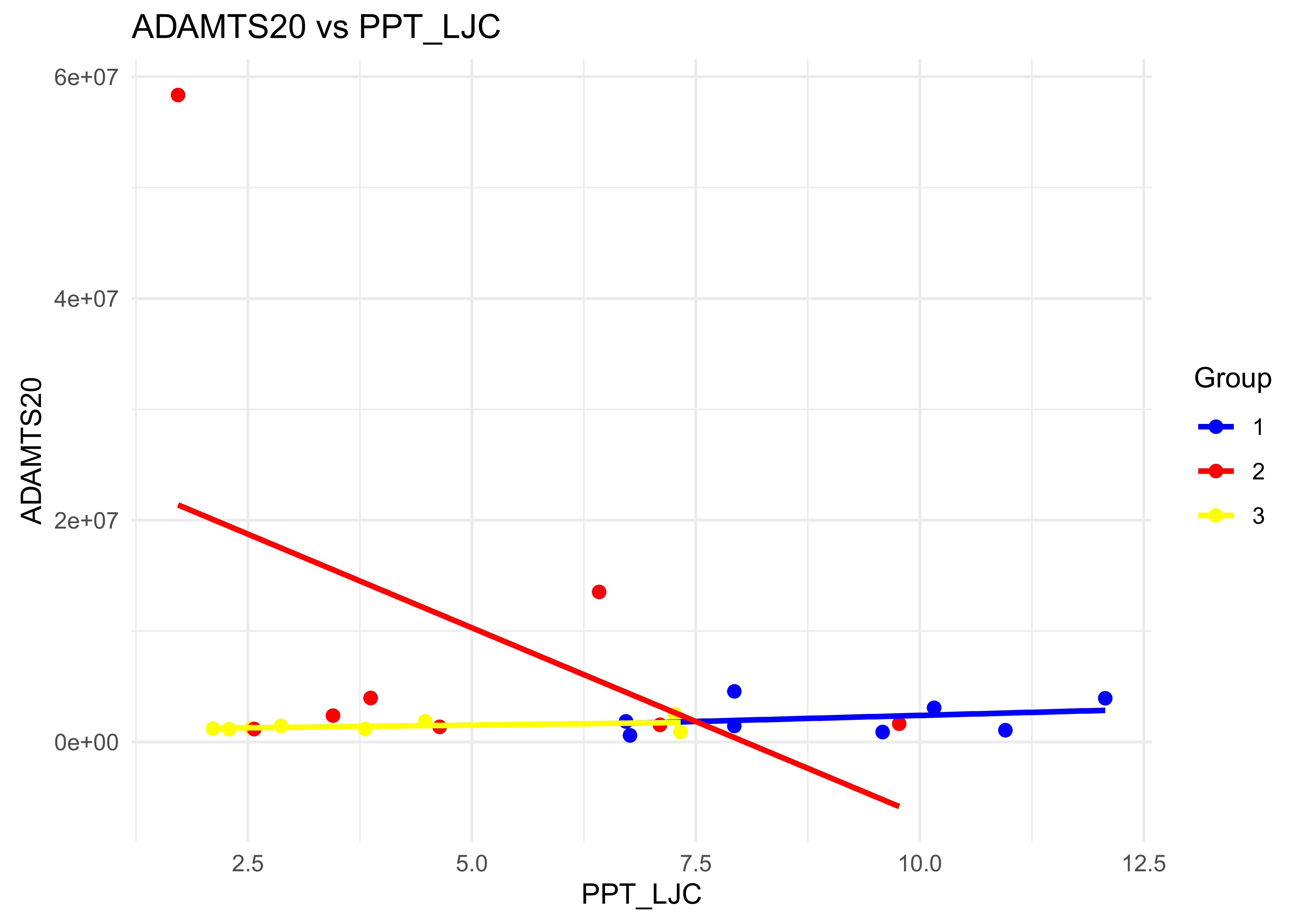
ADAMTS20 vs PPT_LJC
6e+07
4e+07
2e+07
0e+00
ADAMTS20
2.5
5.0
7.5
10.0
12.5
PPT_LJC
Group
1
2
3

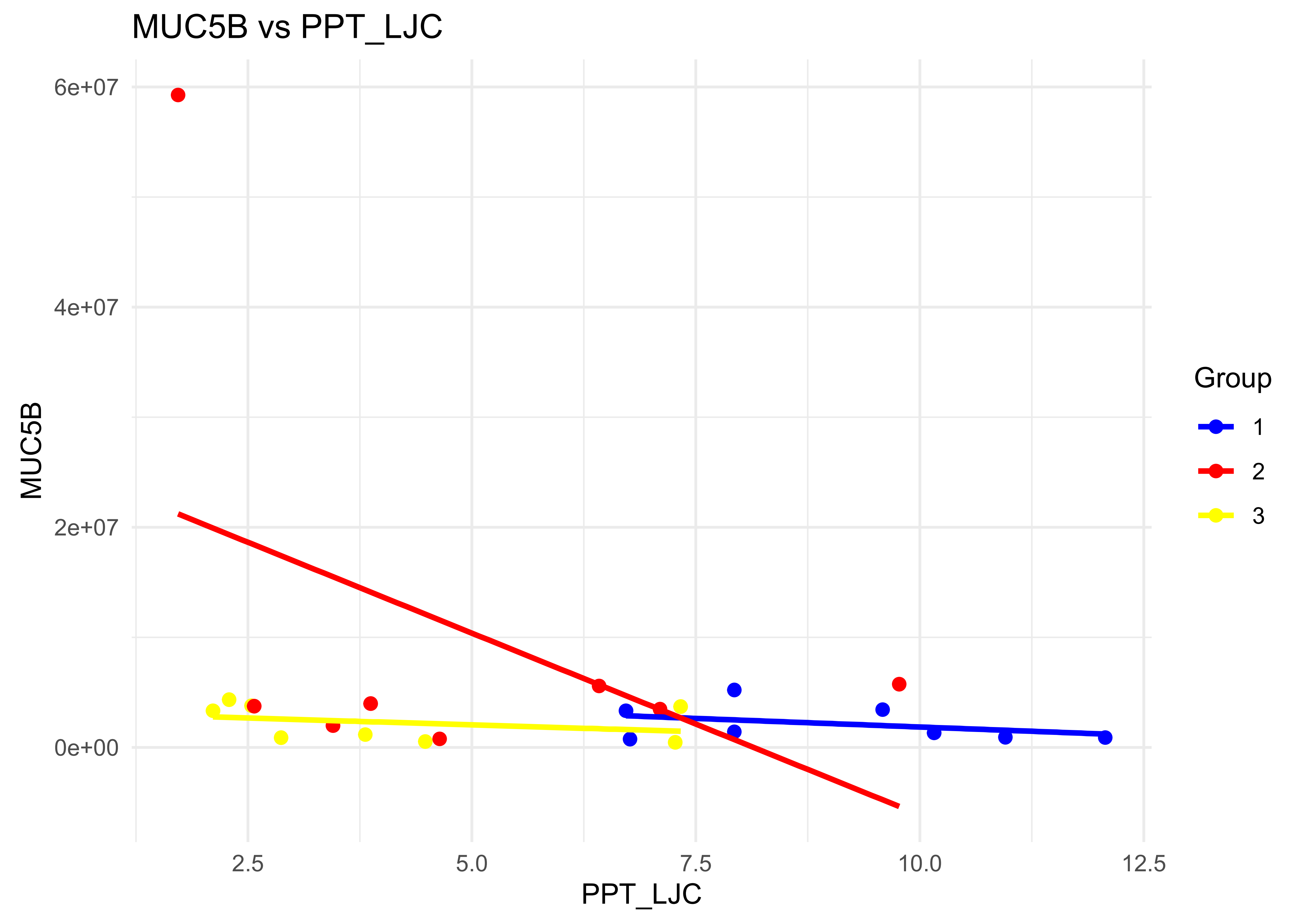

MUC5B vs PPT_LJC
6e+07
4e+07
2e+07
0e+00
MUC5B
2.5
5.0
7.5
10.0
12.5
PPT_LJC
Group
1
2
3

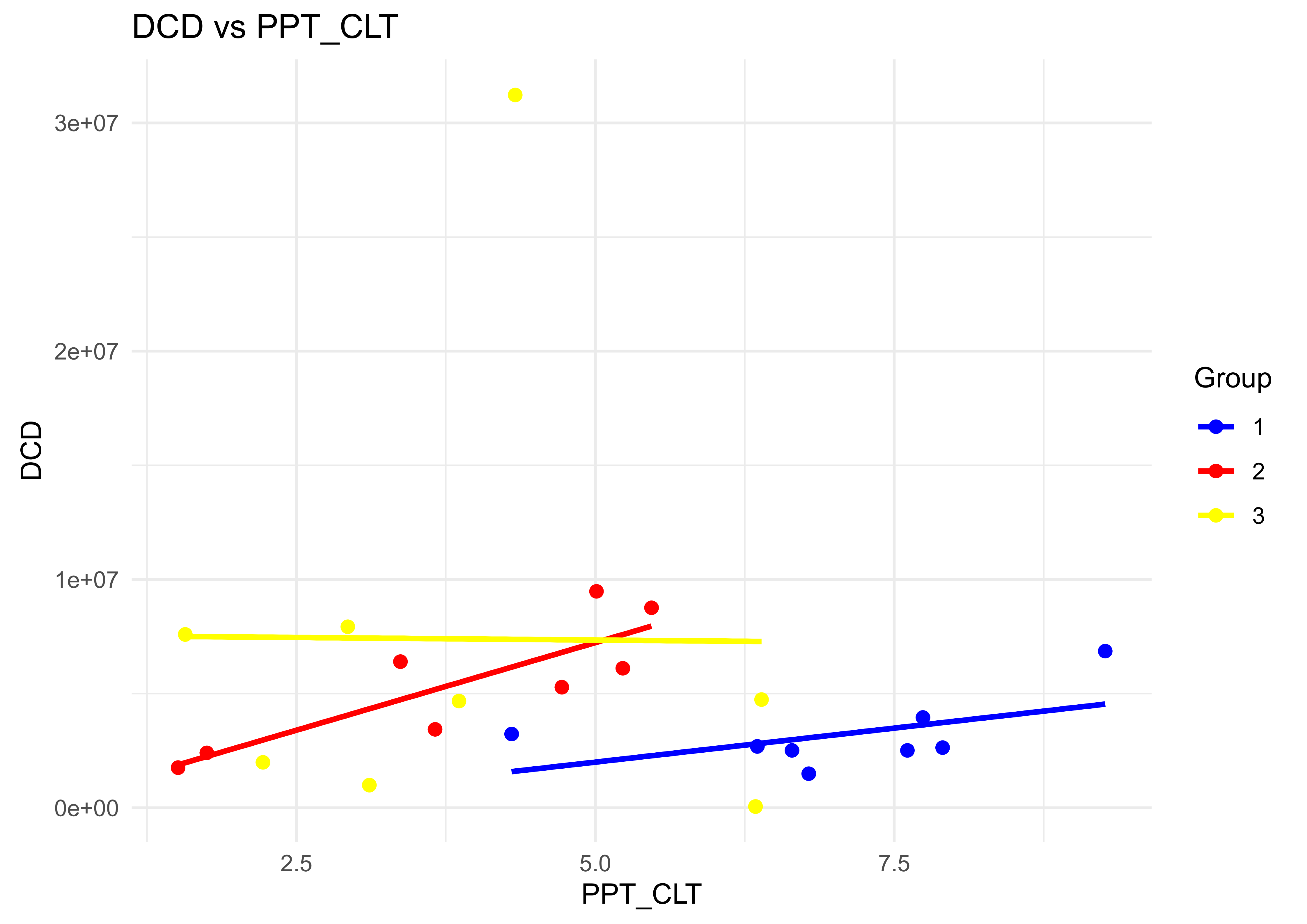

DCD vs PPT_CLT
3e+07
2e+07
1e+07
0e+00
DCD
2.5
5.0
7.5
PPT_CLT
Group
1
2
3

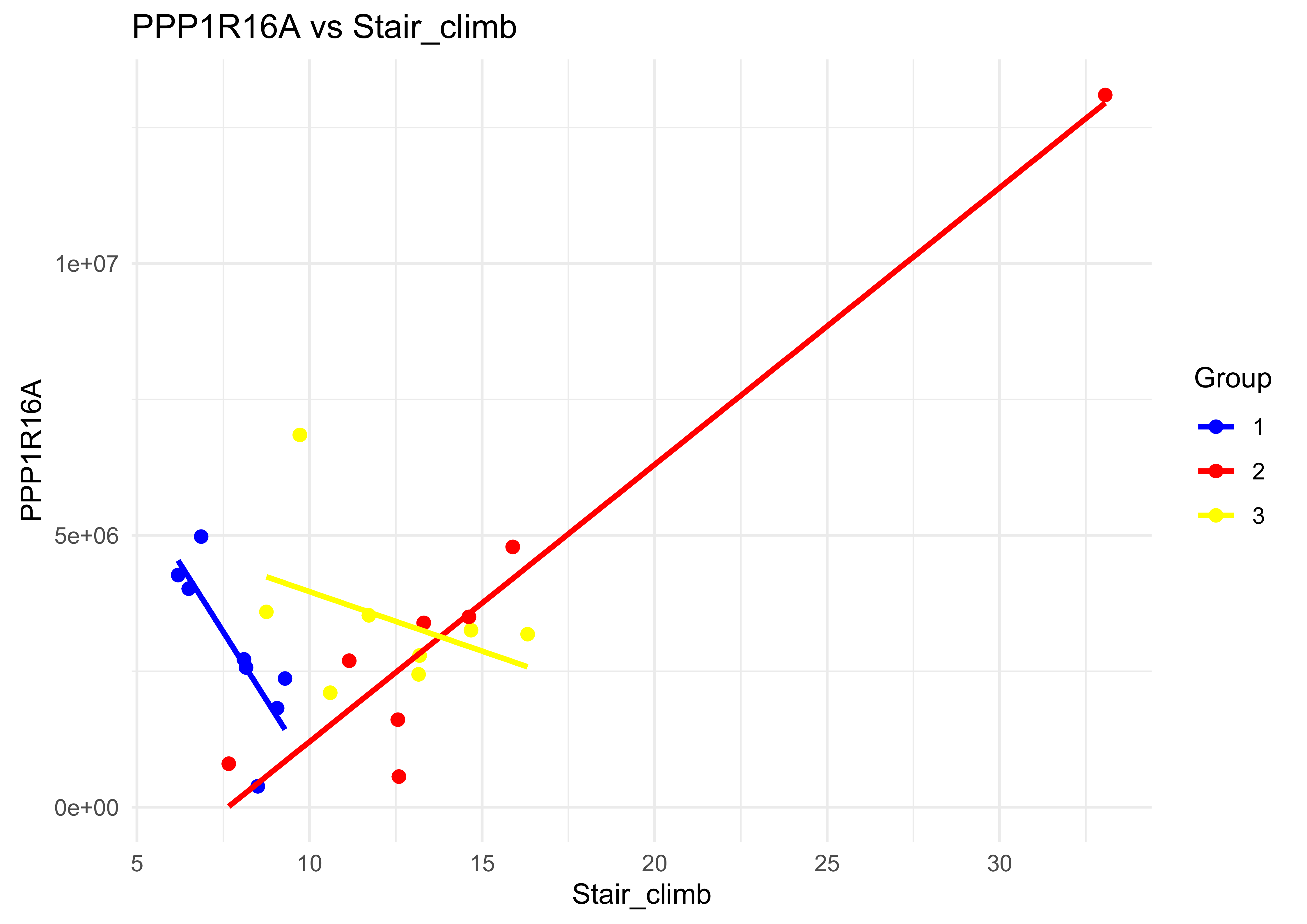
PPP1R16A vs Stair_climb
PPP1R16A
1e+07
5e+06
0e+00
5
10
15
20
25
30
Stair_climb
Group
1
2
3

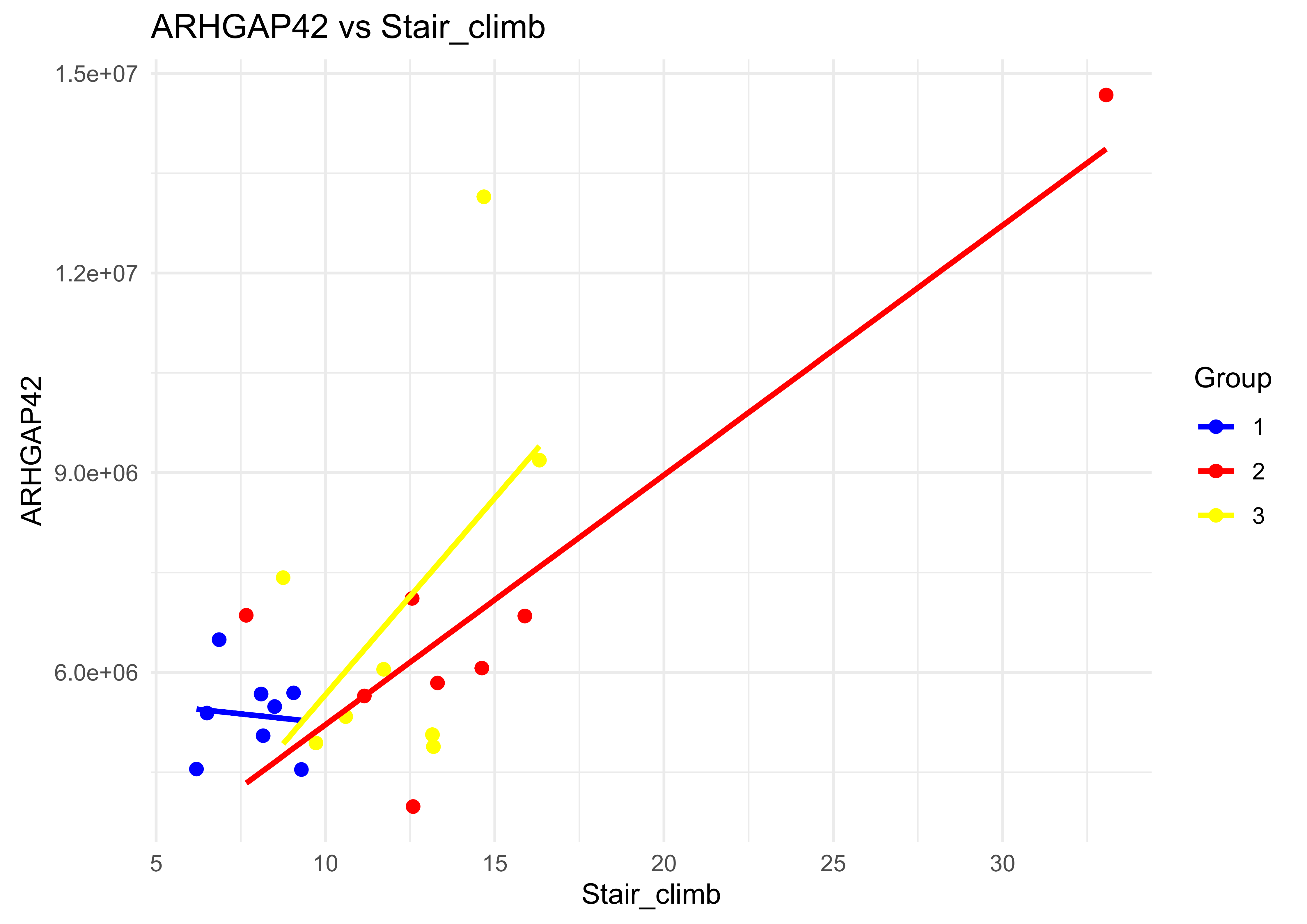
ARHGAP42 vs Stair_climb
ARHGAP42
Stair_climb
1.5e+07
1.2e+07
9.0e+06
6.0e+06
5
10
15
20
25
30
Group
1
2
3

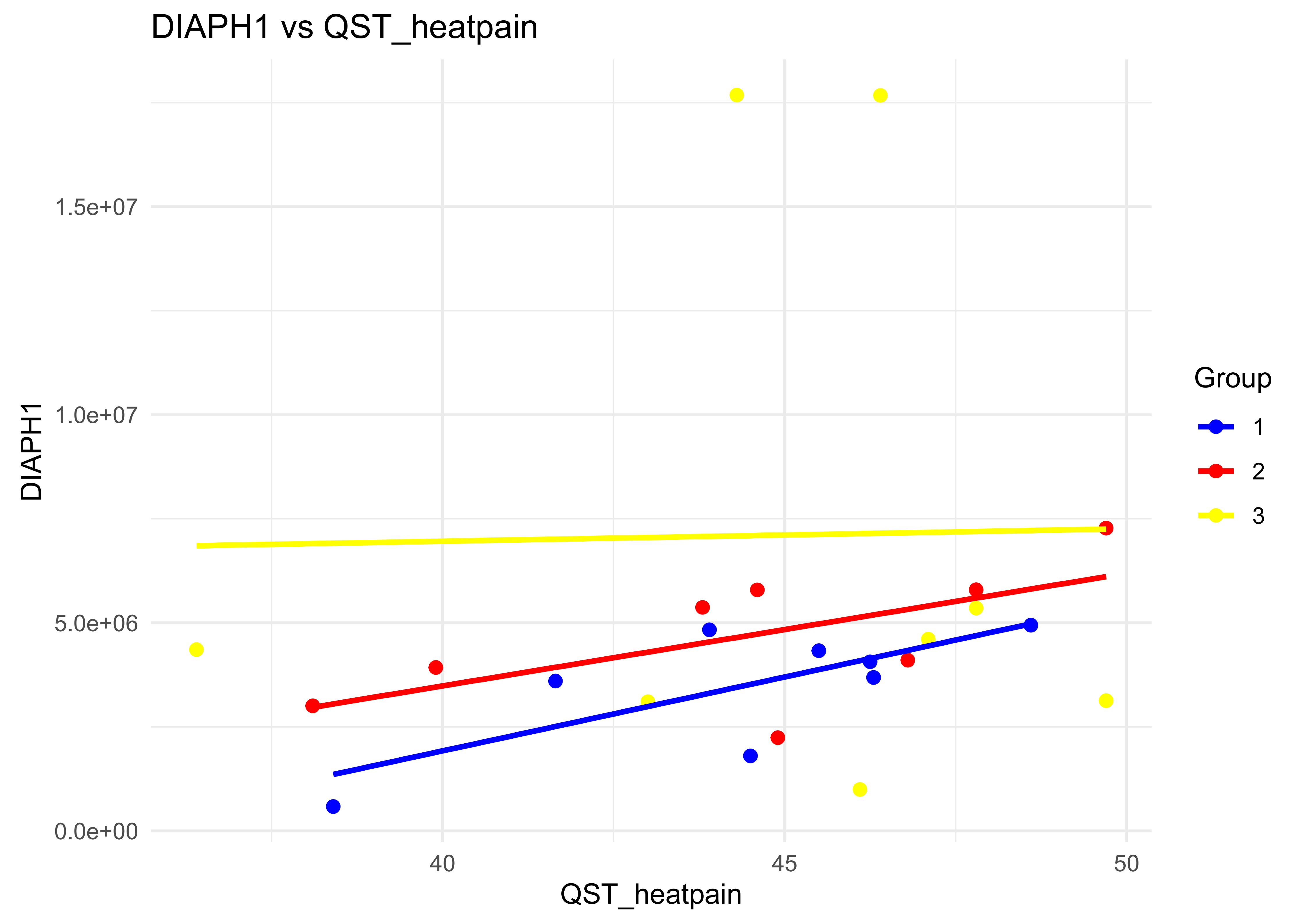

DIAPH1 vs QST_heatpain
DIAPH1
QST_heatpain
1.5e+07
1.0e+07
5.0e+06
0.0e+00
40
45
50
Group
1
2
3

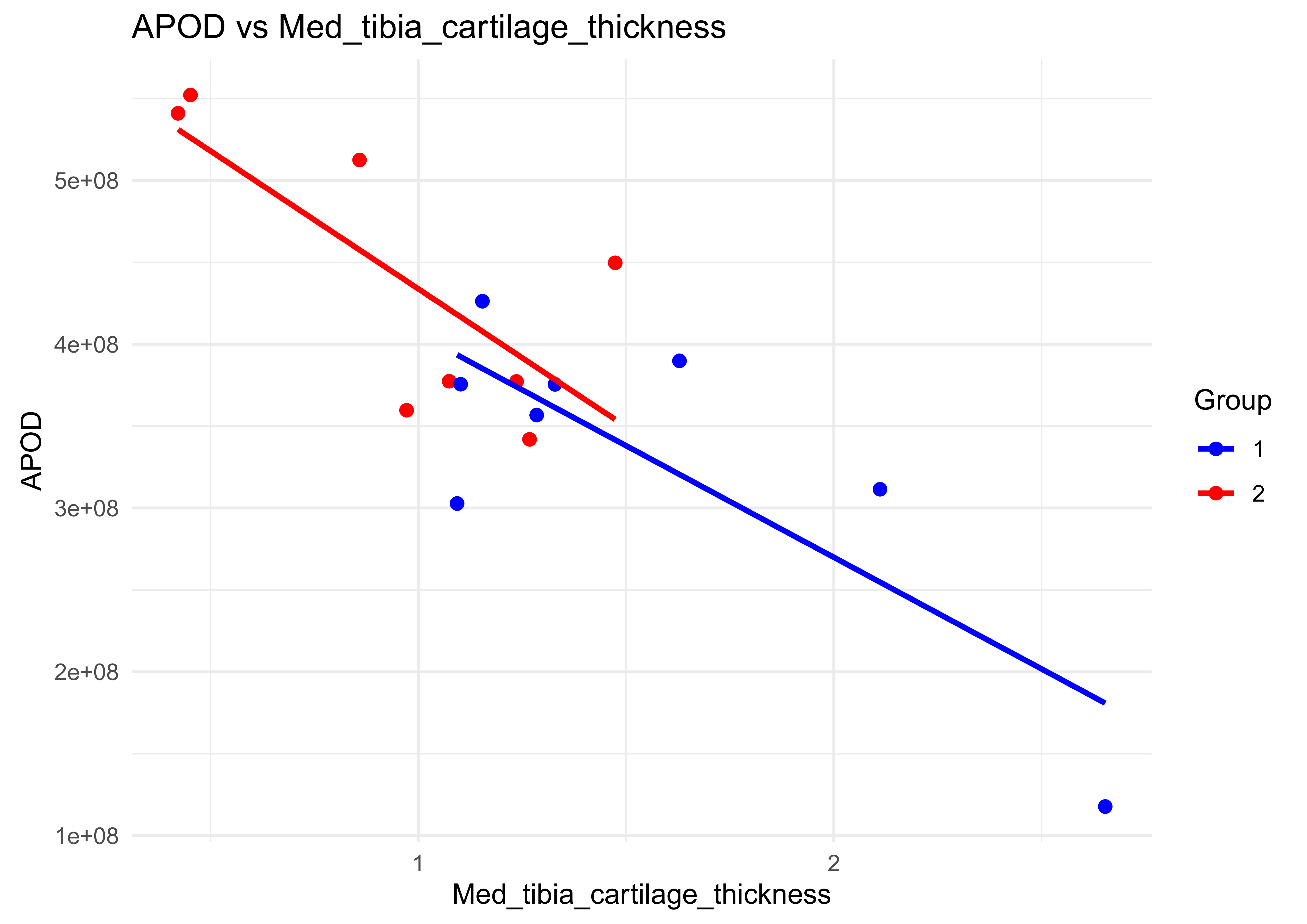
APOD vs Med_tibia_cartilage_thickness
APOD
5e+08
4e+08
3e+08
2e+08
1e+08
1
2
Med_tibia_cartilage_thickness
Group
1
2

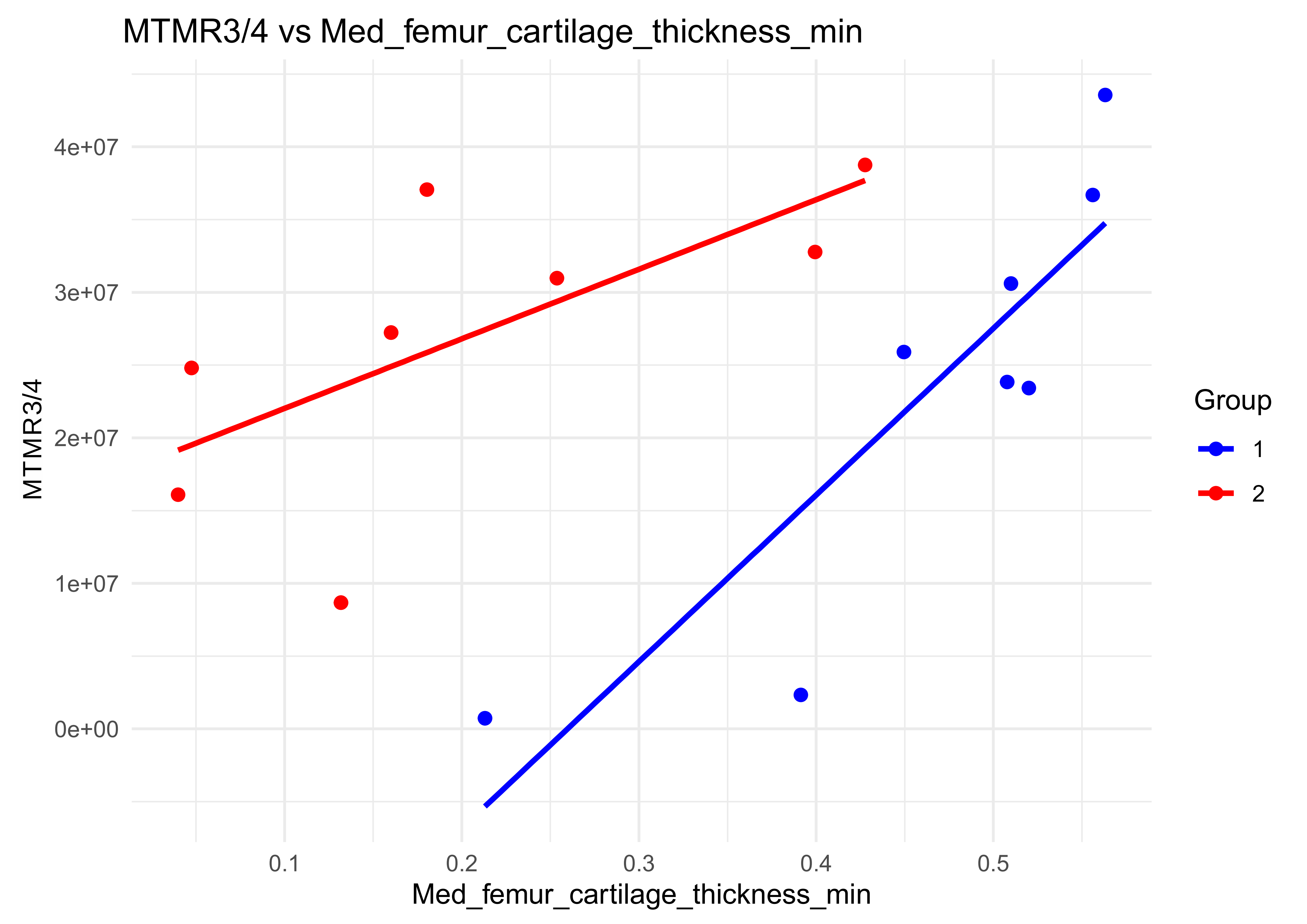

MTMR3/4 vs Med_femur_cartilage_thickness_min
4e+07
3e+07
2e+07
1e+07
0e+00
MTMR3/4
0.1
0.2
0.3
0.4
0.5
Med_femur_cartilage_thickness_min
Group
1
2
