# Supplementary material for: Proteomics Analysis Reveals Serum Biomarkers Reflecting Joint Pain and Physical Limitations in Knee Osteoarthritis Before and After Joint Replacement Surgery
Source: Cartilage. 2026 May 30:19476035261455413. Online ahead of print. doi: 10.1177/19476035261455413 (PMC13222223; doi:10.1177/19476035261455413)
Supplement: Supplemental material - Proteomics Analysis Reveals Serum Biomarkers Reflecting Joint Pain and Physical Limitations in Knee Osteoarthritis Before and After Joint Replacement Surgery [file sj-pdf-2-car-10.1177_19476035261455413.pdf]

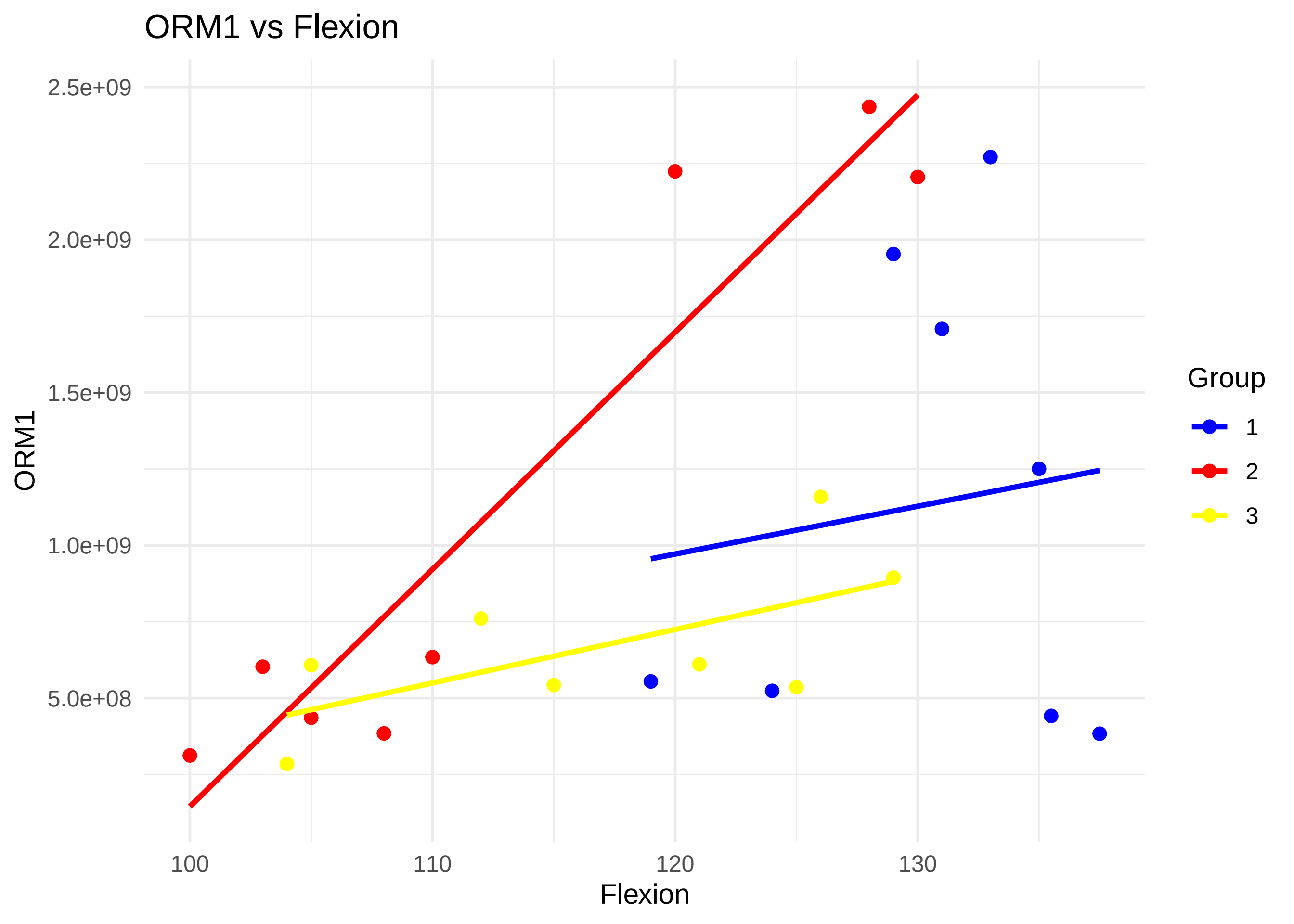
ORM1 vs Flexion
2.5e+09
2.0e+09
1.5e+09
1.0e+09
5.0e+08
ORM1
100
110
120
130
Flexion
Group
1
2
3

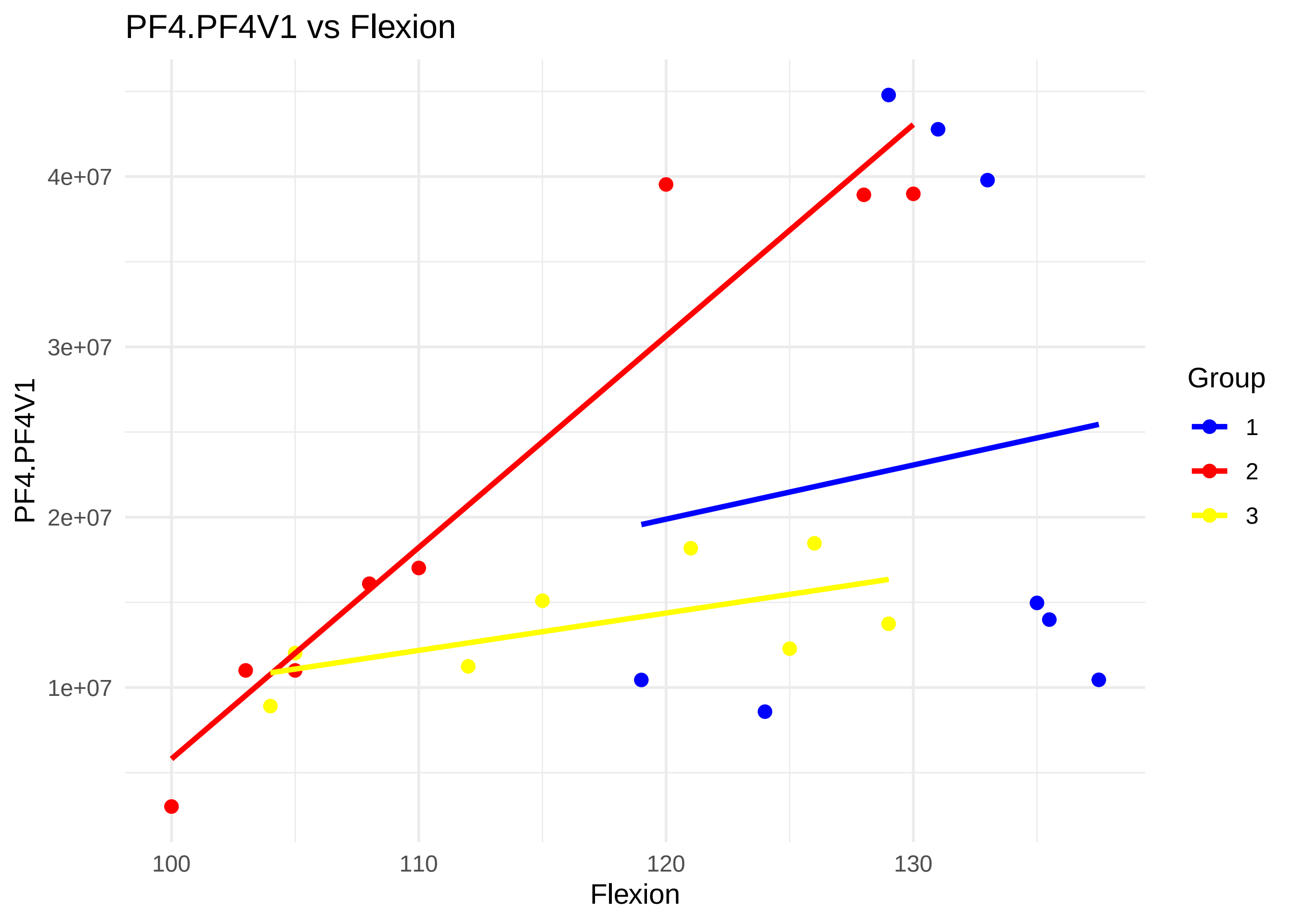
PF4.PF4V1 vs Flexion
PF4.PF4V1
4e+07
3e+07
2e+07
1e+07
100
110
120
130
Flexion
Group
1
2
3

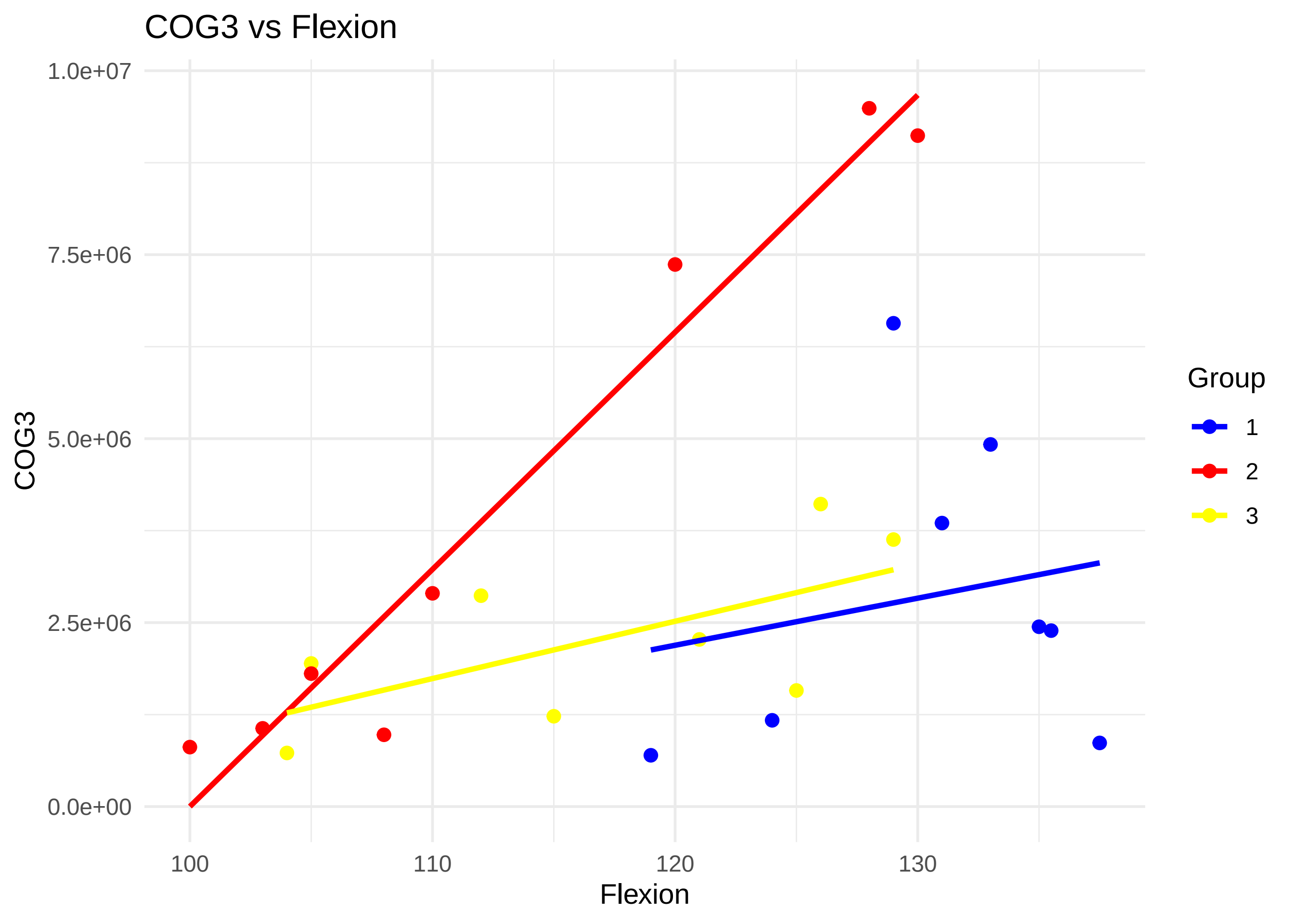

COG3 vs Flexion
1.0e+07
7.5e+06
5.0e+06
2.5e+06
0.0e+00
COG3
100
110
120
130
Flexion
Group
1
2
3

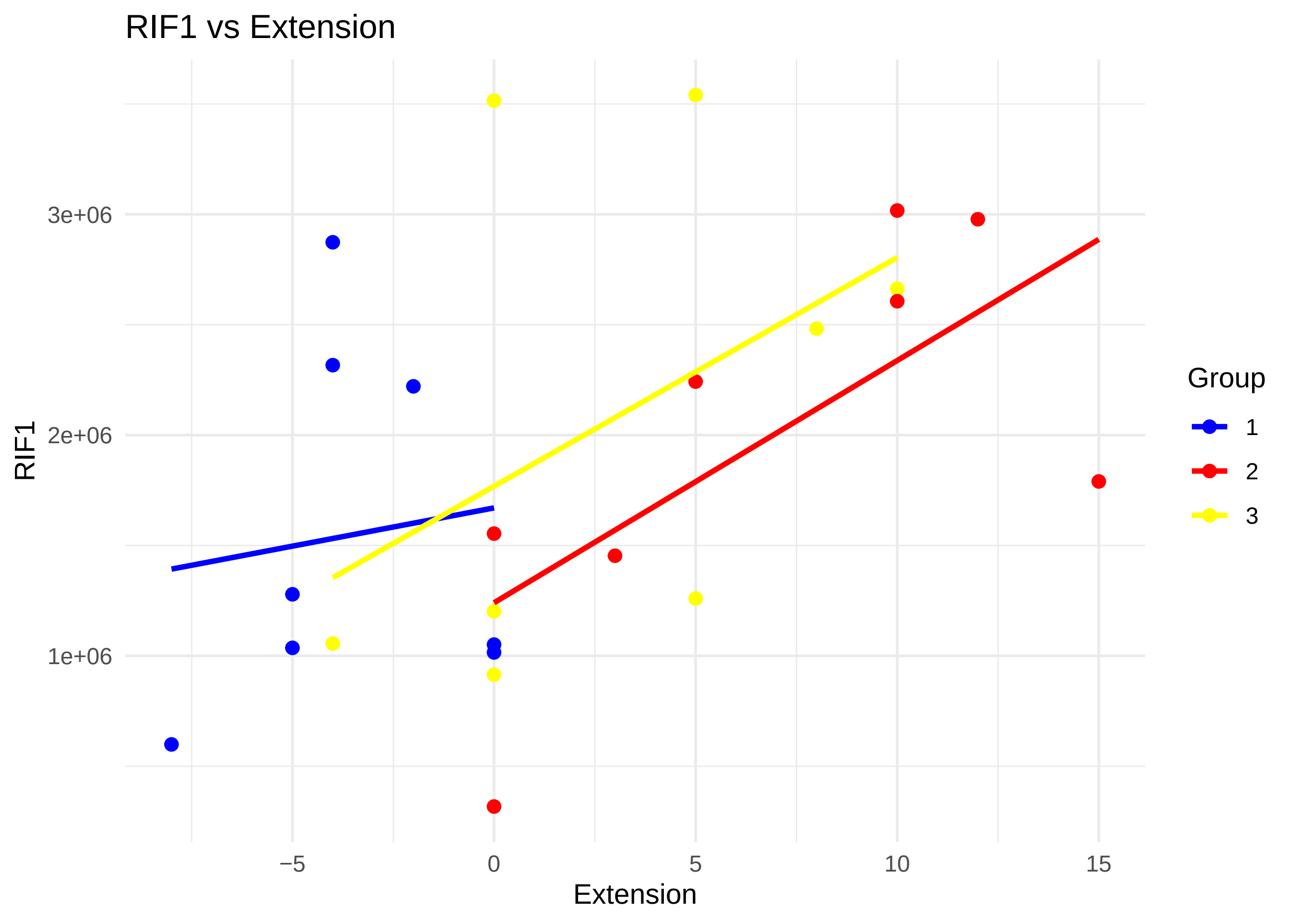

RIF1 vs Extension
3e+06
2e+06
1e+06
RIF1
−5
0
5
10
15
Extension
Group
1
2
3

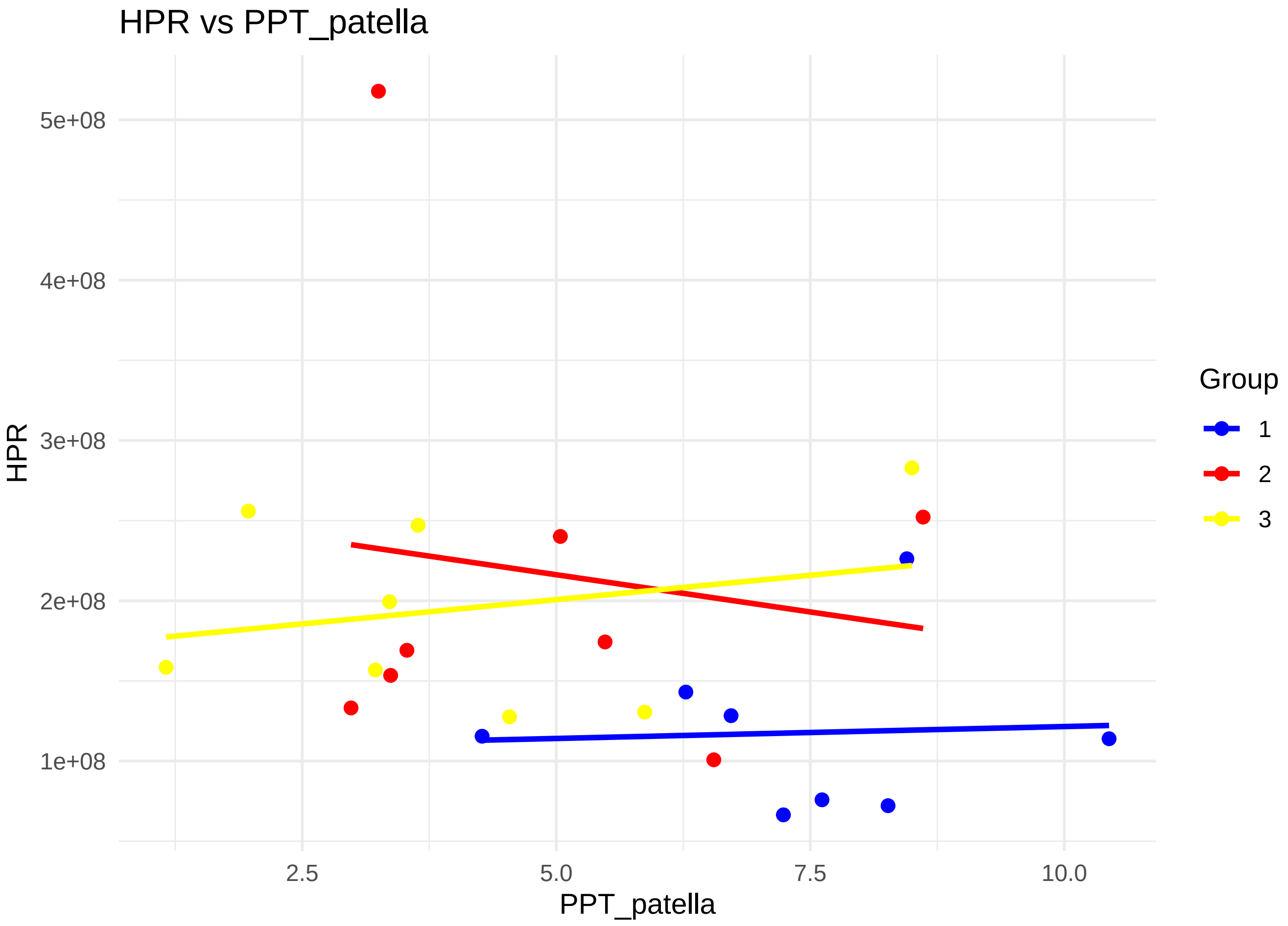
HPR vs PPT_patella
HPR
5e+08
4e+08
3e+08
2e+08
1e+08
2.5
5.0
7.5
10.0
PPT_patella
Group
1
2
3

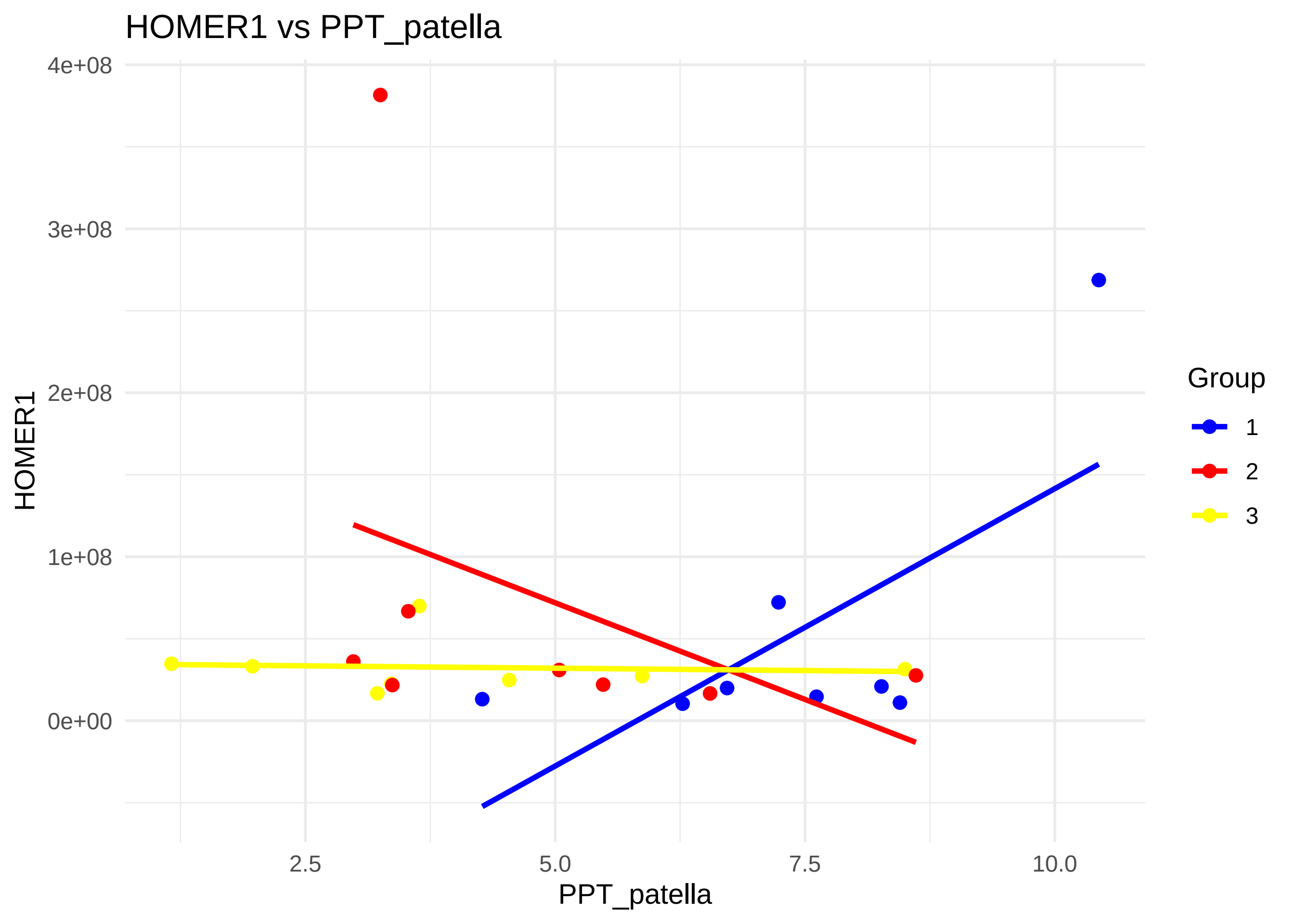

HOMER1 vs PPT_patella
4e+08
3e+08
2e+08
1e+08
0e+00
HOMER1
2.5
5.0
7.5
10.0
PPT_patella
Group
1
2
3

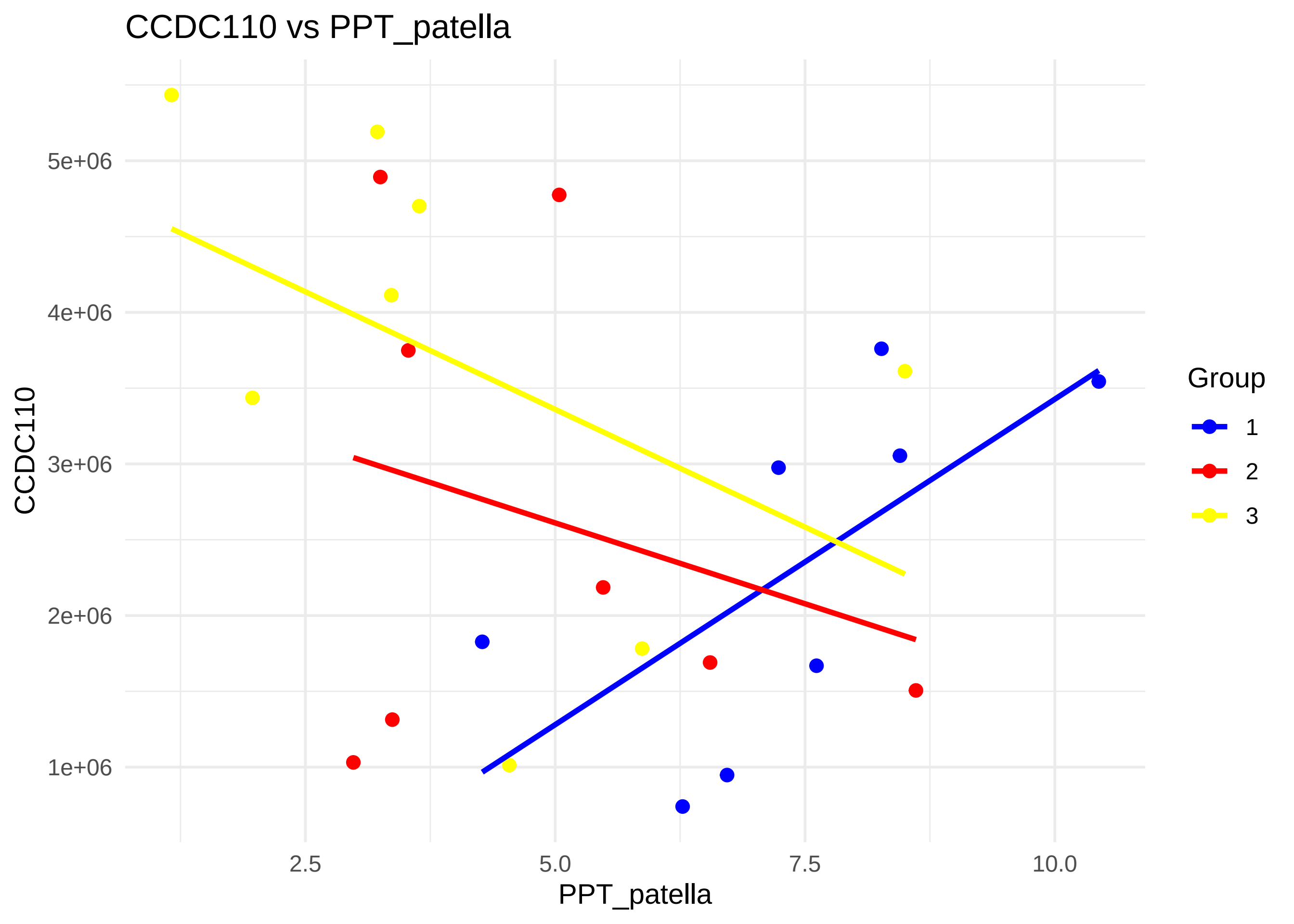

CCDC110 vs PPT_patella
5e+06
4e+06
3e+06
2e+06
1e+06
CCDC110
2.5
5.0
7.5
10.0
PPT_patella
Group
1
2
3

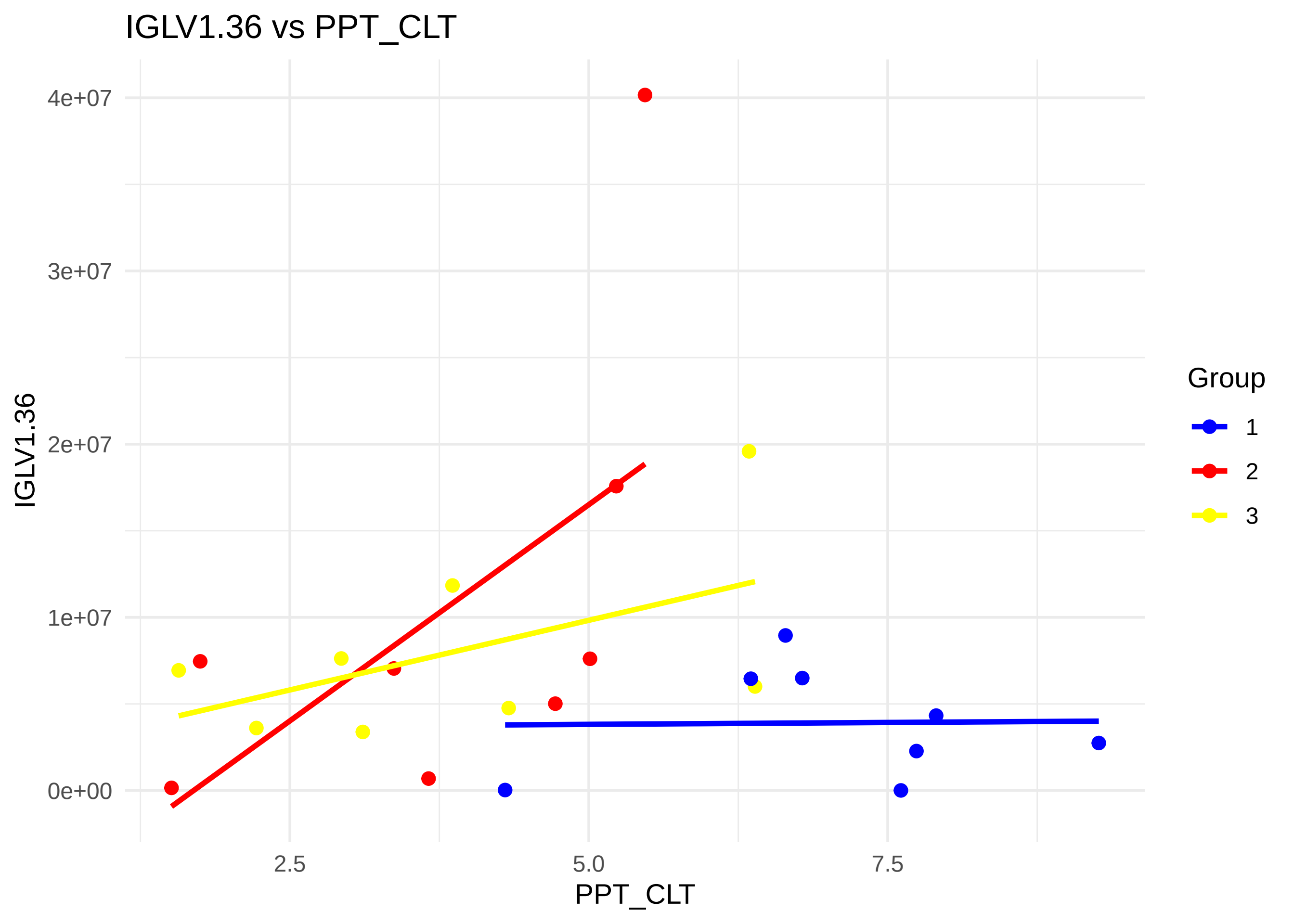

IGLV1.36 vs PPT_CLT
4e+07
3e+07
2e+07
1e+07
0e+00
IGLV1.36
2.5
5.0
7.5
PPT_CLT
Group
1
2
3

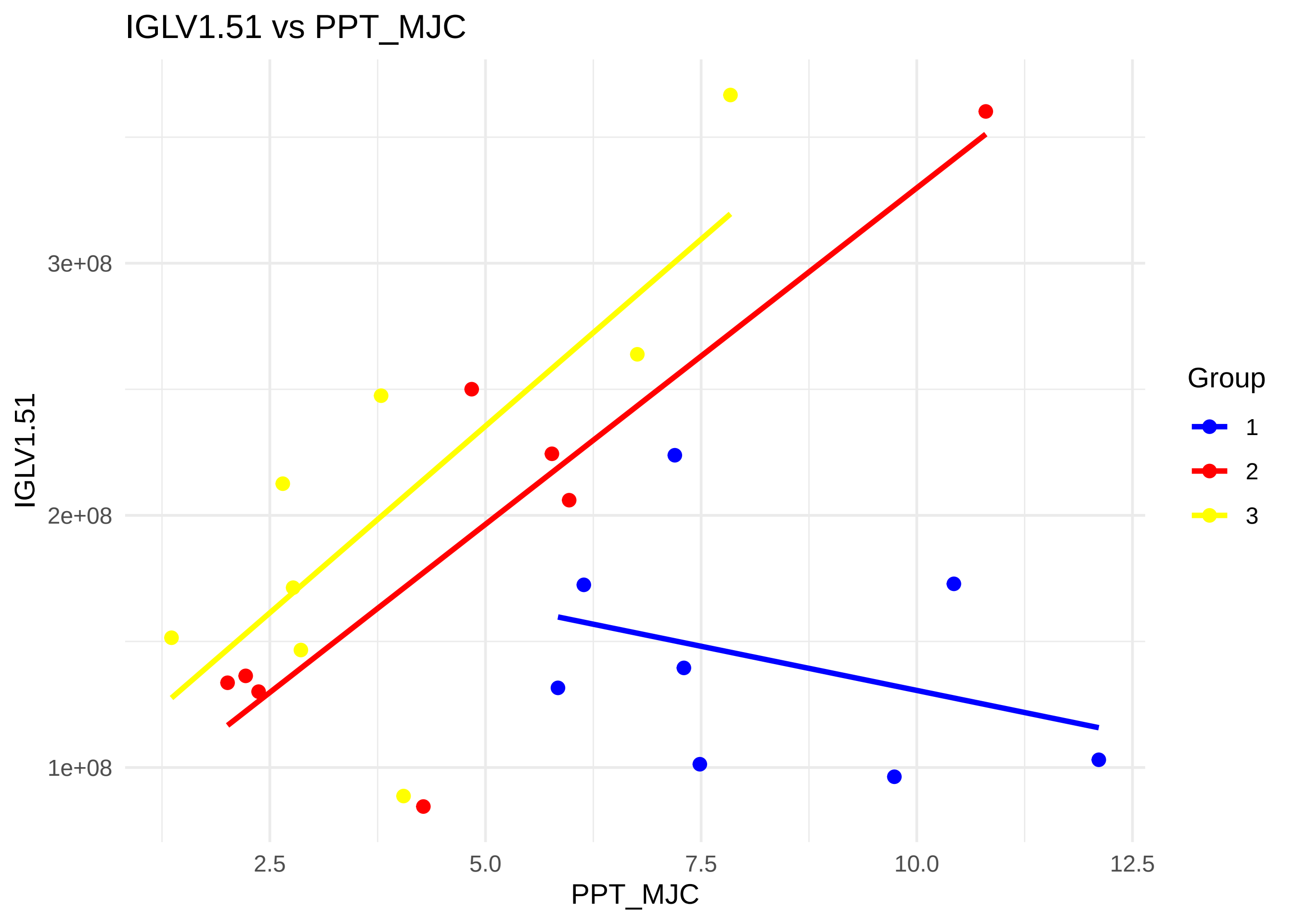

IGLV1.51 vs PPT_MJC
IGLV1.51
3e+08
2e+08
1e+08
2.5
5.0
7.5
10.0
12.5
PPT_MJC
Group
1
2
3

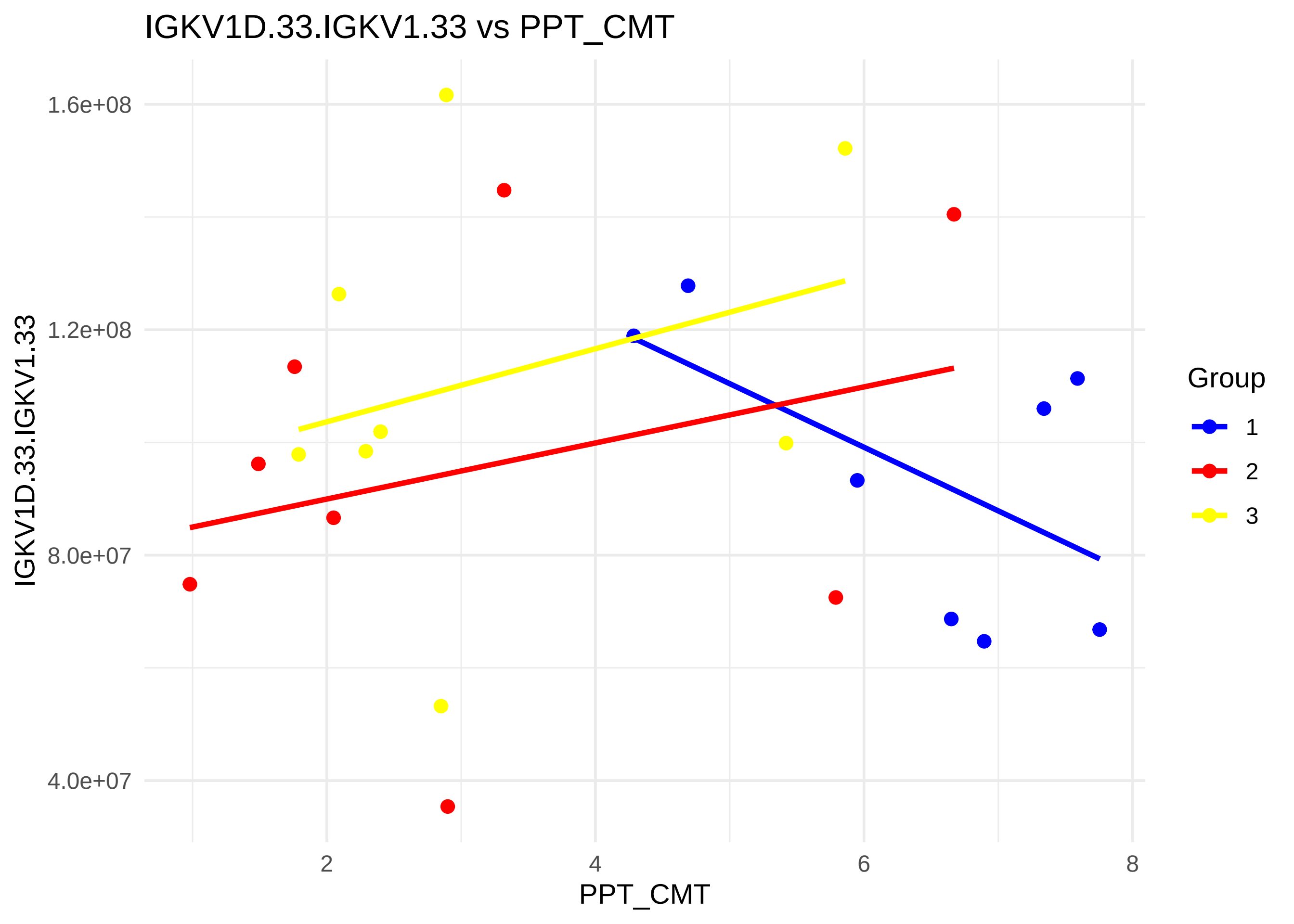

IGKV1D.33.IGKV1.33 vs PPT_CMT
1.6e+08
1.2e+08
8.0e+07
4.0e+07
IGKV1D.33.IGKV1.33
2
4
6
8
PPT_CMT
Group
1
2
3

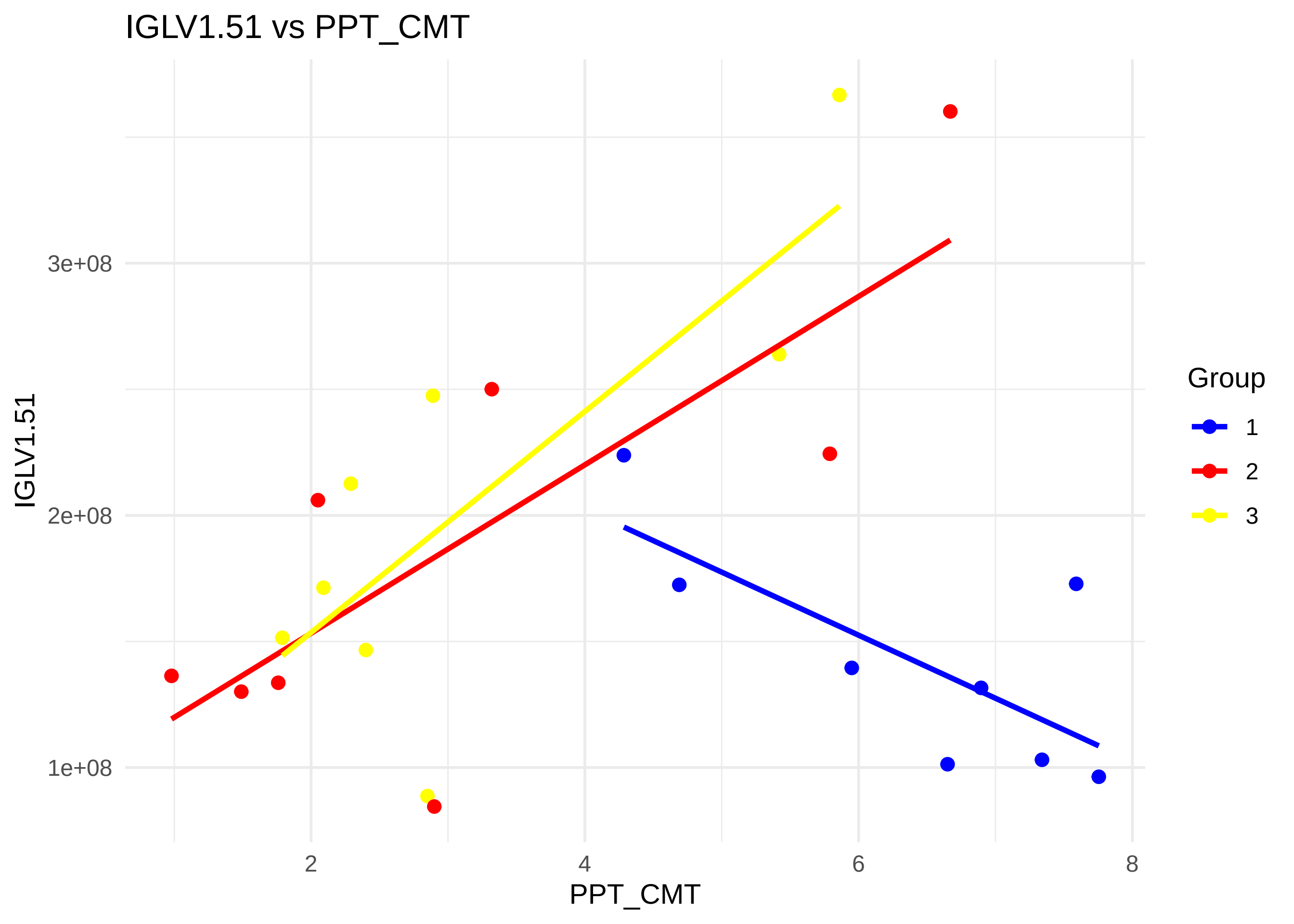

IGLV1.51 vs PPT_CMT
3e+08
2e+08
1e+08
IGLV1.51
2
4
6
8
PPT_CMT
Group
1
2
3

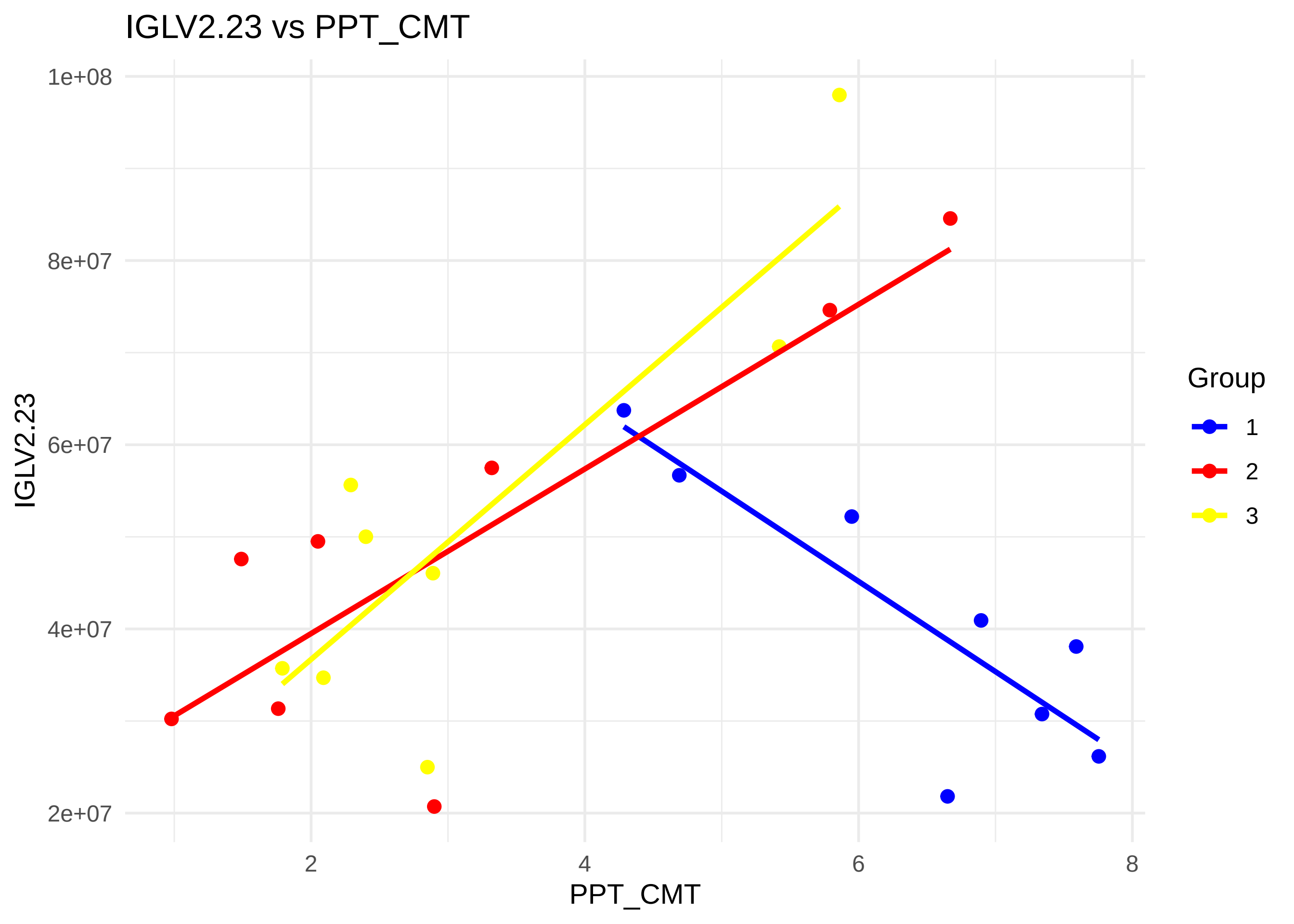
IGLV2.23 vs PPT_CMT
1e+08
8e+07
6e+07
4e+07
2e+07
IGLV2.23
2
4
6
8
PPT_CMT
Group
1
2
3

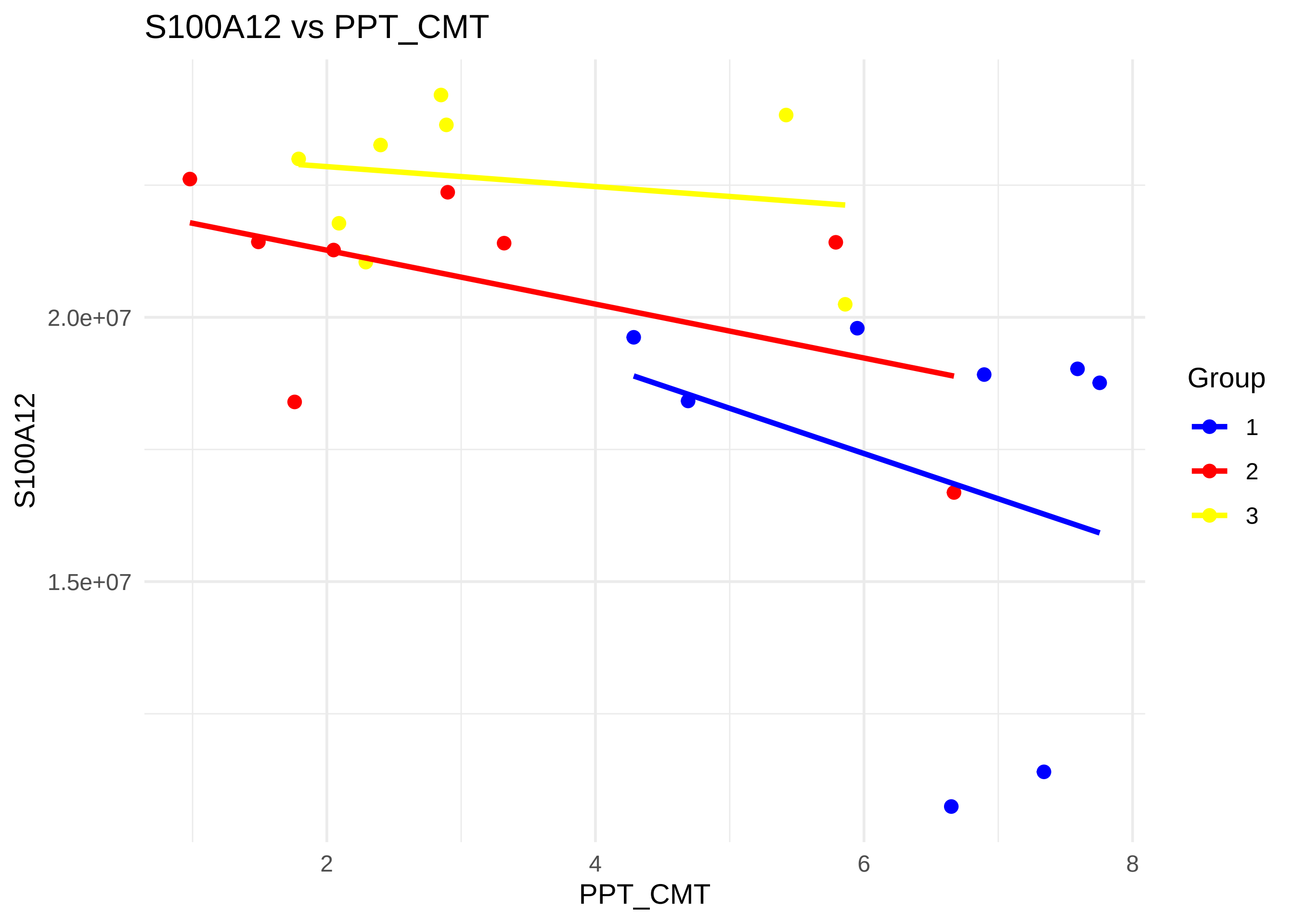
S100A12 vs PPT_CMT
S100A12
2.0e+07
1.5e+07
2
4
6
8
PPT_CMT
Group
1
2
3

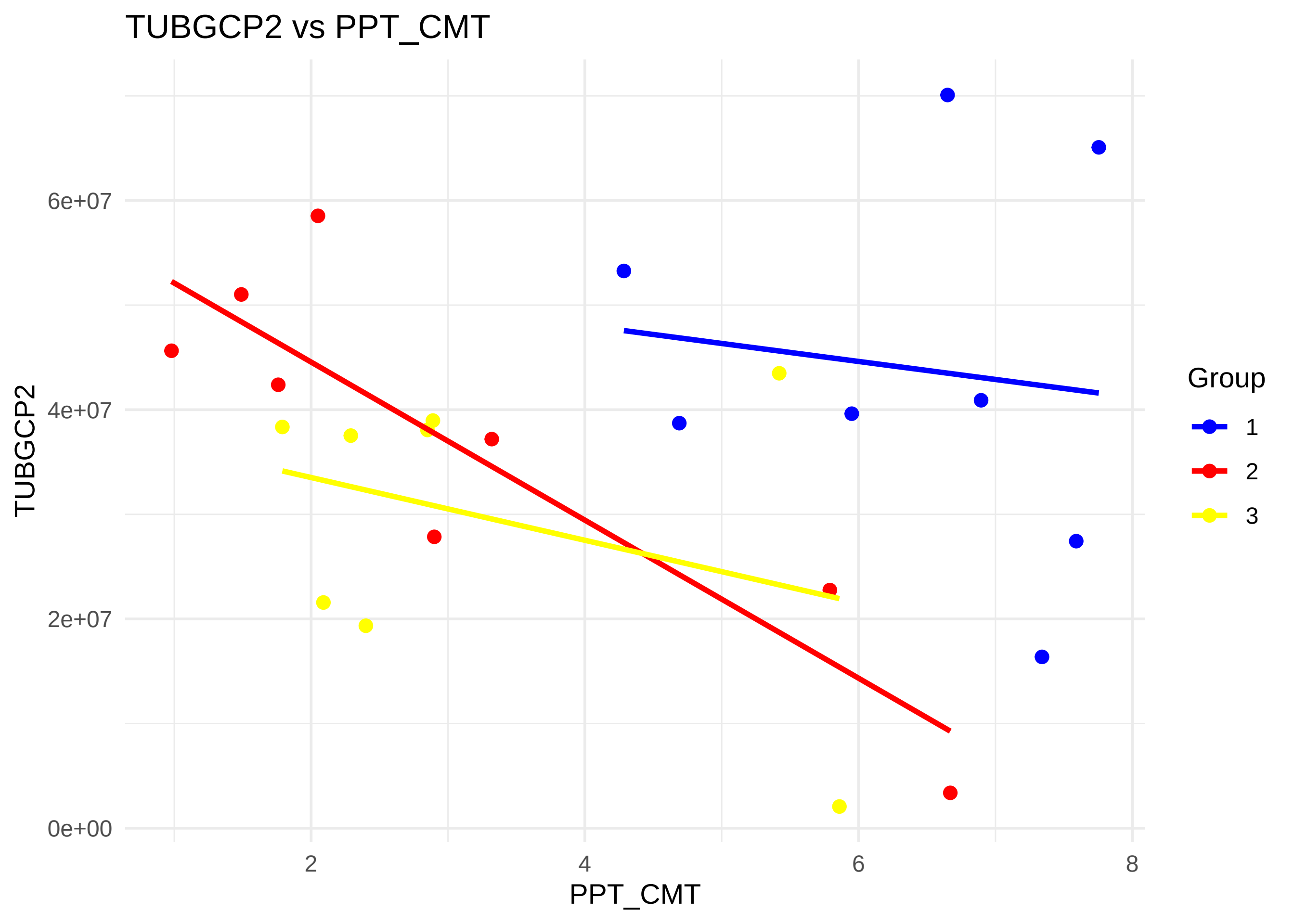

TUBGCP2 vs PPT_CMT
TUBGCP2
6e+07
4e+07
2e+07
0e+00
2
4
6
8
PPT_CMT
Group
1
2
3

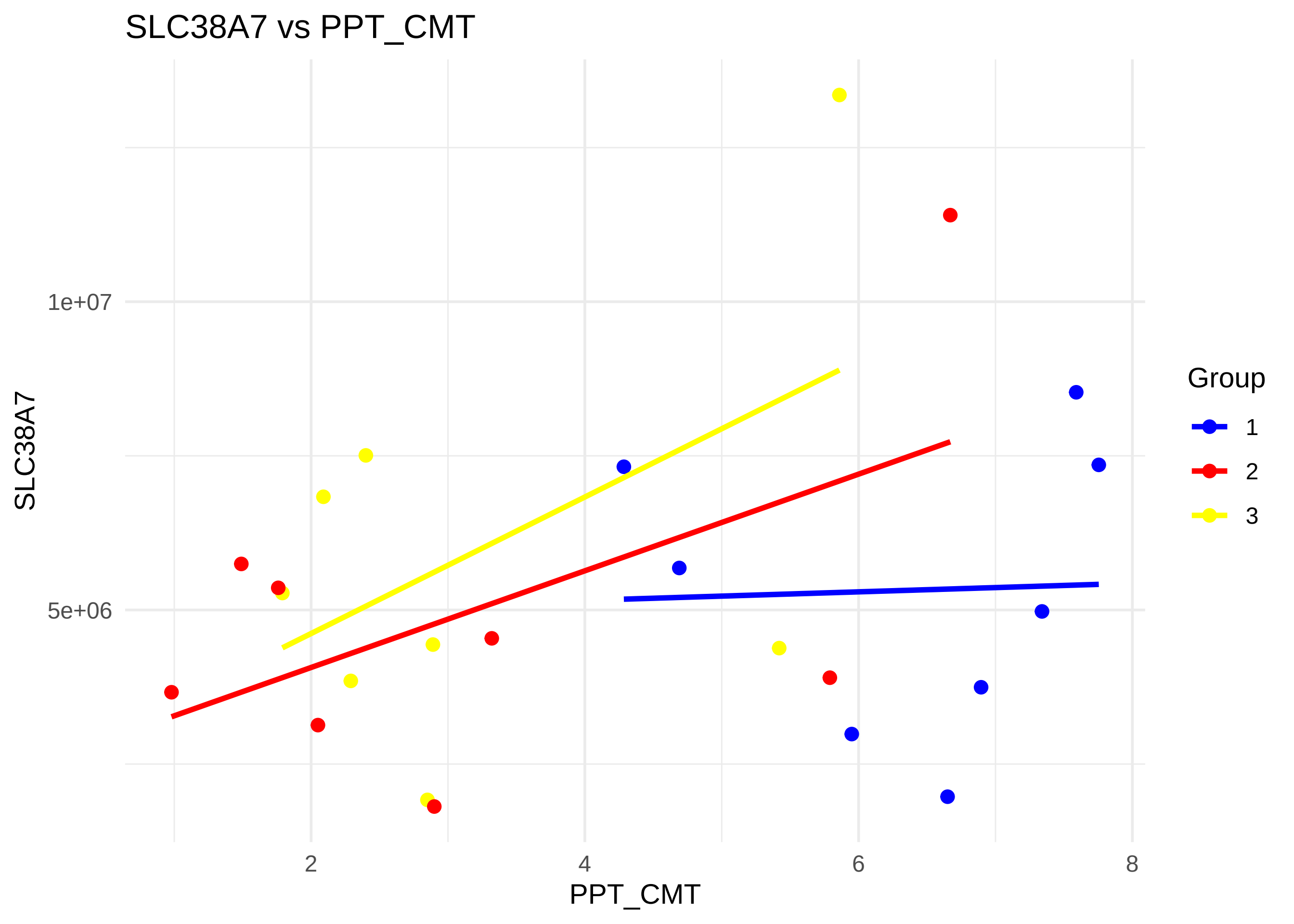
SLC38A7 vs PPT_CMT
SLC38A7
1e+07
5e+06
2
4
6
8
PPT_CMT
Group
1
2
3

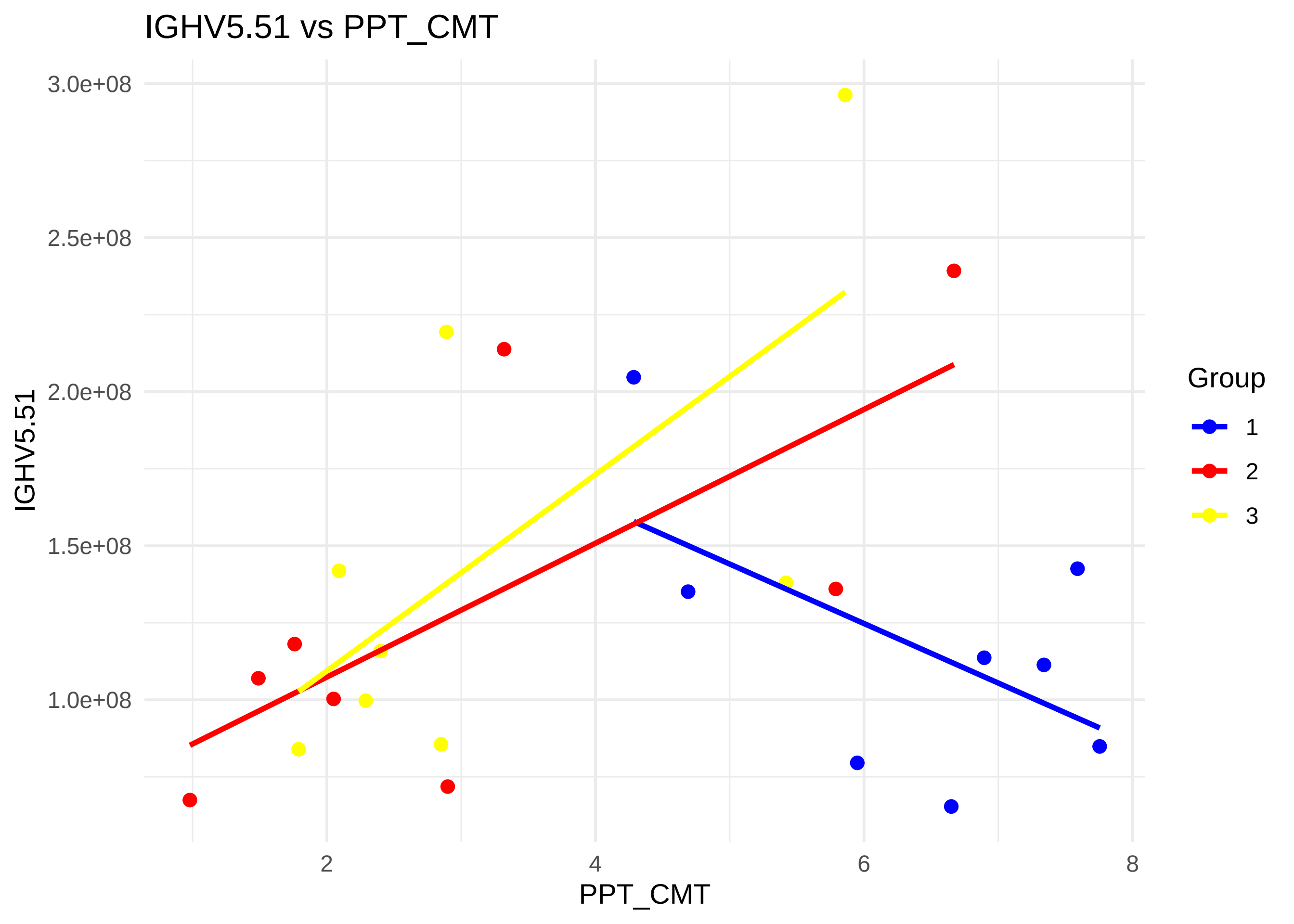

IGHV5.51 vs PPT_CMT
3.0e+08
2.5e+08
2.0e+08
1.5e+08
1.0e+08
IGHV5.51
2
4
6
8
PPT_CMT
Group
1
2
3

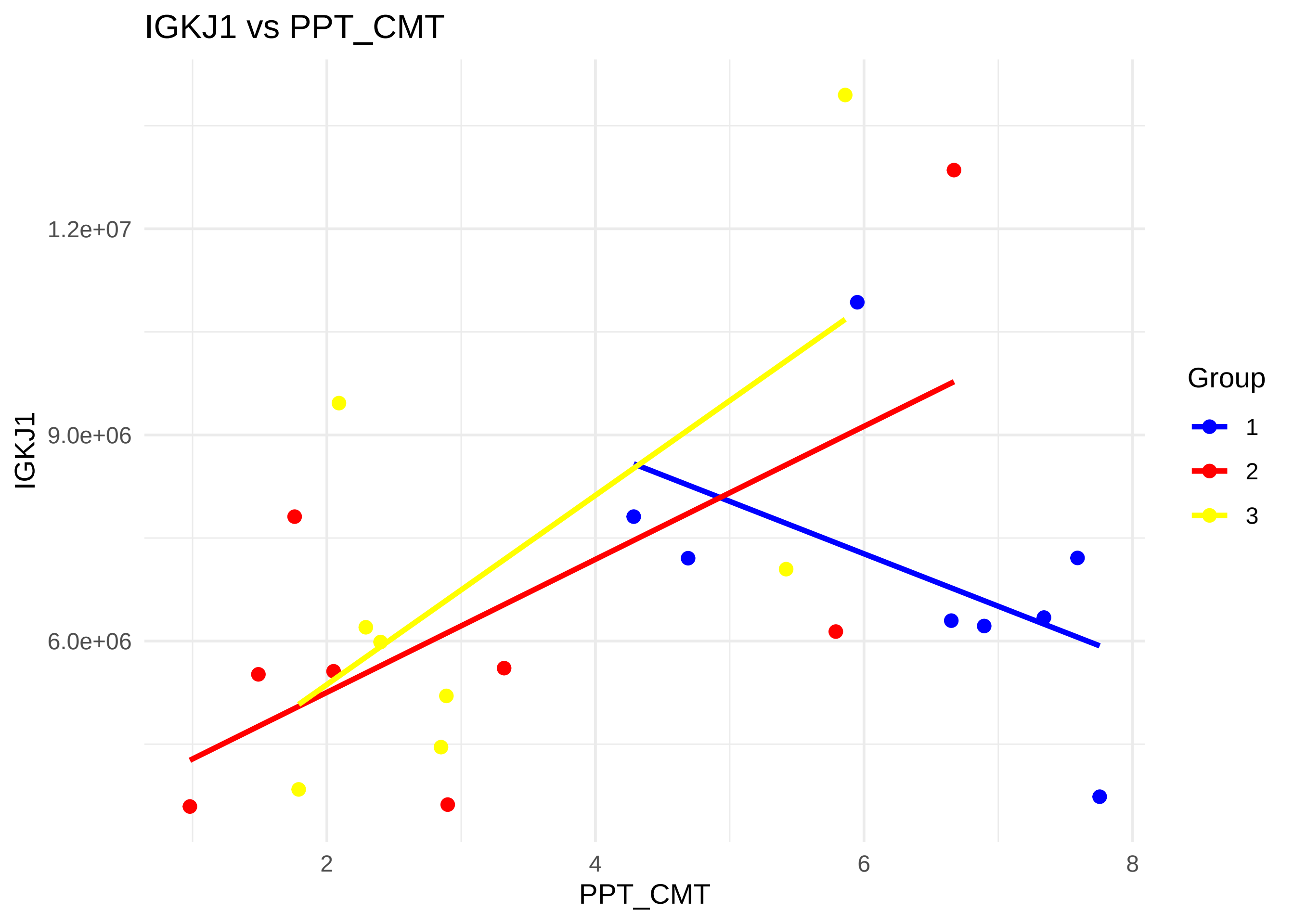
IGKJ1 vs PPT_CMT
1.2e+07
9.0e+06
6.0e+06
IGKJ1
2
4
6
8
PPT_CMT
Group
1
2
3

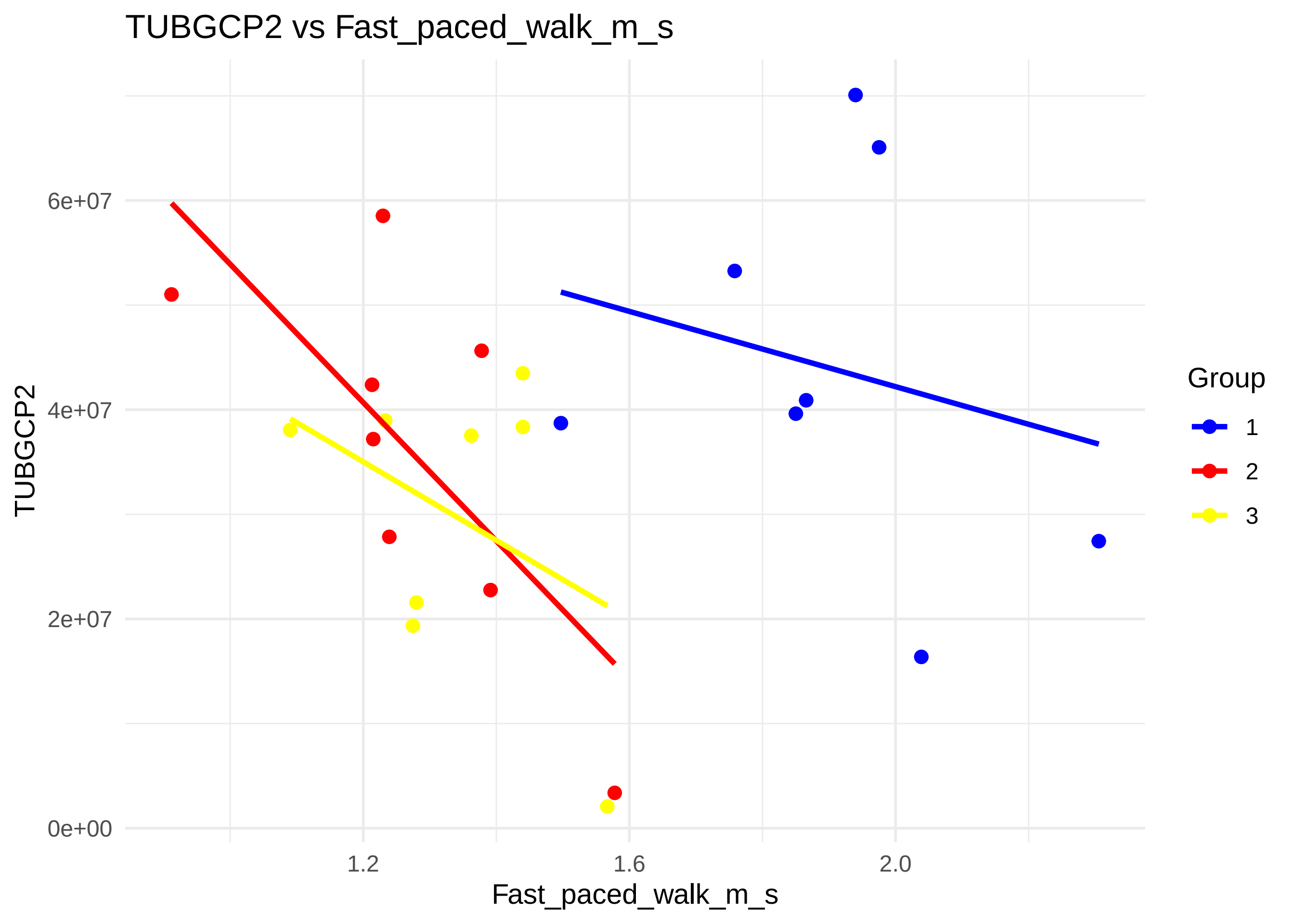
TUBGCP2 vs Fast_paced_walk_m_s
TUBGCP2
6e+07
4e+07
2e+07
0e+00
1.2
1.6
2.0
Fast_paced_walk_m_s
Group
1
2
3

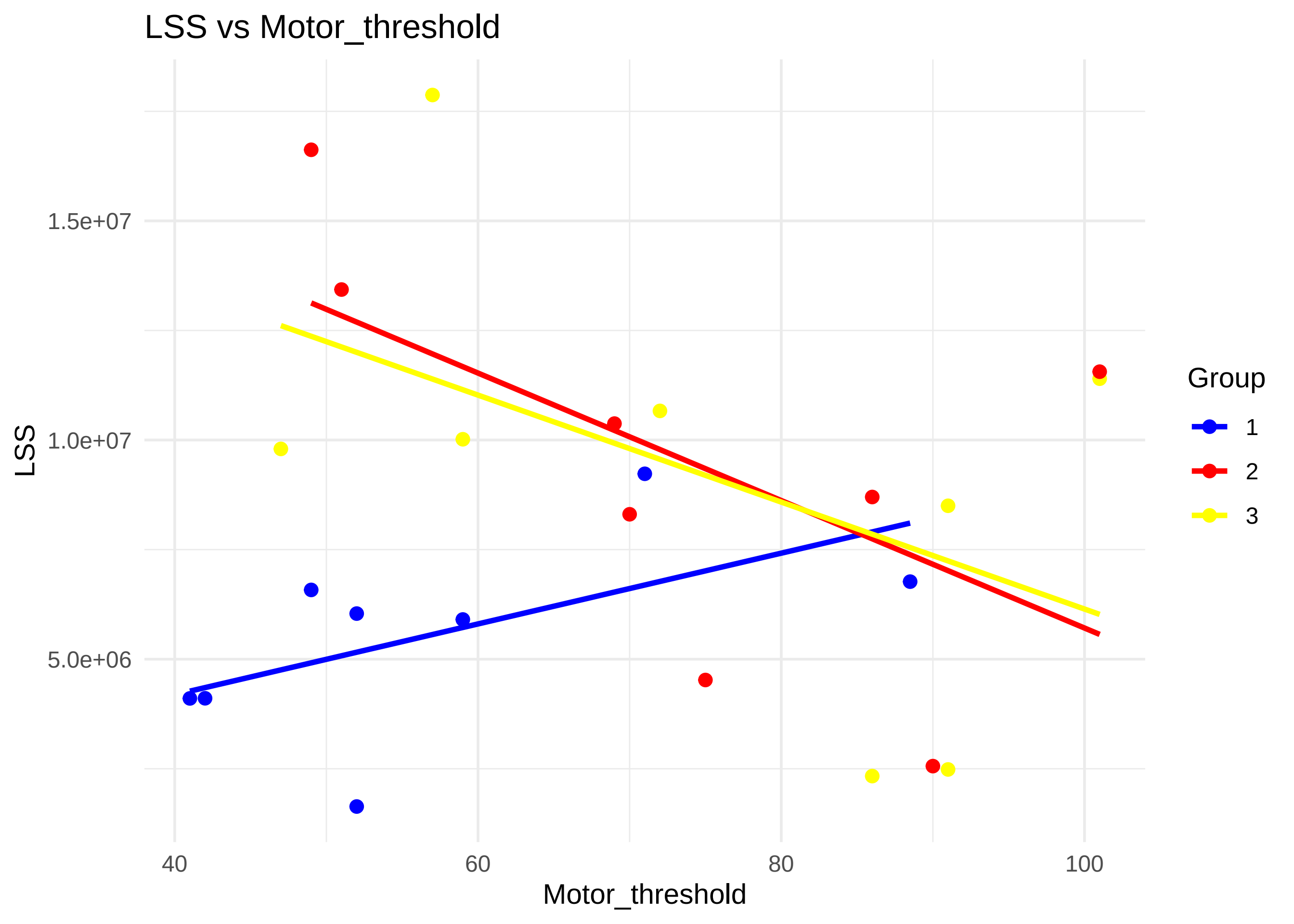
LSS vs Motor_threshold
LSS
1.5e+07
1.0e+07
5.0e+06
40
60
80
100
Motor_threshold
Group
1
2
3

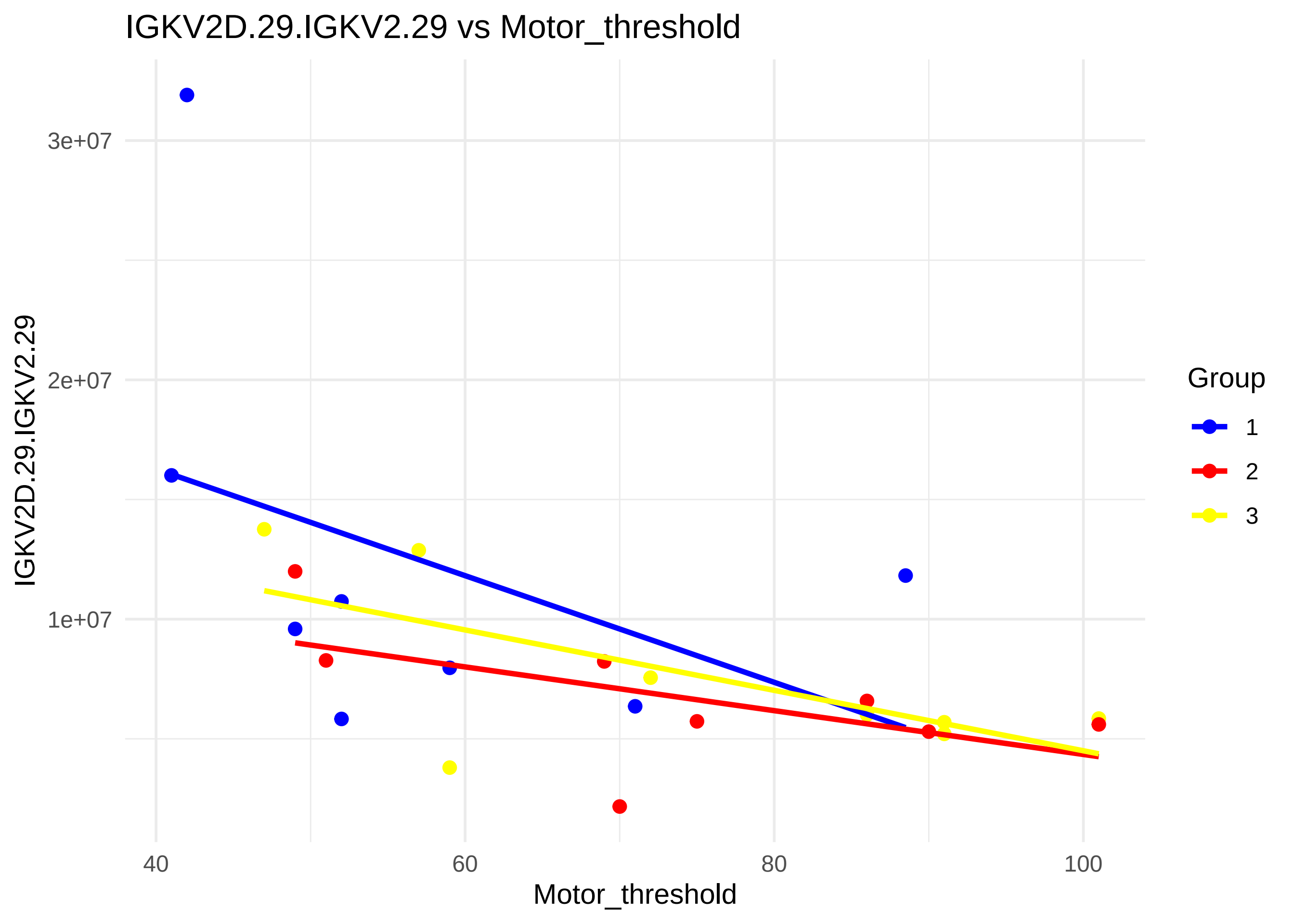

IGKV2D.29.IGKV2.29 vs Motor_threshold
3e+07
2e+07
1e+07
IGKV2D.29.IGKV2.29
40
60
80
100
Motor_threshold
Group
1
2
3

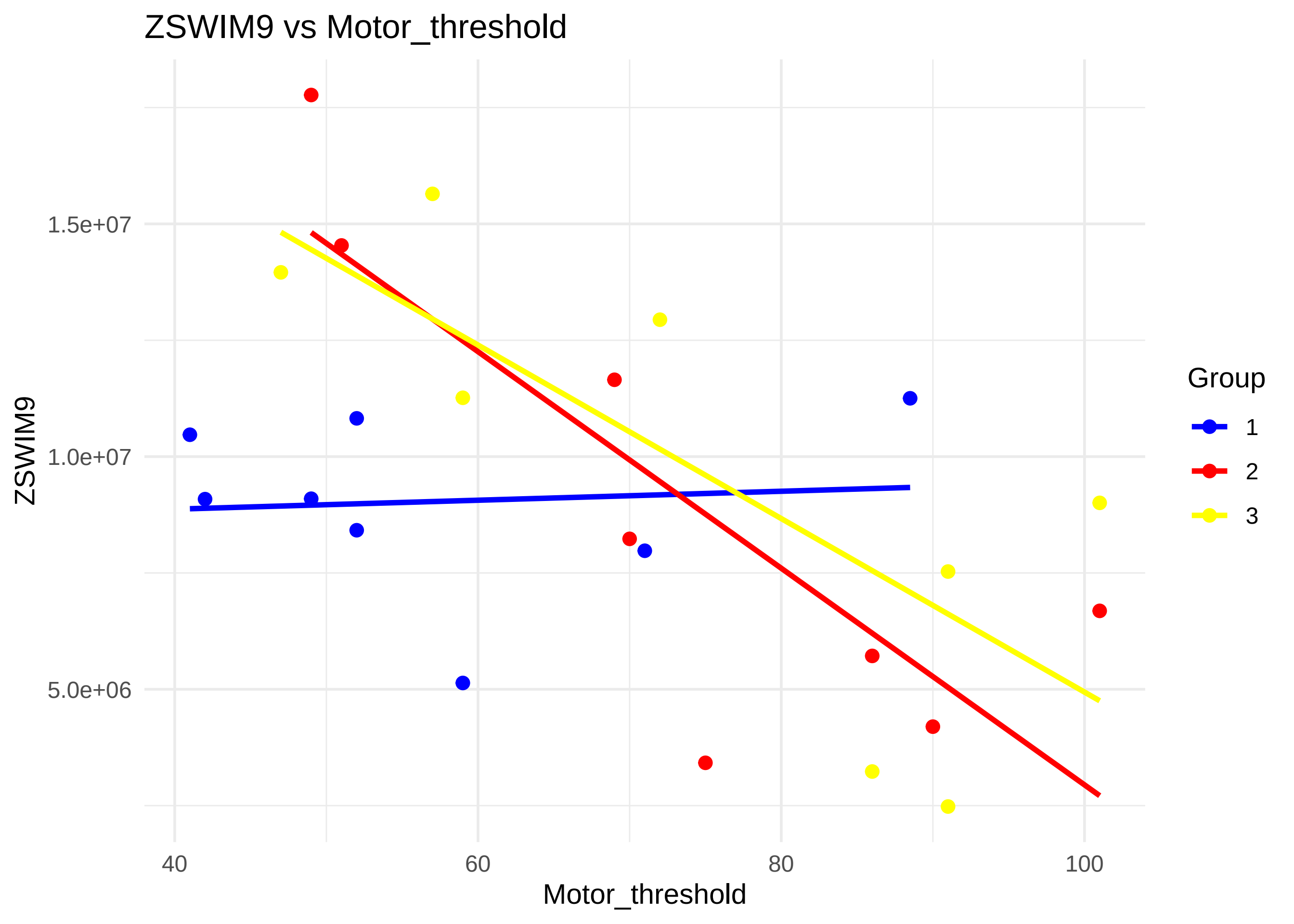
ZSWIM9 vs Motor_threshold
ZSWIM9
1.5e+07
1.0e+07
5.0e+06
40
60
80
100
Motor_threshold
Group
1
2
3

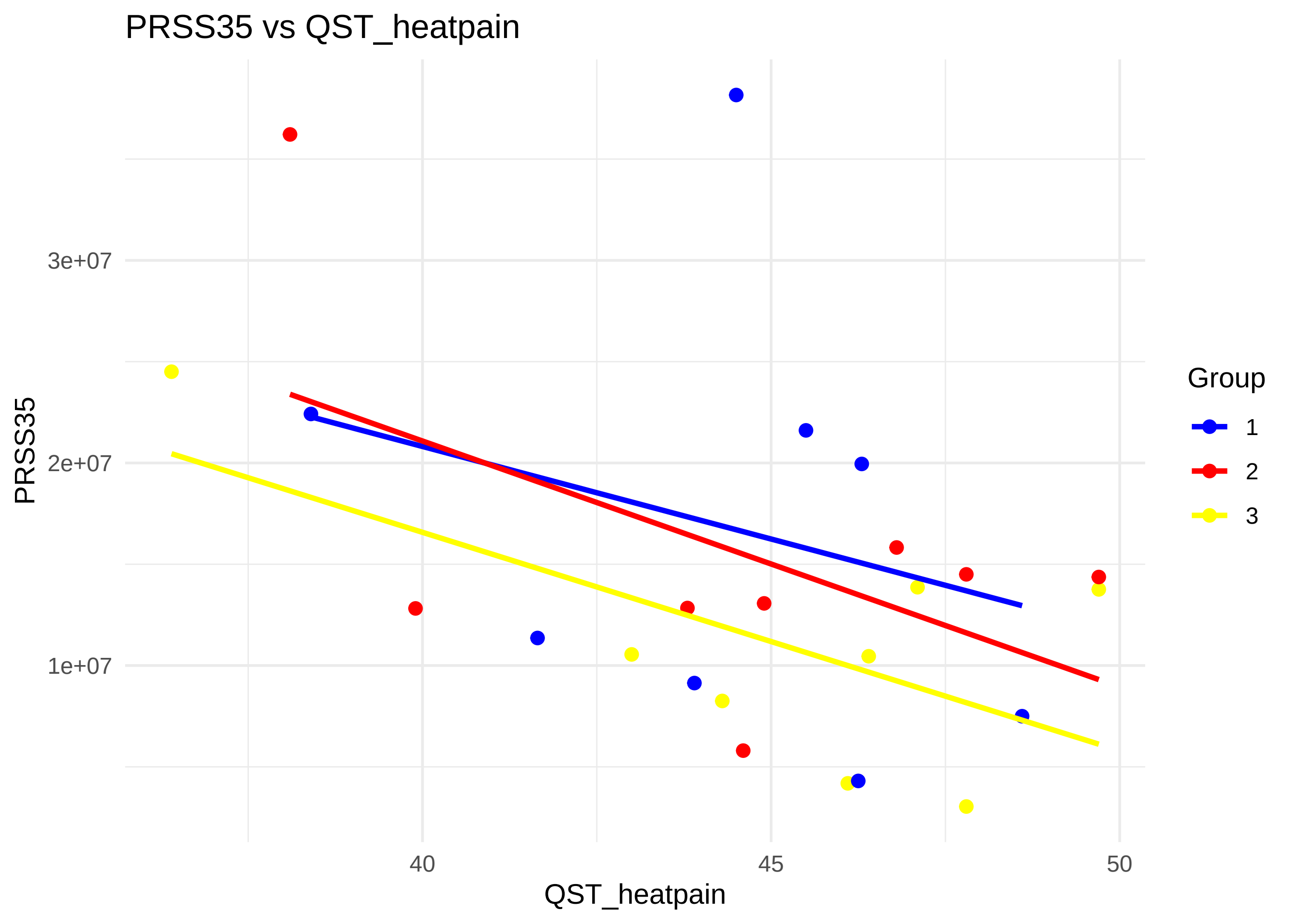

PRSS35 vs QST_heatpain
3e+07
2e+07
1e+07
PRSS35
40
45
50
QST_heatpain
Group
1
2
3
