# Supplementary material for: Proteomics Analysis Reveals Serum Biomarkers Reflecting Joint Pain and Physical Limitations in Knee Osteoarthritis Before and After Joint Replacement Surgery
Source: Cartilage. 2026 May 30:19476035261455413. Online ahead of print. doi: 10.1177/19476035261455413 (PMC13222223; doi:10.1177/19476035261455413)
Supplement: Supplemental material - Proteomics Analysis Reveals Serum Biomarkers Reflecting Joint Pain and Physical Limitations in Knee Osteoarthritis Before and After Joint Replacement Surgery [file sj-pdf-3-car-10.1177_19476035261455413.pdf]

Supplementary Table S2. Significant Pearson correlations between serum proteins and clinical variables adjusted for sex, age, and body mass index in controls and patients with baseline knee osteoarthritis.

Gene	Protein	Clinical variable	Pearson correlation	Adj. p-value
PROX1	Prospero homeobox protein 1	TPD med	0.858853844	0.031490918
CNDP1	Beta-Ala-His dipeptidase	PPT patella	0.84950992	0.044359231
HIP1	Huntingtin-interacting protein 1	PPT LJC	–0.893971095	0.011240864
C9	Complement component C9	PPT LJC	–0.935800284	0.001878898
ADAMTS20	A disintegrin and metalloproteinase with thrombospondin motifs 20	PPT LJC	–0.897718157	0.011035379
MUC5B	Mucin-5B	PPT LJC	–0.900671339	0.011035379
DCD	Dermcidin	PPT CLT	0.86500182	0.025586092
PPP1R16A	Protein phosphatase 1 regulatory subunit 16A	Stair climb	0.901479197	0.011035379
ARHGAP42	Rho GTPase-activating protein 42	Stair climb	0.874264952	0.01755644
DIAPH1	Protein diaphanous homolog 1	QST heat pain	0.874351595	0.01755644
APOD	Apolipoprotein D	Med tibia cartilage thickness	–0.88924508	0.01238506
MTMR3/4	Phosphatidylinositol-3,5-bisphosphate 3-phosphatase MTMR3/4	Min med femur cartilage thickness	0.884602932	0.01238506

TPD = two-point discrimination, med = medial, PPT = pressure pain threshold, LJC = lateral joint capsule, CLT = lateral tibial condyle, QST = quantitative sensory testing, min = minimum
