# Supplementary material for: Proteomics Analysis Reveals Serum Biomarkers Reflecting Joint Pain and Physical Limitations in Knee Osteoarthritis Before and After Joint Replacement Surgery
Source: Cartilage. 2026 May 30:19476035261455413. Online ahead of print. doi: 10.1177/19476035261455413 (PMC13222223; doi:10.1177/19476035261455413)
Supplement: Supplemental material - Proteomics Analysis Reveals Serum Biomarkers Reflecting Joint Pain and Physical Limitations in Knee Osteoarthritis Before and After Joint Replacement Surgery [file sj-pdf-4-car-10.1177_19476035261455413.pdf]

Supplementary Table S3. Significant Pearson correlations between serum proteins and clinical variables adjusted for sex, age, and body mass index in patients with baseline knee osteoarthritis (KOA) or post-surgical KOA.

Gene	Protein	Clinical variable	Pearson correlation	Adj. p-value
ORM1	Alpha-1-acid glycoprotein 1	Flexion	0.862496299	0.025626851
PF4/PF4V1	Platelet factor 4	Flexion	0.867285146	0.025626851
ORM2	Alpha-1-acid glycoprotein 2	Flexion	0.845194689	0.036745905
COG3	Conserved oligomeric Golgi complex subunit 3	Flexion	0.88374132	0.020022461
RIF1	Telomere-associated protein RIF1	Extension	0.869769571	0.025626851
PTPRC	Receptor-type tyrosine-protein phosphatase C	VAS pain	–0.822689569	0.046142951
HPR	Haptoglobin-related protein	PPT patella	–0.866533698	0.025626851
FGA	Fibrinogen alpha chain	PPT patella	–0.822402955	0.046142951
PPARG	Peroxisome proliferator-activated receptor gamma	PPT patella	–0.830067619	0.045237518
ZBTB46	Zinc finger and BTB domain-containing protein 46	PPT patella	–0.822187152	0.046142951
HOMER1	Homer protein homolog 1	PPT patella	–0.860824247	0.025626851
LMTK2	Serine/threonine-protein kinase LMTK2	PPT patella	–0.815969422	0.049835611
NELFB	Negative elongation factor B	PPT patella	–0.821059534	0.046142951
IGLV2-18	Immunoglobulin lambda variable 2-18	PPT patella	–0.825848747	0.046142951
PRR19	Proline-rich protein 19	PPT patella	–0.814671041	0.049891469
CCDC110	Coiled-coil domain-containing protein 110	PPT patella	–0.855534022	0.027935575
IGLV3-12	Immunoglobulin lambda variable 3-12	PPT patella	–0.814464772	0.049891469
C8A	Complement component C8 alpha chain	PPT LJC	0.835921686	0.041815832
TMEM198	Transmembrane protein 198	PPT LJC	–0.840210027	0.038837891
IGHV3-9	Immunoglobulin heavy variable 3-9	PPT CLT	–0.84185458	0.038837891
IGLV1-36	Immunoglobulin lambda variable 1-36	PPT CLT	0.852093943	0.030114334
KRT5	Keratin, type II cytoskeletal 5	PPT RF	–0.82390208	0.046142951
IGLV1-51	Immunoglobulin lambda variable 1-51	PPT MJC	0.864438626	0.025626851
TTR	Transthyretin	PPT MJC	–0.818762606	0.046326957
IGKV3D-20	Immunoglobulin kappa variable 3D-20	PPT MJC	0.839845847	0.038837891
IGKV1D-33/IGKV1-33	Immunoglobulin kappa variable 1D-33	PPT CMT	0.883553017	0.020022461
IGLV1-47	Immunoglobulin lambda variable 1-47	PPT CMT	0.818731549	0.046326957
IGLV1-51	Immunoglobulin lambda variable 1-51	PPT CMT	0.909216184	0.010026992
IGLV2-23	Immunoglobulin lambda variable 2-23	PPT CMT	0.86059125	0.025626851

AMBP	Protein AMBP	PPT CMT	0.823727965	0.046142951
S100A12	Protein S100-A12	PPT CMT	–0.912685639	0.010026992
SHROOM3	Protein Shroom3	PPT CMT	0.833168437	0.042071436
TUBGCP2	Gamma-tubulin complex component 2	PPT CMT	–0.826138125	0.046142951
CDCP1	CUB domain-containing protein 1	PPT CMT	–0.8449974	0.036745905
SLC38A7	Sodium-coupled neutral amino acid transporter 7	PPT CMT	0.860262549	0.025626851
KCNG1	Voltage-gated potassium channel regulatory subunit KCNG1	PPT CMT	0.821181954	0.046142951
IGHV5-51	Immunoglobulin heavy variable 5-51	PPT CMT	0.855664299	0.027935575
NKX1-1	NK1 transcription factor-related protein 1	PPT CMT	0.814911026	0.049891469
IGKJ1	Immunoglobulin kappa joining 1	PPT CMT	0.885704864	0.020022461
LINC03122	Uncharacterized protein encoded by LINC03122	PPT CMT	0.835128973	0.041898048
MNAT1	CDK-activating kinase assembly factor MAT1	Chair stand	0.850005413	0.031803779
CNTNAP4	Contactin-associated protein-like 4	Chair stand	0.833830755	0.042017186
TUBGCP2	Gamma-tubulin complex component 2	Fast-paced walk m/s	–0.868540716	0.025626851
PPP1R16A	Protein phosphatase 1 regulatory subunit 16A	Stair climb	0.836412159	0.041815832
CHRM3	Muscarinic acetylcholine receptor M3	Motor threshold	0.841740645	0.038837891
LSS	Lanosterol synthase	Motor threshold	–0.867333601	0.025626851
INF2	Inverted formin-2	Motor threshold	–0.819596456	0.046326957
IGKV2D-29/IGKV2-29	Immunoglobulin kappa variable 2D-29	Motor threshold	–0.864279554	0.025626851
IGKV3D-15	Immunoglobulin kappa variable 3D-15	Motor threshold	–0.822574831	0.046142951
ZSWIM9	Uncharacterized protein ZSWIM9	Motor threshold	–0.937815123	0.002299651
PIK3CA	Phosphatidylinositol 4,5-bisphosphate 3-kinase catalytic subunit alpha isoform	QST warm	0.841243115	0.038837891
FRYL	Protein furry homolog-like	QST heat pain	–0.814038395	0.049914964
CYP7A1	Cytochrome P450 7A1	QST heat pain	–0.821594016	0.046142951
MASP1	Mannan-binding lectin serine protease 1	QST heat pain	–0.821065347	0.046142951
ZNF618	Zinc finger protein 618	QST heat pain	–0.834545304	0.041898048
PRSS35	Inactive serine protease 35	QST heat pain	–0.854462175	0.028158137

VAS = visual analog scale, PPT = pressure pain threshold, LJC = lateral joint capsule, CLT = lateral tibial condyle, RF = *Rectus femoris* muscle, MJC = medial joint capsule, CMT = medial tibial condyle, QST = quantitative sensory testing
