# Supplementary material for: Proteomics Analysis Reveals Serum Biomarkers Reflecting Joint Pain and Physical Limitations in Knee Osteoarthritis Before and After Joint Replacement Surgery
Source: Cartilage. 2026 May 30:19476035261455413. Online ahead of print. doi: 10.1177/19476035261455413 (PMC13222223; doi:10.1177/19476035261455413)
Supplement: Supplemental material - Proteomics Analysis Reveals Serum Biomarkers Reflecting Joint Pain and Physical Limitations in Knee Osteoarthritis Before and After Joint Replacement Surgery [file sj-pdf-5-car-10.1177_19476035261455413.pdf]

Dear Editor,

Based on the Reviewers' reports we have now revised our manuscript (Manuscript ID CART-26-0027) accordingly. Please, see the specific comments and our responses below. The revised text is marked with the correction grid for your convenience.

Reviewer: 1

Thank you for the opportunity to review the manuscript entitled 'Proteomics analysis reveals serum biomarkers reflecting joint pain and physical limitations in knee osteoarthritis before and after joint replacement surgery'. The authors use serum proteomics to investigate potential biomarkers of knee osteoarthritis and correlate these to clinical features before and after arthroplasty. Given the low number of patients studied, the authors find striking changes in the serum proteome and perform further ontology and pathway interaction analysis. Overall, the manuscript is well written, and the content is suitable for the journal. Please find my these below:

1/ My major comment would be that the results section is made difficult to read by the jumping between Figures 2 and 3. I appreciate this is to focus on the different comparisons made but I suggest it would be better to group all figures related to a 'topic' together so they can be properly appreciated.

- Figures 2 and 3 were split and their panels rearranged to form Figures 2, 3, and 4 with revised legends (P33-34) to accommodate this comment.

2/ I would also suggest the up/down regulated proteins would be better displayed as a volcano plot where the authors could highlight particular proteins of interest. I would also consider removing (or moving to supplementary material) Figures 2 C,F, and I as it is very difficult to make any useful interpretation from these. Moreover, specific reference to these isn't made in the results text.

- The panels C and F of the original Fig. 2 were removed and replaced with newly-formed volcano plots of the three group comparisons (Fig. 2A, 3A, 4A), as suggested (P10, 12-13, 33-34).

3/ Similarly, whilst the STRING analysis is impressively networked, again, it is difficult to derive any meaning from these. The authors could consider highlighting specific pathways/networks identified (via GO) to make better inferences about the biological mechanisms at play in KOA.

- The Results section was revised according to this comment to clearly emphasize the most pivotal proteomic pathways and the central hub proteins with most interactions in each figure panel (P11-14).

4/ For the results obtained, I would also suggest the discussion is too long in the current form at 8 ½ pages and should be made more concise.

- We agree, and the Discussion section was shortened when possible and made more concise. The length was reduced by approximately 20%. The deleted passages have been removed and are therefore not shown in the correction grid.

5/ Finally, and more conceptually, the primary pathways that appear to be activated are those associated with generalised inflammation (eg complement and immune-related pathways). How would the authors suggest these might be meaningfully differentiated from other conditions like infection etc? If found the

discussion around the ECM remodelling, which one would assume is more targeted to KOA, much more compelling in this sense. Undeniably, complement (and associated) are showing strong changes in KOA and may be useful as therapeutic targets in treatment. However, I think the conclusions could be more realistically pitched to recognise the limits of generalised immune factors, strengths of targeted markers specific to the condition, and practicality/meaningfulness of panelling these together.

- This was also a valuable comment from the Reviewer, and the issue is now included in the Discussion section (P22). We agree that generalized inflammation pathways, such as complement and immune-related pathways, are not specific to KOA and may overlap with other conditions, such as infections. This highlights the value of markers linked, e.g., to ECM remodeling, which would be more directly reflective of joint-specific pathology.

6/ Minor comment – please check the flow rate of the UHPLC in the LC/MS section. Is 0.3mL/min potentially meant to be 0.3uL/min?

- The flow rate was checked by our proteomics core facilities, and the original 0.3 mL/min was verified to be correct (P6). This was an understandable question from the Reviewer, as in many labs there has been a recent shift to micro- or even nano-flow instruments, which improves the sensitivity of the analysis and reduces the required sample volumes.

Reviewer: 2

Proteomics analysis reveals serum biomarkers reflecting joint pain and physical limitations in knee osteoarthritis before and after joint replacement surgery

This study addresses a highly relevant clinical problem, namely the lack of effective disease-modifying therapies and reliable biomarkers for knee osteoarthritis, and is therefore of genuine interest with regard to both disease characterization and clinical outcome assessment. The authors used serum proteomics to compare controls with patients with end-stage KOA before surgery and 12 months after total knee arthroplasty, identifying marked alterations in proteins related to complement activation, immune and inflammatory pathways, coagulation, calcium homeostasis, and extracellular matrix remodeling. Complement factor I emerged as a promising biomarker candidate, and several proteins were associated with cartilage loss, pain sensitivity, physical function, and corticospinal excitability independently of sex, age, and BMI. The findings further suggest that systemic biochemical alterations are not fully normalized 12 months after surgery. Overall, the study appears clinically meaningful and timely, particularly given the clear need to define relevant biomarkers in KOA; however, the manuscript unfortunately has several important methodological and interpretative deficiencies that limit the strength of the conclusions.

Material&Methods:

The inclusion criteria require clearer definition and partly read more like a general cohort description than a rigorous eligibility framework. In particular, several terms are too vague for reproducibility, including “pain on most days,” “relatively normal range of motion,” “no clinical instability,” and “normal mental status,” as it is not explained how these were assessed or which thresholds or instruments were used. The use of Kellgren–Lawrence grade 2–4 also introduces substantial clinical heterogeneity, since grade 2 does not reflect end-stage disease, whereas patients undergoing TKA are typically more advanced. In addition, the broad age range further increases heterogeneity. Control and TKA groups appear to differ considerably in

age and BMI, which may strongly influence systemic metabolism, inflammation, and pain-related biology. These factors may therefore confound the reported biomarker differences and should be better justified and statistically addressed.

- We apologize that the inclusion/exclusion criteria were not clearer in the original submission. They have now been revised to be more specific and informative (P4). The indication for surgery was pain and functional impairment caused by KOA. According to the experienced orthopedic surgeon among our authors, the patients' pain and K–L grade do not always correlate very strongly. Patients referred for surgery may have significant pain that is refractory to conservative treatments even with the K–L grade 2. Experienced orthopedic surgeons opted to undergo surgery in mutual understanding with each patient.
- The differences in average age and BMI between the controls and KOA patients is unfortunate and sometimes unavoidable in OA research. These were taken into consideration by adjustments in our statistical analyses (regressions, Pearson correlations), and the issue is stated in the revised Statistical analyses section (P8) and Study limitations section (P22).

Line 90: was is meant by "an overnight fast"

- The data on the duration of fasting of each subject were revisited, and the average duration and range in hrs are now included in the revised manuscript (P4), as requested.

Line 91: KOA patients were sampled 12 month post-TKA – but what is about the control patients?

- The controls were sampled only once (at baseline), as explained now more clearly in the Material and Methods section (P4).

Line 103-113: the authors should think to transfer this information into a separate section because thes arre the PROMS and CROMs and contains information that ist missing throughout the information above - so it seems quite important – plus how were the ROMS being measured?

- The paragraph on MRI and the measurement of cartilage thickness was moved under a separate heading "Cartilage thickness measurement" (P5). The ROM was assessed using standard goniometric methods with the patient in the supine position. This is now stated in the revision (P5).

156-196: This paragraph describes MRI acquisition and image-processing methodology rather than statistical analysis. It is therefore not appropriate for the Statistics section. The actual statistical methods used for hypothesis testing and inference should be described separately and in sufficient detail.

- As requested, the methodologies of MRI acquisition and image-processing are now placed under a new heading "Cartilage thickness measurement" in the revision (P5), whereas the revised Statistical and bioinformatic analyses can be found in a separate section on P7-9.

RESULTS:

Line 202-205: The study design appears to include controls, baseline KOA patients, and the same KOA patients re-assessed 12 months after TKA. This is therefore not a simple two-group design. The statistical methods and group structure should be clarified explicitly. The comparison between controls and KOA patients 12 months after TKA may be informative, but it should be presented as a distinct contrast within a longitudinal framework, not as an ordinary independent-group comparison.

- We apologize that this issue was not clearly expressed in the original submission. The Statistical section has been revised to clarify the group comparisons (P7).

Line 199-205: where one can find the data described here? A note to a table or figure should be given.

- A reference to Supplementary Table S1 was added to this paragraph, as requested (P9).

Line 296-307: The correlation analysis needs clearer explanation. The authors state that Pearson correlation analysis was performed after adjusting for sex, age, and BMI, but it is not clear how this adjustment was done, since a standard Pearson correlation does not directly account for covariates. This should be clarified. In addition, no significant correlations were found when all three groups were analyzed together, whereas 13 significant correlations were identified when only controls and baseline KOA patients were included. This suggests that the observed associations may depend on the study groups included in the analysis and may not be consistent across the full cohort. The authors should explain this more clearly and describe how the multiple testing correction was performed.

- These important issues raised by the Reviewer are now more thoroughly explained in the revised statistical section (P8). The scatter plots of most significant protein–clinical variable pairs in the combined analysis of two groups (control and baseline KOA or baseline and post-surgical KOA) are now included as new Supplementary Figures S1–2. As suggested by the Reviewer, particular correlations can be quite different in the separate groups, which basically explains why the significance is often lost when the 3rd group is added to the analysis. Possible reasons for this include, e.g., differences in the strength or direction of the associations between variables in controls, and changes in the correlation structure or variability of the measurements following surgery. In the new Supplements, the correlations can be observed groupwise to accommodate this potential discrepancy.

Figure 4/5 are only partly effective in displaying the correlation results. Although the plots show the direction of the associations, the color legend suggests a continuous range of correlation values, while the points appear to be displayed mainly as red or blue without clear intermediate shades. This makes the color scale difficult to interpret and potentially misleading. The authors should either use a true continuous color encoding that matches the legend or simplify the legend to indicate only positive versus negative correlations. In addition, the meaning of point size should be clearly explained in the figure caption.

- The Figures 5 and 6 (previously 4 and 5) and their legends were revised to accommodate this perceptive point raised by the Reviewer (P34-35).

Further comments: the summary correlation plot is helpful as an overview, but it does not allow the reader to assess the underlying data, potential outliers, or whether the associations are truly linear. It would strengthen the manuscript to show scatter plots with regression lines for the most relevant correlations, rather than relying only on the bubble plot. If the reported results are based on partial correlations adjusted for sex, age, and BMI, the authors should also clarify how these adjusted associations are visualized. Additional plots could be provided in the supplementary material.

- The calculation of Pearson correlations is now more thoroughly explained in the revised statistical section (P8). The scatter plots of protein–clinical variable pairs that were most significant in the combined analysis of two groups (control and baseline KOA or baseline and post-surgical KOA) are now included as new Supplementary Figures S1–2 (P36).

Shorten discussion.

- The Discussion section was shortened and made more concise, as requested by both Reviewers. The length was reduced by approximately 20%.

We thank the Editor and Reviewers for their constructive and helpful comments and look forward to future submissions.

Anne-Mari Mustonen & co-authors
